# Supplementary material for: Effects of the Kampo medicine Yokukansan for perioperative anxiety and postoperative pain in women undergoing breast surgery: A randomized, controlled trial
Source: PLoS One. 2021 Nov 24;16(11):e0260524. doi: 10.1371/journal.pone.0260524 (PMC8612547; doi:10.1371/journal.pone.0260524)
Supplement: S1 File — (DOCX) [file pone.0260524.s002.docx]

**Research Protocol**

Niigata University Ethical Review Committee approved version Created on 2017.06.15

Clinical Trials Act compatible version, 1st edition Created on 2019.01.09

Clinical Trials Act compatible version, 2nd edition Created on 2019.02.15

Clinical Trials Act compatible version, 3rd edition Created on 2019.03.06

Clinical Trials Act compatible version, 4th edition Created on 2019.07.01

**1. [Research subject name]**

A prospective randomized controlled trial to investigate the effect of yokukansan on perioperative anxiety and pain

**2. [Organization of the research]**

This study will be conducted in compliance with the ethical principles, clinical research methods, related notices and research plan stipulated in the Declaration of Helsinki (revised 2013).

① [Principal Investigator]

Hiroshi Baba: Professor, Department of Anesthesiology, Niigata University Medical and Dental Hospital

② [Research Member]

Yoshinori Kamiya: Associate professor, Department of Anesthesiology, Niigata University Medical and Dental Hospital

Moegi Tanaka: Staff anesthesiologist, Department of Anesthesiology, Niigata University Medical and Dental Hospital

Chieko Shibue: Staff anesthesiologist, Department of Anesthesiology, Niigata Cancer Center Hospital

Misako Takamatsu: Staff anesthesiologist, Department of Anesthesiology, Niigata Cancer Center Hospital

Tsunehiko Tanaka: Associate professor, Educational Psychology Course, Faculty of Education, Niigata University

③ [Place of research]

Niigata University Medical and Dental Hospital Operating Room, Ward, and Anesthesiology Outpatient Examination Room

Niigata Cancer Center Niigata Hospital Breast Surgery, Ward, Operating Room

④ [Matters related to persons engaged in clinical research other than the PI]

Data management manager

Moegi Tanaka: Staff anesthesiologist, Department of Anesthesiology, Niigata University Medical and Dental Hospital

Monitoring manager

Tatsunori Watanabe: Specially appointed Lecturer, Department of Anesthesiology, Uonuma Institute of Community Medicine, Niigata University Medical and Dental Hospital

Audit Officer

Kenta Furutani: Lecturer, Department of Anesthesiology, Niigata University Medical and Dental Hospital

Person in charge of statistical analysis

Tsunehiko Tanaka: Associate professor, Educational Psychology Course, Faculty of Education, Niigata University

**3. [Purpose and significance of research]**

It is known that excessive perioperative anxiety causes an increase in blood pressure and tachycardia. On the other hand, reducing excessive perioperative anxiety is known to be shortened the postoperative course and hospitalization period and postoperative pain. Benzodiazepine anxiolytics have traditionally been used as pre-anesthetic medications to reduce perioperative anxiety [1], but benzodiazepines can cause excessive sedation, and their use tends to be withheld [2]. Moreover, severe postoperative pain can be a risk factor for postoperative chronic pain due to the delay in motor function recovery that inhibits rehabilitation [3]. Anticonvulsants such as pregabalin and various anxiolytics for perioperative anxiety have been tried as measures against postoperative pain, but there are problems such as ineffectiveness and strong side effects.

Yokukansan is a type of Chinese herbal medicine that has been used for night crying, and hysteria in children but recently is used for dementia-associated symptoms [4] and neuropathic pain [5]. Yokukansan is thought to have a reducing effect on excitability and some effect on pain and emotions. The applicant has also been prescribing yokukansan for patients with neuropathic pain or increased pain due to anxiety at an outpatient clinic. A study examining the effects of yokukansan on perioperative anxiety and sedation levels [6] suggests that yokukansan has the effect of suppressing anxiety while minimizing sedation. No clinical studies have been conducted to investigate whether or not it is effective for postoperative pain. We planned this study because we thought that the anxiolytic and analgesic effects of yokukansan might also benefit perioperative patients.

[1] J. K. Carroll E, et al, *British Journal of Nursing*. 2012; 21(8):479-483

[2] P.F. White, B. et al, *Anesthesia and Analgesia*. 2009; 108(4)1140-1145

[3] Patricia R. Pinto, et al, Journal of pain research. 2012;13(11)1045-1057

[4] Matsunaga S, et al, J Altzheimers Dis. 2016;54(2) 635-43

[5] Nakamura Y, et al, Masui. 2009; 58(10) 1248-55

[6] Young-Chang Arai, et al, Evidence based Complementaly and Alternative Medicine.2014; ID965045

**4. [Research method and period]**

① Research implementation period

From November 1, 2017 to March 31, 2021.

② Information on drugs used

Name: Tsumura Yokukansan (Product number: 054)

Route of administration, dosage and administration: Oral, 1 packet (2.5 g) at a time

Dosage form: Extract granules

③ Research subjects

Patients aged 20 to 60 years who undergo breast cancer surgery at Niigata University Medical and Dental Hospital or Niigata Prefectural Cancer Center Niigata Hospital and undergo total mastectomy and sentinel lymph node biopsy, or partial mastectomy and sentinel lymph node biopsy (Details will be described later).

④ Research method

Multicenter / Open Label / Superiority test by forced random sampling

[1] A pre-treatment questionnaire survey will be conducted on all subjects, and the age, BMI, surgical history, and general status (American Society of Anesthesiologists-Physical Status: ASA-PS, ECOG-PS) will be recorded in advance. Also, before taking the study drug, record the measurement of amylase in saliva, the Hospital anxiety and depression scale (HADS), and the State Trait Anxiety Inventory (STAI).

[2] Subjects are assigned to two groups, one with yokukansan (intervention) and the other without (control), according to the results of randomization. The intervention group should be given 1 packet (2.5 g) of TSUMURA Yokukansan before sleep on the day before surgery and 1 packet (2.5 g) 2 hours before entering the operating room on the day of surgery. For the purpose of maintaining the same quality between the two groups, a forced allocation algorithm is created, and if a bias is confirmed between the groups, forced allocation is performed based on age, surgical history, etc.

[3] Subjects in the intervention group take 1 packet of yokukansan orally 2 hours before entering the operating room on the day of surgery, and then all participants are measured salivary amylase in the operating room. Anesthetized by general anesthesia in the operating room and undergo total or partial mastectomy and sentinel lymph node biopsy

[4] Record salivary amylase measurements, HADS, STAI, visual analog scale of pain: VAS, QOR (Quality of recovery), and the presence or absence of analgesic use 24 hours after surgery.

| 24-hour before surgery | Salivary amylase  HADS STAI |
| --- | --- |
| Just before surgery | Salivary amylase |
| 24-hour after surgery | Salivary amylase  HADS STAI  Visual analog scale for postoperative pain  QOR  Rescue analgesics usage |

※The allowable range is ± 12 hours before and 24 hours after surgery.

⑤ Sample size

In this study, we plan to perform Bayesian analysis. In this method, the posterior probability of the response rate is calculated by an interim analysis, and the calculated posterior probability is monitored. If it is judged that the effect is not as good as that of untreated, the test should be discontinued. Is planned. In addition, since the posterior predictive distribution is obtained based on the data obtained from the interim analysis, it is possible to judge whether the additional data follows the distribution, and it is possible to save the number of cases by performing this procedure. Is known to be. As an interim analysis, an exploratory analysis will be performed when 20 cases in a group are reached based on reference [1]. Recalculate the required number of cases based on the results of the interim analysis. Regarding the design of the number of cases, referring to the previous study, 1-β was 80%, the significance level was 0.05, and the success rate of the intervention group (yokukansan was expected to be taken preoperatively and preoperatively: impression at the outpatient clinic of pain clinic) is set to 75%. About 40 cases are expected in each group. The clinically meaningful difference is defined as σ = 0.3.

[1]Young-Chang Arai, et al, Evidence based Complementaly and Alternative Medicine.2014; ID965045

⑥ Study endpoints

Primary endpoint: Salivary amylase level

Secondary endpoint: HADS STAI QOR Pain VAS

⑦ Analysis method

Statistical analysis methods are planned to be performed by the maximum likelihood estimation method and the Bayesian statistical method. All analysis is performed by a person in charge of analysis different from that of the principal investigator.

For maximum likelihood estimation methods, t-tests that do not correspond to continuous variables, Fisher's exact test for scale classification, and Mann-Whitney U test for continuous variables or ordinal variables that do not follow a normal distribution such as VAS. A test will be used to determine P <0.05 as significant.

For Bayesian statistics, the difference between two independent groups is estimated for the primary endpoint. A pre-analysis will be conducted based on the uniform distribution obtained in the exploratory test and the data of 20 subjects, and the obtained distribution will be used as the prior distribution to generate and verify the posterior distribution using the data of 40 subjects in each group.

**5. [Policies for selecting research subjects]**

(1) Research subjects

① Patients aged 20 to 60 years who have been diagnosed with breast cancer and undergo total mastectomy and sentinel lymph node biopsy, or partial mastectomy and sentinel lymph node biopsy at Niigata University Medical and Dental Hospital or Niigata Prefectural Cancer Center Niigata Hospital

② Patients who received written and oral explanations regarding this study and showed their consent to participate in writing.

(2) Exclusion criteria

① Patients with preoperative condition classification (ASA-PS) 4 or higher by the American Society of Anesthesia

② Patients with BMI (Body Mass Index ≥ 30)

③ Patients who already have pain that requires oral painkillers somewhere in the body except for the primary disease

④ Those who are allergic to the drug used

⑤ Persons with hypokalemia

⑥ Patients taking anti-anxiety drugs and antipsychotic drugs

⑦ Patients who are already taking painkillers including opioids

⑧ Patients who have already taken some Chinese medicine

⑨ Patients who have difficulty communicating (dementia, mental retardation, mental illness, do not understand Japanese, etc.)

⑩ Other patients who are judged to be inappropriate by the principal investigator and the investigator

(3) Cancellation criteria

① When the research subject offers to decline participation in the research or withdraws consent

② When the entire research is canceled

③ When the result of intraoperative sentinel lymph node biopsy is positive and axillary lymph node dissection is performed

④ When the person in charge of research determines that it is appropriate to discontinue the research for other reasons.

(4) Voluntary research cooperation and freedom of withdrawal

Participation in the study is decided by the patient's free will, and it is possible to withdraw participation at any time. If the patient withdraws from the study before the start of oral administration, it will be excluded from this study. Patients who refuse to take the second dose after the first dose and withdraw from the study are also excluded from this study. Explain that withdrawal of research participation does not result in any disadvantage in subsequent treatment.

**6. [Basis for scientific rationality of research]**

If this study proves that yokukansan is effective for perioperative anxiety and pain, it will be possible to improve the patient's perioperative quality of life with inexpensive Chinese herbs with few side effects.

**7. [Procedures for receiving informed consent, etc.]**

To explain the research, use the research manual (attached to the materials) to participate in the clinical research, regarding the purpose, method and funding source of the clinical research, possible conflicts of interest, involvement with related organizations such as researchers, and so on. A written and oral explanation of the expected benefits and possible risks, the inevitable unpleasant condition, the response after the end of the clinical study, the presence or absence of compensation for the clinical study, and other necessary matters. Please sign the attached material). Strict privacy is maintained for the patient, participation in the study is not obligatory, refusal to participate in the study, or withdrawal of consent in the middle does not disadvantage the subsequent treatment. Explain the warranty. When a participant withdraws his / her consent, he / she confirms his / her intention to withdraw his / her consent by using the consent withdrawal form (attached to the material).

**8. [Handling of personal information, etc.]**

All persons in charge of this study shall comply with the "Declaration of Helsinki (revised in October 2013)" and the Clinical Research Law. When publishing the results of the study, do not include information that can identify the subject. In addition, we will not use the materials of the subjects obtained in the research other than the purpose of the research.

**9. [Burden on research subjects and expected risks and benefits, comprehensive evaluation of these, and measures to minimize the burden and risks]**

Patients in the control group of this study do not take any medication and therefore do not suffer any benefit or disadvantage. On the other hand, patients in the intervention group have the disadvantages of having to take the drug even if it is difficult to take it, and the side effects of Chinese herbs may occur.

The side effects of yokukansan have been reported to be hypokalemia, gastrointestinal disorders (diarrhea and nausea), liver dysfunction, etc., but the frequency is stated to be less than 0.1-5% in the package insert. All of them are side effects of long-term continuous use (reported in 13 days at the earliest). Most of them occur in the elderly aged 65 and over. Since this time, the drug was taken only twice, it is highly unlikely that such side effects will occur. In the unlikely event that yokukansan causes health problems, the researcher will deal with it in good faith and provide appropriate medical care. The cost will be covered by the subject's insurance, and no special compensation will be provided by this study. The collection period for adverse events should be continuous from the acquisition of consent to the end of the study.

**10. [Procedures for collecting, recording, and reporting information on diseases, etc. (including identification of important diseases, etc. that the principal investigator should report to the principal investigator and abnormal values ​​of clinical tests, and requirements and deadlines for reporting) and outbreaks of diseases, etc. Observation period of subjects for later clinical studies]**

As described in the above, the onset of serious complications due to oral administration of Suppression and the appearance of abnormal values ​​in clinical tests are not expected, but complications with clinical symptoms (eg, weakness due to hypokemia, etc.) ), If the length of hospital stay due to breast surgery set by the clinical pass is extended, information will be transmitted from the Niigata University Medical and Dental Hospital and Niigata Prefectural Cancer Center Niigata Hospital Breast Surgery to the doctors in charge of anesthesia at each hospital, and they will be discharged. Until then, the progress of clinical symptoms and test results is observed and recorded.

**11. [Viewing original materials]**

At the conducting medical institution, the principal investigator ensures that all records, including source materials, can be viewed during monitoring, auditing and investigations by accredited clinical research review committees and regulators.

**12. [Method of storing and disposing of samples / information (including materials related to information used in research)]**

The information obtained from this study will be kept strictly until 10 years have passed after all research plans have been completed. Regarding the information obtained at all facilities, all or part of the information that can identify an individual is removed from the personal information and anonymized by numbering. The obtained patient information and questionnaire results will be stored in a locked drawer of the Niigata University Anesthesiology Laboratory in an anesthesiology state and will not be taken out. The correspondence table and patient information will be stored separately, and care will be taken to ensure that information is exchanged between hospitals appropriately. The person in charge of storage shall be Hiroshi Baba, the person in charge of research. When discarding the obtained information, shredder the information left on the paper medium and erase the information left in the file format using data erasure software. The results of this research will be published by presenting them at academic meetings such as academic societies and academic journals, but in that case as well, consideration will be given to not being able to identify individuals, and the results of statistical analysis will be the main focus.

If the research is rejected or the research is discontinued after consenting to the research, all the information on the patients to be studied collected up to that point will be discarded and no further data will be collected.

**Procedures for handling missing, rejected and abnormal data**

If there is missing data on the primary endpoint, the patient's data will be excluded from the analysis. If it is found that missing data exists in the secondary evaluation item during data analysis, the item is treated as a deletion value and data analysis is performed.

**13. [Contents and methods of reporting to the director of the research institution]**

If we obtain facts or information that impair the ethical validity or scientific rationality of the research, or facts or information that may impair the appropriateness of the research implementation or the credibility of the research results, promptly document the relevant matters and countermeasures. Report. If we become aware of a serious adverse event, we will promptly report it in writing. At the end of the study, report the study results in writing without delay.

**14. [Status of conflicts of interest related to research by research institutes, such as funding sources for research, and conflicts of interest related to research by researchers, etc.]**

① How to raise research funds

Plan to use basic expenses such as university operating expenses in the field of anesthesiology

(2) Items to be confirmed regarding conflicts of interest

None

**15. [Method of disclosing information on research]**

The results of this research will be published by presenting them at academic meetings such as academic societies and academic journals. The outline of this study will be registered in the UMIN clinical trial registration system, which is a public database, before the start of the study, and the registered contents will be updated according to changes in the research plan and the progress of the study. When the research is completed, the results of the research will be registered. The outline, progress, results, etc. of clinical research will be announced on jRCT (<https://jrct.niph.go.jp/>).

**16. [Responding to consultations, etc. from research subjects and related parties]**

Not listed for personal information protection

-------------------------------------------------------------------------------------------------------

**Check the following whether it is applicable or not, and if applicable, describe the details.**

**17. [When receiving informed consent from a surrogate, etc.]**

□ Applicable ・ ■ Not applicable

**18. [When obtaining informed assent]**

□ Applicable ・ ■ Not applicable

**19. [When conducting research in a situation where the research subject is in an urgent and obvious life-threatening situation]**

□ Applicable ・ ■ Not applicable

20. [When the research subject has an economic burden or a reward]

□ Applicable ・ ■ Not applicable

**21. [For studies involving invasive procedure ①]**

■ ︎ Applicable ・ □ Not applicable

This study is not covered by clinical research insurance and does not provide any other compensation. In the event of a health hazard, the investigator will deal with it in good faith and provide appropriate medical care. The cost will be covered by the subject's insurance, and no special compensation will be provided by this study. Explain the above points to the subjects in advance and obtain their consent.

**22. [In the case of research involving invasive procedure ②]**

■ ︎ Applicable ・ □ Not applicable

Hypokemia, gastrointestinal disorders (diarrhea and nausea), and liver dysfunction have been reported as adverse events, but the frequency is stated to be less than 0.1 to 5% in the attached text, all of which are long-term. It is a side effect of continuous use (reported in 13 days at the earliest). Most of them occur in the elderly aged 65 and over. Since this time, the drug was taken only twice, it is highly unlikely that such side effects will occur. In the unlikely event that yokukansan causes health problems, the researcher will deal with it in good faith and provide appropriate medical care. The cost will be covered by the subject's insurance, and no special compensation will be provided by this study.

**23. [In the case of research involving medical practice beyond normal medical care]**

□ Applicable ・ ■ Not applicable

**24. [When there is a possibility that important findings regarding the health of the research subject, genetic characteristics that can be inherited by offspring, etc. may be obtained by conducting the research]**

□ Applicable ・ ■ Not applicable

**25. [When entrusting a part of research-related work]**

□ Applicable ・ ■ Not applicable

**26. [Possibilities for future use of acquired samples and information]**

■ Applicable ・ □ Not applicable

There is a possibility of performing secondary analysis.

**27. [Procedures for monitoring and auditing]**

■ Applicable ・ □ Not applicable

The person in charge of monitoring will investigate that this study is being carried out properly at this hospital, that necessary items are accurately recorded, and that the reliability of the data is sufficiently maintained. If any matter that affects the proper implementation of this research or deviation from the research plan is confirmed, the matter is promptly notified to the principal investigator and the research institution as necessary, and they are reported. Appropriate measures shall be taken to prevent the recurrence of the matter.

The person in charge of audit evaluates the implementation of this research including monitoring and the status of compliance with the ethical guidelines and research implementation plan from an objective standpoint, and conducts the audit.
