# Supplementary material for: Effects of the Kampo medicine Yokukansan for perioperative anxiety and postoperative pain in women undergoing breast surgery: A randomized, controlled trial
Source: PLoS One. 2021 Nov 24;16(11):e0260524. doi: 10.1371/journal.pone.0260524 (PMC8612547; doi:10.1371/journal.pone.0260524)
Supplement: S2 File — (DOCX) [file pone.0260524.s003.docx]

**研究計画書**

新潟大学倫理審査委員会承認版 作成日 2017.06.15

臨床研究法対応版・第1版 作成日2019.01.09

臨床研究法対応版・第2版 作成日2019.02.15

臨床研究法対応版・第3版 作成日2019.03.06

臨床研究法対応版・第4版 作成日2019.07.01

**1.【研究課題名】**

周術期の不安や痛みに対する抑肝散の効果を調査するための前向き無作為化比較試験

**2.【研究の実施体制】**

本研究は「ヘルシンキ宣言(2013年改訂)」に規定された倫理的原則、臨床研究法、関連通知並びに本研究計画書を遵守して実施する。

**①【研究責任医師】**

所属　新潟大学医歯学総合病院麻酔科　　　　　職名　教授　　　氏名　馬場　洋

**②【研究分担医師】**

所属　新潟大学医歯学総合病院麻酔科　　　　　職名　准教授　　氏名　紙谷　義孝

所属　新潟大学医歯学総合病院麻酔科　　　　　職名　医員　　　氏名　田中　萌生

所属　新潟県立がんセンター新潟病院麻酔科　　職名　部長　　　氏名　渋江　智栄子

所属　新潟県立がんセンター新潟病院麻酔科　　職名　部長　　　氏名　高松　美砂子

所属　新潟大学人文社会科学系教育学系列　　　職名　准教授　　氏名　田中　恒彦

**③【研究実施の場所】**

新潟大学医歯学総合病院手術室、病棟、および麻酔科外来診察室

新潟県立がんセンター新潟病院乳腺外科、病棟、手術室

④**【研究責任医師以外の臨床研究に従事する者に関する事項】**

データマネージメント担当責任者

田中萌生（新潟大学医歯学総合病院　麻酔科、医員）

モニタリング担当責任者

渡部達範（新潟大学地域医療教育センター魚沼基幹病院　麻酔科、特任講師）

監査担当責任者

古谷健太（新潟大学医歯学総合病院　麻酔科、講師）

統計解析担当責任者

田中恒彦（新潟大学人文社会科学系　教育学系列、准教授）

**3.【研究の目的及び意義】**

　周術期の過度の不安は血圧上昇や頻脈などをひきおこすことが知られているが、周術期の過度の不安の低減が良好な術後経過や入院期間の短縮、また術後痛が慢性疼痛へ移行するリスクの軽減に繋がることが示されている。周術期の不安を抑えるために，これまで麻酔前投薬としてベンゾジアゼピン系抗不安薬が使用されていた[1]が、ベンゾジアゼピン系薬物は過度の鎮静を引き起こすことがあり、患者が手術室まで歩行できなくなったり、患者氏名の確認ができなくなったりと不都合なことも多く、使用は控えられる傾向にある[2]。また、術後痛は、それによって引き起こる離床遅延による運動機能回復の遅れに影響することや、術後痛が術後慢性痛のリスクファクターとなりうるとする研究[3]もある。そして術後痛への対策としてプレガバリンなどの抗けいれん薬、周術期の不安に対して様々な抗不安薬などが試されているが、効果がなかったり、副作用が強く出現したりと問題が多い。

抑肝散は漢方薬の一種であり、これまで小児の夜泣きやヒステリーなどに用いられてきたが、認知症周辺症状に対する効果[4]や神経障害性疼痛などへの効果[5]とその作用機序が明らかになりつつあり、痛みと情動に何らかの作用を及ぼすと考えられている漢方薬である。申請者もペインクリニック外来での診察で抑肝散を神経障害性疼痛や，不安により痛みが増強している患者に対して処方することがある。周術期の不安と鎮静レベルに対する抑肝散の効果を検討した研究[6]では、抑肝散は鎮静を最小限に抑えつつ不安を抑える効果があると示唆しているが、抑肝散が術後痛に有効であるかを検討した臨床研究は未だなされていない。この抑肝散の抗不安作用と鎮痛作用は周術期の患者にも利益をもたらすのではないかと考え、この研究を計画した。

参考文献

[1] J. K. Carroll E, et al, *British Journal of Nursing*. 2012; 21(8):479-483

[2] P.F. White, B. et al, *Anesthesia and Analgesia*. 2009; 108(4)1140-1145

[3] Patricia R. Pinto, et al, Journal of pain research. 2012;13(11)1045-1057

[4] Matsunaga S, et al, J Altzheimers Dis. 2016;54(2) 635-43

[5] Nakamura Y, et al, Masui. 2009; 58(10) 1248-55

[6] Young-Chang Arai, et al, Evidence based Complementaly and Alternative Medicine. 2014; ID965045

**4.【研究方法及び期間】**

①研究実施期間

　研究実施期間は2017年11月１日〜2021年3月31日までの期間を予定する。

②使用医薬品情報

　名称：ツムラ抑肝散

　投与経路、用法・用量：経口、１回１包(2.5g)

剤形：エキス顆粒

　製品番号：054

③研究対象者

　新潟大学医歯学総合病院または新潟県立がんセンター新潟病院乳腺外科で乳がん手術を受ける20〜60歳患者で、乳房全摘及びセンチネルリンパ節生検、もしくは乳房部分切除及びセンチネルリンパ節生検を受ける患者（詳細は後述する）。

④研究方法

多施設／オープンラベル／強制無作為割り付けによる優越性試験

［1］被験者全例に治療前のアンケート調査を行い、年齢、BMI、手術歴、全身状態(American Society of Anesthesiologists-Physical Status: ASA-PS, ECOG-PS)を予め記録する。また、試験薬を内服する前に唾液中アミラーゼの測定と、不安とうつ状態の尺度(Hospital anxiety and depression scale: HADS)、状態—特性不安調査 (State Trait Anxiety Inventory: STAI)を記録する。抑肝散が効果的な人の特徴に、「舌の提出が悪い」ということが最近学会等で発表おり、漢方の教科書にも記載されるようになったため、舌だしの結果を収集すると同時に、ストレス指標の一つとして、脈拍数（心拍数）を患者が記載するアンケート用紙に記録者が記載する。

［2］本研究は中央割付で実施する。具体的な手続きとしては，被験者を、研究代表者の所属する部署とは異なる部署にて行われた無作為化の結果に合わせて抑肝散を内服させる(介入)群と内服させない(対照)群の二群に割り付けする。介入群には術前日の眠前にツムラ抑肝散を１包(2.5g)、手術当日の手術室入室２時間前に１包(2.5g)の計２回内服させる。なお，両群間を等質に保つことを目的に，強制割り付けアルゴリズムを作成し，群間に偏りが確認された場合年齢，手術歴などをもとに強制割り付けを行う。

［3］被験者全例に手術当日の手術室入室２時間前に内服後、手術室で唾液中アミラーゼ測定を行う。手術室で全身麻酔によって麻酔を受け、通常の乳房全摘もしくは乳房部分切除と、センチネルリンパ節生検を受ける。

［4］手術後24時間後に再び唾液中アミラーゼ測定値、HADS、STAI、痛みのVisual Analog Scale: VAS、QOR(Quality of recovery)、術後24時間での鎮痛薬使用の有無を記録する。

| 術前２４時間 | 唾液中アミラーゼ値  HADS STAI |
| --- | --- |
| 術直前 | 唾液中アミラーゼ値 |
| 術後２４時間 | 唾液中アミラーゼ値  HADS STAI  痛みのVAS  QOR  鎮痛薬使用の有無 |

※術前、術後24時間は±12時間を許容範囲とする。

　⑤サンプルサイズ

　本研究ではベイジアン解析を行うことを予定している。この方法では中間解析にて反応率の事後確率を算出し、その上で算出された事後確率のモニタリングを行い、未治療と比較して効果がなさそうと判断されれば試験は中止となることを予定している。また、中間解析から得られたデータをもとに事後予測分布が得られるので追加データがその分布に従うかを判断することが可能であり、この手続きを行うことにより症例数を節約することが可能であることが知られている。中間解析として、参考文献[1]をもとに一群20例に到達した時点での探索的解析を行う。中間解析の結果をもとに必要症例数の再計算を行う。例数設計については先行研究を参考に，1-βを80％，有意水準を0.05，介入群成功率（抑肝散を術前日、術前に内服できると見込める率：ペインクリニック外来での印象から大まかに推定）を75％，統制群成功率を20％と設定。各群40例程度を見込んでいる。臨床的に意味がある差としてσ＝0.3と定める。

[1]Young-Chang Arai, et al, Evidence based Complementaly and Alternative Medicine.2014; ID965045

⑥検査・調査の項目

主要評価項目：唾液中アミラーゼ値

副次的評価項目：HADS STAI QOR 痛みのVAS

⑦解析方法

統計学的解析法は最尤推定法による検定とベイズ統計法による検定を行うことを予定している。全ての解析は，研究代表者とは異なった解析担当者が行い，研究代表者が解析データ，解析方法に触れることはない。

最尤推定法については，連続変数に対しては対応のないt検定、尺度分類などにはFisherの正確確率検定、VASなどの正規分布に従わない連続変数または順序変数にはMann-WhitneyのU検定を用い、P<0.05を有意と判定する予定である。

ベイズ統計法については，一次評価項目について，独立した二群の差の推測を行う。その際，探索的試験にて得られた一様分布と20名のデータを元にプレ解析を行い得られた分布を事前分布とし，各群40名のデータを用いて事後分布を生成し，検証を行う。

**5.【研究対象者の選定方針】**

(1) 研究の対象者

①乳がんと診断され、新潟大学医歯学総合病院または新潟県立がんセンター新潟病院において乳房全摘及びセンチネルリンパ節生検、もしくは乳房部分切除及びセンチネルリンパ節生検を受ける20歳以上60歳以下の患者

②本研究に関して書面と口頭による説明を受け、書面で参加の同意を示した患者

(2) 除外基準

1. 米国麻酔学会術前状態分類(ASA-PS)4以上の患者
2. BMI(Body Mass Index≧30の患者
3. 原疾患を除きすでに体のどこかに鎮痛薬の内服を必要とする痛みがある患者
4. 使用する薬剤に対してアレルギーを有する者
5. 低K血症の認められる者
6. 抗不安薬、向精神病薬を内服している患者
7. オピオイドを含めた鎮痛薬をすでに常用している患者
8. 何らかの漢方薬をすでに内服している患者
9. 意思疎通が難しい患者(認知症、精神発達遅滞、精神疾患、日本語を解さないなど)
10. その他、研究責任医師、研究分担医師が不適切と判断した患者

(3) 中止基準

① 研究対象者から研究参加の辞退の申し出や同意の撤回があった場合

② 本研究全体が中止された場合

③ 術中センチネルリンパ節生検の結果陽性となり、腋窩リンパ節郭清が施行された場合

④その他の理由により、研究担当者が研究の中止が適当と判断した場合

(4) 研究協力の任意性と撤回の自由

　研究への参加は患者の自由意思で決定され、いずれの時点においても参加を撤回することは可能である。患者が内服開始前に研究参加を撤回した場合は、本研究から除外する。１回目の内服後に、患者が２回目の内服を拒否し、研究参加を撤回した場合も本研究から除外する。研究参加を撤回することによって、その後の治療で不利益をこうむることはないことを説明する。

**6.【研究の科学的合理性の根拠】**

　この研究により、抑肝散が周術期の不安や痛みに効果的であると証明できると、安価で副作用の少ない漢方薬によって患者の周術期QOLを向上させることが可能となる。

**7.【インフォームド・コンセントを受ける手続き等】**

　研究についての説明は、研究説明書（資料添付）を用いて当該臨床研究の目的、方法及び資金源、起こりうる利害の衝突、研究者等の関連組織との関わり、当該臨床研究に参加することにより期待される利益及び起こりうる危険、必然的に伴う不快な状態、当該臨床研究終了後の対応、臨床研究に伴う補償の有無その他必要な事項について、文書および口頭で説明した上で同意書（資料添付）に署名を頂く。患者に対して、プライバシーは厳格に保たれること、研究の参加は義務ではないこと、研究への参加拒否、または途中で参加同意を撤回しても、その後の治療に不利益にならないことの保証を説明する。参加者が同意を撤回した際には、同意撤回書（資料添付）を用いて同意撤回の意思を確認する。

**8.【個人情報等の取り扱い】**

本研究のすべての担当者は、「ヘルシンキ宣言(2013年10月改訂)」および臨床研究法を遵守して実施する。研究の結果を公表する際は被験者を特定できる情報は含まないようにする。また研究の目的以外に、研究で得られた被験者の資料などを使用しない。

**9.【研究対象者に生じる負担並びに予測されるリスク及び利益，これらの総合的評価並びに当該負担及びリスクを最小化する対策】**

この研究の対照群にあたる患者は薬を内服しないため、いかなる利益も不利益も被らない。一方介入群にあたる患者は、不利益として、薬が内服し辛い場合も内服しなければいけないことと、漢方薬の副作用が生じる可能性が挙げられる。

抑肝散の副作用としては、低K血症、胃腸障害（下痢や嘔気）、肝機能障害等が報告されているが、頻度としては、添付文書にはいずれも0.1〜5%未満と記載されており、いずれも長期連用（最短で１３日での報告がある）での副作用である。またそのほとんどが65歳以上の高齢者で発生している。今回は２回のみの内服のため、このような副作用が出現する可能性は極めて低いと考えられる。万一、抑肝散によって健康被害が生じた場合、研究担当者は誠意を持って対処し、適切な医療を提供する。その費用は被験者の保険診療で行い、本研究による特別の補償は行わない。有害事象の収集期間は同意取得から研究終了まで継続して収集できるようにする。

**10．疾病等の情報収集、記録及び報告に関する手順（研究責任医師が研究代表医師に報告すべき重要な疾病等及び臨床検査の異常値の特定並びに報告の要件及び期限を含む。）及び疾病等発生後の臨床研究の対象者の観察期間**

９．に記載のごとく抑肝散の内服による重篤な合併症の発症及び臨床検査の異常値の出現は想定していないが、臨床症状を伴うような合併症（例：低K血症による脱力など）によりクリニカルパスで設定された乳腺手術による入院期間が延長した場合、新潟大学医歯学総合病院及び新潟県立がんセンター新潟病院乳腺外科からそれぞれの病院の麻酔科担当医師に情報が伝達され、退院までの間臨床症状や検査結果などの経過が観察・記録される

**11．原資料等の閲覧**

実施医療機関において、研究責任医師はモニタリング、監査及び認定臨床研究審査委員会及規制当局による調査の際に、原資料等全ての記録を閲覧できることを保証する。

**12.【試料・情報（研究に用いられる情報に係る資料を含む。）の保管及び廃棄の方法】**

この研究で得られた情報は、すべての研究の計画が終了し，10年が経過するまで厳重に保管する。すべての施設で得られた情報に関して、個人情報から個人を識別することができる情報の全部又は一部を取り除き、番号を付すことによって匿名化する。得られた患者情報およびアンケート結果は匿名化の状態で、新潟大学麻酔科学研究室の施錠された引き出しに保管を行い、外部への持ち出しは行わない。対応表と患者情報は別々に保管し、病院間の情報の授受は適切になされるよう配慮する。保管責任者は、研究責任者馬場洋とする。得られた情報を破棄する場合は、紙媒体で残されたものについてはシュレッダー処理、ファイル形式で残されたものはデータ消去ソフトを用いて消去する。本研究の結果は学会などの学術集会や学術雑誌などで発表することにより公表するが、その際も個人が特定できないように配慮し、統計解析結果を中心とする。

研究同意後に不採用、研究中止となった場合は、その時点まで収集した研究対象患者の情報すべてを破棄し、それ以降のデータ収集も行わない。

**欠落、不採用及び異常データの取扱いの手順**

主要評価項目の欠落データがある場合は当該患者のデータは解析から除外する。副次評価項目においてデータ解析の際に欠落データが存在することが判明した場合、当該項目については欠失値として扱いデータ解析を行う。

**13.【研究機関の長への報告内容及び方法】**

研究の倫理的妥当性若しくは科学的合理性を損なう事実や情報、研究実施の適正若しくは研究結果の信頼性を失うおそれのある事実や情報を得た場合、該当事項および対処方法を速やかに文書により報告する。重篤な有害事象の発生を知った場合は速やかに文書により報告する。研究を終了する際には、研究結果を遅延なく文章により報告する。

**14.【研究の資金源等，研究機関の研究に係る利益相反及び個人の収益等，研究者等の研究に係る利益相反に関する状況】**

**①研究資金の調達方法**

　麻酔科学分野の大学運営費等の基幹的経費を使用する予定

**②利益相反についての確認事項**

　なし

**15.【研究に関する情報公開の方法】**

本研究の結果は学会などの学術集会や学術雑誌などで発表することにより公表する。この研究の概要は，研究を開始する前に公開データベースであるUMIN臨床試験登録システムに登録し，研究計画書の変更及び研究の進み具合に応じて登録内容を更新する。また研究を終了したときは，研究の結果を登録する。臨床研究の概要、進捗状況、結果などについてはjRCT(<https://jrct.niph.go.jp/>)において公表する。

**16.【研究対象者等及びその関係者からの相談等への対応】**

個人情報保護のため記載せず

**-------------------------------------------------------------------------------------------------------------------------**

**以下は該当・非該当をチェックし，該当する場合は詳細を記載すること。**

**17.【代諾者等からインフォームド・コンセントを受ける場合】**

**□該当・■非該当**

**18.【インフォームド・アセントを得る場合】**

**□該当・■非該当**

**19.【研究対象者に緊急かつ明白な生命の危機が生じている状況における研究を実施しようとする場合】**

**□該当・■非該当**

**20.【研究対象者等に経済的負担又は謝礼がある場合】**

**□該当・■非該当**

**21.【侵襲（軽微な侵襲を除く。）を伴う研究の場合①】**

**■︎該当・□非該当**

本研究は、臨床研究保険に加入しておらず、それ以外の補償も用意していない。健康被害が発生した場合、研究担当者は誠意を持って対処し、適切な医療を提供する。その費用は被験者の保険診療で行い、本研究による特別の補償は行わない。以上の点をあらかじめ被験者に説明し、同意を得る。

**22.【侵襲を伴う研究の場合②】**

**■︎該当・□非該当**

有害事象として低K血症、胃腸障害（下痢や嘔気）、肝機能障害が報告されているが、頻度としては、添付文章にはいずれも0.1〜5%未満と記載されており、いずれも長期連用（最短で１３日での報告がある）での副作用である。またそのほとんどが65歳以上の高齢者で発生している。今回は２回のみの内服のため、このような副作用が出現する可能性は極めて低いと考えられる。万一、抑肝散によって健康被害が生じた場合、研究担当者は誠意を持って対処し、適切な医療を提供する。その費用は被験者の保険診療で行い、本研究による特別の補償は行わない。

**23.【通常の診療を超える医療行為を伴う研究の場合】**

**□該当・■非該当**

**24.【研究の実施に伴い，研究対象者の健康，子孫に受け継がれ得る遺伝的特徴等に関する重要な知見が得られる可能性がある場合】**

**□該当・■非該当**

**25.【研究に関する業務の一部を委託する場合】**

**□該当・■非該当**

**26.【取得された試料・情報の将来の使用の可能性】　　　　　　　　　■該当・□非該当**

　　　二次解析を行う可能性がある。

**27.【モニタリング・監査の実施手順】　　　　　　　　　　　　　　　■該当・□非該当**

　モニター担当者は当院において本研究が適切に実施されていること、必要な事項が正確に記録されていること及びデータの信頼性が十分に保たれていることの調査を行う。本研究の適切な実施に影響を及ぼすような事項あるいは研究計画書からの逸脱等が確認された場合は、当該事項は速やかに研究責任者及び必要に応じて当該研究機関に伝えられ、それらの事項の再発を防止するための適切な措置を講じることとする。

　監査担当者はモニタリングを含めた本研究の実施並びに倫理指針及び研究実施計画書等の遵守の状況を客観的な立場から評価を行い、監査を実施する。
