# Supplementary material for: Effects of the Kampo medicine Yokukansan for perioperative anxiety and postoperative pain in women undergoing breast surgery: A randomized, controlled trial
Source: PLoS One. 2021 Nov 24;16(11):e0260524. doi: 10.1371/journal.pone.0260524 (PMC8612547; doi:10.1371/journal.pone.0260524)
Supplement: S3 File — (PDF) [file pone.0260524.s004.pdf]

JP XVII

THE JAPANESE PHARMACOPOEIA

SEVENTEENTH EDITION

Official from April 1, 2016

English Version

THE MINISTRY OF HEALTH, LABOUR AND WELFARE

Notice: This *English Version* of the Japanese Pharmacopoeia is published for the convenience of users unfamiliar with the Japanese language. When and if any discrepancy arises between the Japanese original and its English translation, the former is authentic.

The Ministry of Health, Labour and Welfare Ministerial Notification No. 64

Pursuant to Paragraph 1, Article 41 of the Law on Securing Quality, Efficacy and Safety of Products including Pharmaceuticals and Medical Devices (Law No. 145, 1960), the Japanese Pharmacopoeia (Ministerial Notification No. 65, 2011), which has been established as follows*, shall be applied on April 1, 2016. However, in the case of drugs which are listed in the Pharmacopoeia (hereinafter referred to as ''previous Pharmacopoeia'') [limited to those listed in the Japanese Pharmacopoeia whose standards are changed in accordance with this notification (hereinafter referred to as ''new Pharmacopoeia'')] and have been approved as of April 1, 2016 as prescribed under Paragraph 1, Article 14 of the same law [including drugs the Minister of Health, Labour and Welfare specifies (the Ministry of Health and Welfare Ministerial Notification No. 104, 1994) as of March 31, 2016 as those exempted from marketing approval pursuant to Paragraph 1, Article 14 of the Same Law (hereinafter referred to as ''drugs exempted from approval'')], the Name and Standards established in the previous Pharmacopoeia (limited to part of the Name and Standards for the drugs concerned) may be accepted to conform to the Name and Standards established in the new Pharmacopoeia before and on September 30, 2017. In the case of drugs which are listed in the new Pharmacopoeia (excluding those listed in the previous Pharmacopoeia) and drugs which have been approved as of April 1, 2016 as prescribed under Paragraph 1, Article 14 of the same law (including those exempted from approval), they may be accepted as those being not listed in the new Pharmacopoeia before and on September 30, 2017.

Yasuhisa Shiozaki
The Minister of Health, Labour and Welfare

March 7, 2016

(The text referred to by the term ''as follows'' are omitted here. All of them are made available for public exhibition at the Evaluation and Licensing Division, Pharmaceutical Safety and Environmental Health Bureau, Ministry of Health, Labour and Welfare, at each Regional Bureau of Health and Welfare, and at each Prefectural Office in Japan).

*The term ''as follows'' here indicates the contents of the Japanese Pharmacopoeia Seventeenth Edition from General Notice to Ultraviolet-visible Reference Spectra (pp. 1 – 2405).

CONTENTS

Preface......i
The Japanese Pharmacopoeia, Seventeenth Edition..1
General Notices......1

General Rules for Crude Drugs......5

General Rules for Preparations......7

General Tests, Processes and Apparatus......25
1. Chemical Methods
1.01 Alcohol Number Determination......25
1.02 Ammonium Limit Test......27
1.03 Chloride Limit Test......28
1.04 Flame Coloration Test......28
1.05 Mineral Oil Test......28
1.06 Oxygen Flask Combustion Method......28
1.07 Heavy Metals Limit Test......29
1.08 Nitrogen Determination (Semimicro-Kjeldahl Method)......30
1.09 Qualitative Tests......31
1.10 Iron Limit Test......36
1.11 Arsenic Limit Test......37
1.12 Methanol Test......38
1.13 Fats and Fatty Oils Test......38
1.14 Sulfate Limit Test......40
1.15 Readily Carbonizable Substances Test......41
2. Physical Methods
Chromatography
2.01 Liquid Chromatography......41
2.02 Gas Chromatography......44
2.03 Thin-layer Chromatography......46
2.04 Amino Acid Analysis of Proteins......46
Spectroscopic Methods
2.21 Nuclear Magnetic Resonance Spectroscopy......47
2.22 Fluorometry......49
2.23 Atomic Absorption Spectrophotometry......49
2.24 Ultraviolet-visible Spectrophotometry......51
2.25 Infrared Spectrophotometry......52
Other Physical Methods
2.41 Loss on Drying Test......53
2.42 Congealing Point Determination......54
2.43 Loss on Ignition Test......54
2.44 Residue on Ignition Test......55
2.45 Refractive Index Determination......55
2.46 Residual Solvents......55
2.47 Osmolarity Determination......61
2.48 Water Determination (Karl Fischer Method)......62
2.49 Optical Rotation Determination......65
2.50 Endpoint Detection Methods in Titrimetry......66
2.51 Conductivity Measurement......68
2.52 Thermal Analysis......69
2.53 Viscosity Determination......71
2.54 pH Determination......74
2.55 Vitamin A Assay......75
2.56 Determination of Specific Gravity and Density......76
2.57 Boiling Point and Distilling Range Test......78
2.58 X-Ray Powder Diffraction Method......79
2.59 Test for Total Organic Carbon......83
2.60 Melting Point Determination......84
2.61 Turbidity Measurement......85
2.62 Mass Spectrometry......86
2.63 Inductively Coupled Plasma-Atomic Emission Spectrometry and Inductively Coupled Plasma-Mass Spectrometry......91
2.64 Glycosylation Analysis of Glycoprotein......95
2.65 Methods for Color Matching......96
3. Powder Property Determinations
3.01 Determination of Bulk and Tapped Densities......98
3.02 Specific Surface Area by Gas Adsorption......100
3.03 Powder Particle Density Determination......103
3.04 Particle Size Determination......104
3.05 Water-Solid Interactions: Determination of Sorption-Desorption Isotherms and of Water Activity......108
4. Biological Tests/Biochemical Tests/ Microbial Tests
4.01 Bacterial Endotoxins Test......110
4.02 Microbial Assay for Antibiotics......114
4.03 Digestion Test......117
4.04 Pyrogen Test......120
4.05 Microbiological Examination of Non-sterile Products......121
4.06 Sterility Test......130
5. Tests for Crude Drugs
5.01 Crude Drugs Test......134
5.02 Microbial Limit Test for Crude Drugs and Preparations containing Crude Drugs as Main Ingredient......138
6. Tests for Preparations
6.01 Test for Metal Particles in Ophthalmic Ointments......147
6.02 Uniformity of Dosage Units......147
6.03 Particle Size Distribution Test for Preparations......150
6.04 Test for Acid-neutralizing Capacity of Gastrointestinal Medicines......150
6.05 Test for Extractable Volume of Parenteral Preparations......150
6.06 Foreign Insoluble Matter Test for Injections......151

6.07 Insoluble Particulate Matter Test for Injections 151
6.08 Insoluble Particulate Matter Test for Ophthalmic Solutions 154
6.09 Disintegration Test 155
6.10 Dissolution Test 157
6.11 Foreign Insoluble Matter Test for Ophthalmic Liquids and Solutions 161
6.12 Methods of Adhesion Testing 161
6.13 Release Test for Preparations for Cutaneous Application 163
7. Tests for Containers and Packing Materials
7.01 Test for Glass Containers for Injections ..166
7.02 Test Methods for Plastic Containers 167
7.03 Test for Rubber Closure for Aqueous Infusions 172
9. Reference Standards; Standard Solutions; Reagents, Test Solutions; Measuring Instruments, Appliances, etc.
Reference Standards
9.01 Reference Standards 174
Standard Solutions
9.21 Standard Solutions for Volumetric Analysis 177
9.22 Standard Solutions 189
9.23 Matching Fluids for Color 191
Reagents, Test Solutions, etc.
9.41 Reagents, Test Solutions 191
9.42 Solid Supports/Column Packings for Chromatography 352
9.43 Filter Papers, Filters for filtration, Test Papers, Crucibles, etc. 354
9.44 Standard Particles, etc. 355
Measuring Instruments and Appliances, Thermometers, etc.
9.61 Optical Filters for Wavelength and Transmission Rate Calibration 355
9.62 Measuring Instruments, Appliances 355
9.63 Thermometers 356

Official Monographs 359
Crude Drugs and Related Drugs 1791

Infrared Reference Spectra 2015–2222

Ultraviolet-visible Reference Spectra 2225–2405

General Information
G1 Physics and Chemistry
Near Infrared Spectrometry 2409
pH Test for Gastrointestinal Medicine 2412
System Suitability 2412
Test for Trace Amounts of Aluminum in Total Parenteral Nutrition (TPN) Solutions 2414
Validation of Analytical Procedures 2415
G2 Solid-state Properties
Laser Diffraction Measurement of Particle Size 2418
Measurement of the Diameter of Particles Dispersed in Liquid by Dynamic Light Scattering 2422
Powder Fineness 2424
Powder Flow 2425
Solid and Particle Densities 2427
G3 Biotechnological/Biological Products
Amino Acid Analysis 2428
Basic Requirements for Viral Safety of Biotechnological/Biological Products listed in Japanese Pharmacopoeia 2435
Capillary Electrophoresis 2447
Isoelectric Focusing 2452
Mass Spectrometry of Peptides and Proteins 2454
Monosaccharide Analysis and Oligosaccharide Analysis/Oligosaccharide Profiling 2456
Mycoplasma Testing for Cell Substrates used for the Production of Biotechnological/ Biological Products 2460
Peptide Mapping 2464
Qualification of Animals as Origin of Animal-derived Medicinal Products provided in the General Notices of Japanese Pharmacopoeia and Other Standards 2467
SDS-Polyacrylamide Gel Electrophoresis 2469
Surface Plasmon Resonance 2474
Total Protein Assay 2478
G4 Microorganisms
Decision of Limit for Bacterial Endotoxins 2481
Disinfection and Decontamination Methods 2481
Media Fill Test (Process Simulation) 2484
Microbial Attributes of Non-sterile Pharmaceutical Products 2486
Microbiological Environmental Monitoring Methods of Processing Areas for Sterile Pharmaceutical Products 2489
Parametric Release of Terminally Sterilized Pharmaceutical Products 2494
Preservatives-Effectiveness Tests 2499
Rapid Counting of Microbes using Fluorescent Staining 2501
Rapid Identification of Microorganisms Based on Molecular Biological Method 2503
Rapid Microbial Methods 2505
Sterilization and Sterilization Indicators 2507
G5 Crude Drugs
Analytical Methods for Aflatoxins in Crude Drug and Crude Drug Preparations 2513
Aristolochic Acid 2515
Notification for the Quantitative Marker Constituents of Crude Drugs and Crude

Drug Preparations 2515
Purity Tests on Crude Drugs using Genetic Information 2516
Quantitative Analytical Technique Utilizing Nuclear Magnetic Resonance (NMR) Spectroscopy and its Application to Reagents in the Japanese Pharmacopoeia.............2519
On the Scientific Names of Crude Drugs listed in the JP 2520
Thin-layer Chromatography for Crude Drugs and Crude Drug Preparations 2534
G6 Drug Formulation
Standard Procedure for Mechanical Calibration of Dissolution Apparatus 2536
Tablet Friability Test 2538
G7 Containers and Package
Basic Requirements and Terms for the Packaging of Pharmaceutical Products..................2538
Basic Requirements for Plastic Containers for Pharmaceutical Use and Rubber Closures for Containers for Aqueous Infusions 2542
G8 Water
Quality Control of Water for Pharmaceutical Use 2543
Water to be used in the Tests of Drugs 2550
G9 Reference Standards
Reference Standards and Reference Materials Specified in the Japanese Pharmacopoeia 2550
G10 Others
Basic Concepts for Quality Assurance of Drug Substances and Drug Products 2553
Basic Concept of Quality Risk Management..2554
International Harmonization Implemented in the Japanese Pharmacopoeia Seventeenth Edition...2557

Appendix
Atomic Weight Table (2010) 2595
Standard Atomic Weights 2010 2596

Index 2599
Index in Latin name 2616
Index in Japanese 2618

PREFACE

The Japanese Pharmacopoeia (JP) is an official document that defines the specifications, criteria and standard test methods necessary to properly assure the quality of medicines in Japan.

Paragraph 2, Article 41 of the Law on Securing Quality, Efficacy and Safety of Products including Pharmaceuticals and Medical Devices stipulates that full-fledged JP revisions shall be presented at least every 10 years. Since the JP 9th edition, full-fledged revisions have been made every 5 years. In addition to the full-fledged revisions, a supplement has been promulgated twice in every 5 years since the JP 12th edition as well as partial revisions have been made as necessary to take account of recent progress of science and in the interests of international harmonization.

The 16th Edition of the JP was promulgated by Ministerial Notification No. 65 of the Ministry of Health, Labour and Welfare (MHLW) on March 24, 2011.

In July 2011, the Committee on JP established the basic principles for the preparation of the JP 17th Edition, setting out the roles and characteristics of the JP, the definite measures for the revision, and the date of the revision.

At the Committee, the five basic principles of JP, which we refer to as the ''five pillars'', were established as follows: 1) Including all drugs which are important from the viewpoint of health care and medical treatment; 2) Making qualitative improvement by introducing the latest science and technology; 3) Promoting internationalization; 4) Making prompt partial revision as necessary and facilitating smooth administrative operation; and 5) Ensuring transparency regarding the revision, and disseminating the JP to the public. It was agreed that the Committee on JP should make efforts, on the basis of these principles, to ensure that the JP is used more effectively in the fields of health care and medical treatment by taking appropriate measurements, including getting the understanding and cooperation of other parties concerned.

It was agreed that the JP should provide an official standard, being required to assure the quality of medicines in Japan in response to the progress of science and technology and medical demands at the time. It should define the standards for specifications, as well as the methods of testing to assure overall quality of all drugs in principle, and it should have a role in clarifying the criteria for quality assurance of drugs that are recognized to be essential for public health and medical treatment. The JP has been prepared with the aid of the knowledge and experience of many professionals in the pharmaceutical field. Therefore, the JP should have the characteristics of an official standard, which might be widely used by all parties concerned, and it should play an appropriate role of providing information and understanding about the quality of drugs to the public. Moreover, as a pharmaceutical quality standard, it should contribute promoting and maintaining of advancedness as well as international consistency and harmonization of technical requirements in the international community. It was also agreed that JP articles should cover drugs, which are important from the viewpoint of health care and medical treatment, clinical performance or merits and frequency of use, as soon as possible after they reach the market.

The target date for the publication of JP 17th Edition (the Japanese edition) was set as April 2016.

JP Expert Committees were originally organized with the following committees: Expert Committee; Sub-expert Committee; Committee on Chemicals; Committee on Antibiotics; Committee on Biologicals; Committee on Crude Drugs; Committee on Pharmaceutical Excipients; Committee on Physico-Chemical Methods; Committee on Drug Formulation; Committee on Physical Methods; Committee on Biological Methods; Committee on Nomenclature for Pharmaceuticals; Committee on International Harmonization; Committee on Pharmaceutical Water and Committee on JP Reference Standards. Furthermore, working groups were established under the Committee on Physico-Chemical Methods; Committee on Drug Formulation and Committee on Biological Methods to expedite discussion on revision drafts. Later, the Expert Committees were reorganized in order to solve technical issues with preparation of JP drafts; the Subcommittee on Manufacturing Process-related Matters was newly established and the Committee on JP Reference Standards was re-formed and renamed Committee on Reference Standards. Moreover, working groups were established under the Committee on Pharmaceutical Excipients and Committee on International Harmonization.

In the Committee on JP, Mitsuru Hashida took the role of chairman from January 2011 to March 2016.

In accordance with the above principles, the committees initiated deliberations on selection of articles and on revisions for General Notices, General Rules for Crude Drugs, General Rules for Preparations,

General Tests, Monographs and so on.

In order to ensure distribution of drugs in the area hit by the 2011 off the Pacific coast of Tohoku Earthquake on March 11, 2011, for those drugs that were distributed by the distributors in the same quake-hit area, the expiry date of interim measure of the Supplement II to the JP 15th Edition under the Ministerial Notification No. 425 of the MHLW dated September 30, 2009 was extended to June 30, 2013 and that of the Partial Revision of the JP 15th Edition under the Ministerial Notification No. 322 of the MHLW dated July 30, 2010 was extended to January 31, 2012, which was promulgated as the partial revision of the preamble of the Ministerial Notification of the JP 16th Edition by Ministerial Notification No. 96 of the MHLW on March 31, 2011, and became effective.

Draft revisions covering subjects in General Notices, General Rules for Crude Drugs, General Rules for Preparations, General Tests and Monographs, for which discussions were finished between April 2010 and March 2012, were prepared for a supplement to the JP 16. They were examined by the Committee on JP in May 2012, followed by the Pharmaceutical Affairs and Food Sanitation Council (PAFSC) in June 2012, and then submitted to the Minister of Health, Labour and Welfare.

The supplement was named ''Supplement I to the JP 16th Edition'', promulgated on September 27, 2012 by Ministerial Notification No. 519 of MHLW, and became effective on October 1, 2012.

Numbers of discussions in the committees to prepare the supplement drafts were as follows: Expert Committee (8); Sub-expert Committee (4), Committee on Chemicals (22), Committee on Antibiotics (5); Committee on Biologicals (9); Committee on Crude Drugs (21); Committee on Pharmaceutical Excipients (12); Committee on Physico-Chemical Methods (14); Committee on Drug Formulation (19); Committee on Physical Methods (7); Committee on Biological Methods (13); Committee on Nomenclature for Pharmaceuticals (7); Committee on International Harmonization (8); and Committee on Pharmaceutical Water (7).

It should be noted that in the preparation of the drafts for the supplement, generous cooperation was given by the Pharmaceutical Technology Committee of the Osaka Pharmaceutical Manufacturers Association, the Pharmacopeia and CMC Committee of the Pharmaceutical Manufacturers' Association of Tokyo, the Tokyo Crude Drugs Association, the International Pharmaceutical Excipients Council Japan, the Japan Kampo Medicines Manufacturers Association, the Japan Flavor and Fragrance Materials Association, the Japan Medicinal Plant Federation, the Japan Pharmaceutical Manufacturers Association, the Parenteral Drug Association Japan Chapter, the Japan Reagent Association, the Japan Oilseed Processors Association, the Home Medicine Association of Japan, and the Association of Membrane Separation Technology of Japan.

In consequence of this revision, the JP 16th Edition carries 1837 articles, owing to the addition of 77 articles and the deletion of 4 articles.

Draft revisions covering subjects, the revision of the General Tests and the revision of the specification of monograph Gelatin connected with the harmonization among the three pharmacopoeias, JP, EP and USP were examined by the Committee on JP in February 2013, followed by PAFSC in April 2013, and then submitted to the Minister of Health, Labour and Welfare.

This revision was promulgated on May 31, 2013 by Ministerial Notification No. 190 of MHLW, and became effective.

Draft revisions covering subjects in General Notices, General Rules for Crude Drugs, General Rules for Preparations, General Tests and Monographs, for which discussions were finished between April 2012 and September 2013, were prepared for a supplement to the JP 16. They were examined by the Committee on JP in October 2013, followed by the PAFSC in December 2013, and then submitted to the Minister of Health, Labour and Welfare.

The supplement was named ''Supplement II to the JP 16th Edition'' and promulgated on February 28, 2014 by Ministerial Notification No. 47 of MHLW, and became effective.

Numbers of discussions in the committees to prepare the supplement drafts were as follows: Expert Committee (5); Sub-committee on Manufacturing Process-related Matters (6); Committee on Chemicals (16); Committee on Antibiotics (3); Committee on Biologicals (8); Committee on Crude Drugs (16); Committee on Pharmaceutical Excipients (12); Committee on Physico-Chemical Methods (9); Committee on Drug Formulation (14); Committee on Biological Methods (13); Committee on Nomenclature for Pharmaceuticals (4); Committee on International Harmonization (10); and Committee on Reference Standards (1).

It should be noted that in the preparation of the drafts for the supplement, generous cooperation was given by the Pharmaceutical Technology Committee of the Osaka Pharmaceutical Manufacturers Association, the Pharmacopeia and CMC Committee of the Pharmaceutical Manufacturers' Association of Tokyo, the Tokyo Crude Drugs Association, the International Pharmaceutical Excipients Council Japan, the Japan Kampo Medicines Manufacturers Association, the Japan Flavor and Fragrance Materials Association, the Japan Medicinal Plant Federation, the Japan Pharmaceutical Manufacturers Association, the Federation of Pharmaceutical Manufacturers' Associ-

ation of Japan, the Parenteral Drug Association Japan Chapter, the Japan Reagent Association, the Japan Oilseed Processors Association, the Home Medicine Association of Japan, the Association of Membrane Separation Technology of Japan, the External Pharmaceutical Association, the Japan Alcohol Association and the Pharmacopoeial Drug Society.

In consequence of this revision, the JP 16th Edition carries 1896 articles, owing to the addition of 60 articles and the deletion of 1 article.

In accordance with the change of the title from Pharmaceutical Affairs Law (Act No. 145 of 1960) to Law on Securing Quality, Efficacy and Safety of Products including Pharmaceuticals and Medical Devices by the Law for Partial Revision of the Pharmaceutical Affairs Law (Act No. 84 of 2013), the partial revision to change from ''Pharmaceutical Affairs Law'' to ''Law on Securing Quality, Efficacy and Safety of Products including Pharmaceuticals and Medical Devices'' in JP General Notices was promulgated on November 21, 2014 by Ministerial Notification No. 439 of MHLW, and became effective.

Draft revisions covering subjects in General Notices, General Rules for Crude Drugs, General Rules for Preparations, General Tests and Monographs, for which discussions were finished between October 2013 and July 2015, were prepared for a supplement to the JP 17. They were examined by the Committee on JP in August 2015, followed by the PAFSC in September 2015, and then submitted to the Minister of Health, Labour and Welfare.

Numbers of discussions in the committees to prepare the supplement drafts were as follows: Expert Committee (7); Sub-committee on Manufacturing Process-related Matters (12); Committee on Chemicals (22); Committee on Antibiotics (8); Committee on Biologicals (11); Committee on Crude Drugs (21); Committee on Pharmaceutical Excipients (10, including working group); Committee on Physico-Chemical Methods (9, including working group); Committee on Drug Formulation (23, including working group); Committee on Physical Methods (7); Committee on Biological Methods (12, including working group); Committee on Nomenclature for Pharmaceuticals (6); Committee on International Harmonization (10, including working group); and Committee on Reference Standards (8).

It should be noted that in the preparation of the drafts for the supplement, generous cooperation was given by the Pharmaceutical Technology Committee of the Osaka Pharmaceutical Manufacturers Association, the Pharmacopeia and CMC Committee of the Pharmaceutical Manufacturers' Association of Tokyo, the Tokyo Crude Drugs Association, the International Pharmaceutical Excipients Council Japan, the Home Medicine Association of Japan, the Japan Kampo Medicines Manufacturers Association, the Japan Flavor and Fragrance Materials Association, the Japan Medicinal Plant Federation, the Japan Pharmaceutical Manufacturers Association, the Federation of Pharmaceutical Manufacturers' Association of Japan, the Parenteral Drug Association Japan Chapter, the Japan Reagent Association, the Japan Oilseed Processors Association, the Association of Membrane Separation Technology of Japan, the External Pharmaceutical Association, the Japan Alcohol Association, and the Pharmacopoeial Drug Society.

In consequence of this revision, the JP 17th Edition carries 1962 articles, owing to the addition of 76 articles and the deletion of 10 articles.

The principles of description and the salient points of the revision in this volume are as follows:

1. The JP 17th Edition comprises the following items, in order: Notification of MHLW; Contents; Preface; General Notices; General Rules for Crude Drugs; General Rules for Preparations; General Tests, Processes and Apparatus; Official Monographs; then followed by Infrared Reference Spectra and Ultraviolet-visible Reference Spectra; General Information; Table of Standard Atomic Weights as an appendix; and a Cumulative Index.

2. The articles in Official Monographs, Infrared Reference Spectra and Ultraviolet-visible Reference Spectra are respectively placed in alphabetical order in principle.

3. The following items in each monograph are put in the order shown below, except that unnecessary items are omitted depending on the nature of the drug:

(1) English title
(2) Commonly used name(s)
(3) Latin title (only for crude drugs)
(4) Title in Japanese
(5) Structural formula or empirical formula
(6) Molecular formula and molecular mass
(7) Chemical name
(8) Chemical Abstracts Service (CAS) Registry Number
(9) Origin
(10) Limits of the content of the ingredient(s) and/or the unit of potency
(11) Labeling requirements
(12) Method of preparation
(13) Manufacturing requirement
(14) Description
(15) Identification tests
(16) Specific physical and/or chemical values
(17) Purity tests
(18) Potential adulteration
(19) Loss on drying or Ignition, or Water
(20) Residue on ignition, Total ash or Acid-insoluble ash

(21) Tests being required for pharmaceutical preparations
(22) Other special tests
(23) Assay
(24) Containers and storage
(25) Shelf life
(26) Others

4. In each monograph, the following physical and chemical values representing the properties and quality of the drug are given in the order indicated below, except that unnecessary items are omitted depending on the nature of drug:

(1) Alcohol number
(2) Absorbance
(3) Congealing point
(4) Refractive index
(5) Osmolar ratio
(6) Optical rotation
(7) Constituent amino acids
(8) Viscosity
(9) pH
(10) Content ratio of the active ingredients
(11) Specific gravity
(12) Boiling point
(13) Melting point
(14) Acid value
(15) Saponification value
(16) Ester value
(17) Hydroxyl value
(18) Iodine value

5. Identification tests comprise the following items, which are generally put in the order given below:

(1) Coloration reactions
(2) Precipitation reactions
(3) Decomposition reactions
(4) Derivatives
(5) Infrared and/or ultraviolet-visible absorption spectrometry
(6) Nuclear magnetic resonance spectrometry
(7) Chromatography
(8) Special reactions
(9) Cations
(10) Anions

6. Purity tests comprise the following items, which are generally put in the order given below, except that unnecessary items are omitted depending on the nature of drug:

(1) Color
(2) Odor
(3) Clarity and/or color of solution
(4) Acidity or alkalinity
(5) Acidity
(6) Alkalinity
(7) Chloride
(8) Sulfate
(9) Sulfite
(10) Nitrate
(11) Nitrite
(12) Carbonate
(13) Bromide
(14) Iodide
(15) Soluble halide
(16) Thiocyanate
(17) Selenium
(18) Cationic salts
(19) Ammonium
(20) Heavy metals
(21) Iron
(22) Manganese
(23) Chromium
(24) Bismuth
(25) Tin
(26) Aluminum
(27) Zinc
(28) Cadmium
(29) Mercury
(30) Copper
(31) Lead
(32) Silver
(33) Alkaline earth metals
(34) Arsenic
(35) Free phosphoric acid
(36) Foreign matters
(37) Related substances
(38) Isomer
(39) Optical isomer
(40) Multimers
(41) Residual solvent
(42) Other impurities
(43) Residue on evaporation
(44) Readily carbonizable substances

7. The following paragraphs were newly added to General Notices:

(1) Paragraph 12: The item ''Manufacturing requirement'' was newly added in monograph in order to specify the requirements that should be noted on manufacturing processes such as control of intermediates and manufacturing processes.
(2) Paragraph 34: The specification for residual solvents was added.
(3) Paragraph 35: The item ''Potential adulteration'' was added in monograph in order to describe control of harmful substances that was intentionally contaminated.
(4) Paragraph 40: The definitions of ''sterility'', ''sterilization'' and ''aseptic technique'' as sterility related terms were added.

8. The following paragraphs of General Notices

were revised:

(1) Paragraph 5: Container under ''Containers and storage'' for preparations (excluding preparations containing crude drugs as main active ingredients) in the monographs were removed from the standards for conformity.

(2) Paragraph 48: The symbols (◇ ◇) were newly added as the ways to indicate the part being not harmonized among three pharmacopoeias in order to distinguish ''JP local requirements'' from ''non-harmonized attributes/provisions among three pharmacopoeias''.

(3) Other descriptions were improved.

9. To Paragraph 1 of General Rules for Crude Drugs the following items were added:

(1) Codonopsis Root
(2) Hedysarum Root
(3) Salvia Miltiorrhiza Root

10. The following paragraphs were newly added to General Rules for Preparations:

''[2] General Notices for Packaging of Preparations'' which describe the fundamental requirements for packaging of preparations in order to improve the terminology, definitions and specifications for packaging of preparation.

11. The General Rules for Preparations was revised as follows in general:

(1) General Notices for Preparations (8): The description of sterile preparations; ''sterile preparations'', ''terminal sterilization'' and ''aseptic processing'' was added.

(2) General Notices for Preparations (10): The description of containers and packaging for preparations was deleted.

(3) Monographs for Preparations (2): The description of ''containers and packaging'' was deleted.

(4) Monographs for Preparations (3): The definition of preparation in single-dose package was described.

(5) Other descriptions were improved.

12. The following items were newly added to General Tests, Processes and Apparatus:

(1) 2.64 Glycosylation Analysis of Glycoprotein
(2) 2.65 Methods for Color Matching
(3) 3.05 Water-Solid Interactions: Determination of Sorption-Desorption Isotherms and of Water Activity
(4) 6.12 Methods of Adhesion Testing
(5) 6.13 Release Test for Preparations for Cutaneous Application

13. The following items in General Tests, Processes and Apparatus were revised:

(1) 2.21 Nuclear Magnetic Resonance Spectroscopy
(2) 2.46 Residual Solvents
(3) 2.49 Optical Rotation Determination
(4) 2.52 Thermal Analysis
(5) 2.60 Melting Point Determination
(6) 3.01 Determination of Bulk and Tapped Densities
(7) 5.01 Crude Drugs Test
(8) 5.02 Microbial Limit Test for Crude Drugs and Preparations containing Crude Drugs as Main Ingredient
(9) 6.02 Uniformity of Dosage Units
(10) 6.05 Test for Extractable Volume of Parenteral Preparations
(11) 6.06 Foreign Insoluble Matter Test for Injections
(12) 9.01 Reference Standards
(13) 9.21 Standard Solutions for Volumetric Analysis
(14) 9.22 Standard Solutions
(15) 9.23 Matching Fluids for Color
(16) 9.41 Reagents, Test Solutions
(17) 9.42 Solid Supports/Column Packings for Chromatography
(18) 9.44 Standard Particles, etc.

14. The following Reference Standards were added:

Cilnidipine
Ciprofloxacin
Citicoline
Diflorasone Diacetate
Eplerenone
Isomalt
Lansoprazole
Medroxyprogesterone Acetate
Miglitol
Mitiglinide Calcium
Montelukast for System Suitability
Montelukast Dicyclohexylamine
Montelukast Racemate for System Suitability
Montelukast Sodium for Identification
Residual Solvents for System Suitability
Residual Solvents Class 1
Residual Solvents Class 2A
Residual Solvents Class 2B
Ribavirin
Silodosin
Valaciclovir Hydrochloride
Voriconazole
Interferon Alfa

15. The following Reference Standards were revised in Japanese title:

Acetanilide for Apparatus Suitability
Acetophenetidine for Apparatus Suitability
Caffeine for Apparatus Suitability
Calcitonin Salmon

Calcium Oxalate Monohydrate for Calibration of Apparatus
Insulin Human
Sulfanilamide for Apparatus Suitability
Sulfapyridine for Apparatus Suitability
Vanillin for Apparatus Suitability

16. The following Reference Standards were deleted from the list of 9.01 Reference Standards:
Griseofulvin
Protamine Sulfate
Serum Gonadotrophin
Siccanin

17. The following substances were newly added to the Official Monographs:
Ampicillin Sodium and Sulbactam Sodium for Injection
Ampiroxicam
Ampiroxicam Capsules
Ascorbic Acid and Calcium Pantothenate Tablets
Candesartan Cilexetil and Hydrochlorothiazide Tablets
Hypromellose Capsules
Pullulan Capsules
L-Carbocisteine Tablets
Cefalexin Combination Granules
Cefoperazone Sodium and Sulbactam Sodium for Injection
Cefpodoxime Proxetil for Syrup
Cilnidipine
Cilnidipine Tablets
Ciprofloxacin
Ciprofloxacin Hydrochloride Hydrate
Citicoline
Diflorasone Diacetate
Diltiazem Hydrochloride Extended-release Capsules
Doxycycline Hydrochloride Tablets
Eplerenone
Eplerenone Tablets
Ethyl Icosapentate Capsules
Felbinac Cataplasm
Felbinac Tape
Fluconazole Injection
Fosfomycin Calcium for Syrup
Haloperidol Injection
Interferon Alfa (NAMALWA)
Interferon Alfa (NAMALWA) Injection
Irbesartan
Isomalt Hydrate
Lansoprazole
Lansoprazole Delayed-release Capsules
Lansoprazole Delayed-release Orally Disintegration Tablets
Levofloxacin Injection
Medroxyprogesterone Acetate
Miglitol
Mitiglinide Calcium Hydrate
Mitiglinide Calcium Tablets
Montelukast Sodium
Montelukast Sodium Chewable Tablets
Montelukast Sodium Tablets
Ozagrel Sodium Injection
Panipenem and Betamipron for Injection
Pioglitazone Hydrochloride and Glimepiride Tablets
Ribavirin
Ribavirin Capsules
Silodosin
Silodosin Tablets
Purified Sodium Hyaluronate Injection
Purified Sodium Hyaluronate Ophthalmic Solution
Sodium L-Lactate Ringer's Solution
Spectinomycin Hydrochloride for Injection
Sultamicillin Tosilate Tablets
Tacrolimus Capsules
Teprenone Capsules
Terbinafine Hydrochloride Tablets
Ticlopidine Hydrochloride Tablets
Trientine Hydrochloride
Trientine Hydrochloride Capsules
Tulobuterol
Tulobuterol Transdermal Tape
Valaciclovir Hydrochloride
Valaciclovir Hydrochloride Tablets
Voriconazole
Voriconazole Tablets
Bofutsushosan Extract
Boiogito Extract
Codonopsis Root
Hedysarum Root
Kamikihito Extract
Salvia Miltiorrhiza Root
Anhydrous Sodium Sulfate
Sodium Sulfate Hydrate
Tokakujokito Extract
Yokukansan Extract

18. The following monographs were revised:
Acetylcysteine
Aciclovir
Alacepril Tablets
Aldioxa Tablets
Alendronate Sodium Hydrate
Alendronate Sodium Tablets
Allopurinol Tablets
Alprostadil Injection
Aminophylline Hydrate
Aminophylline Injection
Amiodarone Hydrochloride
Amiodarone Hydrochloride Tablets
Amlexanox Tablets
Amlodipine Besilate
Amphotericin B for Injection

Amphotericin B Tablets
Ampicillin Sodium
Aprindine Hydrochloride
Arbekacin Sulfate
Argatroban Hydrate
Arsenical Paste
Arsenic Trioxide
Atorvastatin Calcium Hydrate
Atropine Sulfate Hydrate
Auranofin
Azathioprine Tablets
Azelnidipine
Azithromycin Hydrate
Bacampicillin Hydrochloride
Bamethan Sulfate
Benserazide Hydrochloride
Benzylpenicillin Benzathine Hydrate
Benzylpenicillin Potassium
Bepotastine Besilate
Beraprost Sodium
Berberine Chloride Hydrate
Betahistine Mesilate
Betamethasone
Betamethasone Tablets
Betaxolol Hydrochloride
Bifonazole
Brotizolam
Bucillamine Tablets
Bucumolol Hydrochloride
Buformin Hydrochloride Delayed-release Tablets
Buformin Hydrochloride Tablets
Bunazosin Hydrochloride
Bupivacaine Hydrochloride Hydrate
Bupranolol Hydrochloride
Butenafine Hydrochloride
Cadralazine
Calcitonin Salmon
Calcium Folinate
Calcium Paraaminosalicylate Hydrate
Anhydrous Dibasic Calcium Phosphate
Monobasic Calcium Phosphate Hydrate
dl-Camphor
Candesartan Cilexetil
Capsules
Carboplatin
Carvedilol
Cefaclor Combination Granules
Cefalexin Capsules
Cefazolin Sodium Hydrate
Cefcapene Pivoxil Hydrochloride Hydrate
Cefcapene Pivoxil Hydrochloride Tablets
Cefditoren Pivoxil
Cefditoren Pivoxil Tablets
Cefixime Capsules
Cefotiam Hexetil Hydrochloride
Cefpirome Sulfate
Cefpodoxime Proxetil Tablets
Cefroxadine Hydrate
Cefteram Pivoxil Tablets
Ceftriaxone Sodium Hydrate
Celmoleukin (Genetical Recombination)
Cetirizine Hydrochloride Tablets
Cetotiamine Hydrochloride Hydrate
Chloramphenicol Palmitate
Chloramphenicol Sodium Succinate
Chlorphenesin Carbamate Tablets
Cibenzoline Succinate Tablets
Cilostazol Tablets
Cinoxacin
Clarithromycin Tablets
Clebopride Malate
Clocapramine Hydrochloride Hydrate
Clomifene Citrate
Clopidogrel Sulfate
Clopidogrel Sulfate Tablets
Cloxacillin Sodium Hydrate
Codeine Phosphate Hydrate
Colestimide
Colistin Sodium Methanesulfonate
Colistin Sulfate
Cyanocobalamin
Cyproheptadine Hydrochloride Hydrate
L-Cystine
Danazol
Dantrolene Sodium Hydrate
Dextromethorphan Hydrobromide Hydrate
Diethylcarbamazine Citrate Tablets
Diflucortolone Valerate
Dihydrocodeine Phosphate
Dilazep Hydrochloride Hydrate
Docetaxel Hydrate
Docetaxel for Injection
Domperidone
Donepezil Hydrochloride
Dorzolamide Hydrochloride
Doxapram Hydrochloride Hydrate
Doxazosin Mesilate
Doxycycline Hydrochloride Hydrate
Droperidol
Droxidopa
Droxidopa Capsules
Ebastine
Ecabet Sodium Hydrate
Ecothiopate Iodide
Edaravone
Edrophonium Chloride
Edrophonium Chloride Injection
Emedastine Fumarate
Enoxacin Hydrate
Enviomycin Sulfate
Epalrestat
Epalrestat Tablets

Epoetin Alfa (Genetical Recombination)
Epoetin Beta (Genetical Recombination)
Erythromycin Delayed-release Tablets
Estradiol Benzoate Injection (Aqueous Suspension)
Estriol Injection (Aqueous Suspension)
Ethambutol Hydrochloride
Ethanol
Anhydrous Ethanol
Ethanol for Disinfection
Ethylmorphine Hydrochloride Hydrate
Faropenem Sodium Hydrate
Faropenem Sodium Tablets
Felbinac
Fenbufen
Fentanyl Citrate
Fexofenadine Hydrochloride
Fexofenadine Hydrochloride Tablets
Filgrastim (Genetical Recombination)
Filgrastim (Genetical Recombination) Injection
Flecainide Acetate
Flecainide Acetate Tablets
Fluconazole
Fluconazole Capsules
Fludrocortisone Acetate
Fluorescein Sodium
Fluphenazine Enanthate
Flurbiprofen
Flutamide
Flutoprazepam
Fluvoxamine Maleate
Fluvoxamine Maleate Tablets
Fudosteine
Furosemide Tablets
Gabexate Mesilate
Gefarnate
Gelatin
Purified Gelatin
Gentamicin Sulfate
Gliclazide
Glimepiride
Gonadorelin Acetate
Guanethidine Sulfate
Heparin Calcium
Heparin Sodium
Hydrocotarnine Hydrochloride Hydrate
Hydroxocobalamin Acetate
Hydroxypropylcellulose
Hydroxyzine Hydrochloride
Hydroxyzine Pamoate
Hypromellose
Ibuprofen Piconol
Idarubicin Hydrochloride
Imidapril Hydrochloride
Imipenem and Cilastatin Sodium for Injection
Indapamide
Indenolol Hydrochloride
Insulin Glargine (Genetical Recombination)
Insulin Glargine (Genetical Recombination) Injection
Insulin Human (Genetical Recombination)
Insulin Human (Genetical Recombination) Injection
Iohexol
Ipratropium Bromide Hydrate
Ipriflavone
Irsogladine Maleate
Isosorbide Mononitrate 70%/Lactose 30%
Ketoconazole
Kitasamycin Tartrate
Labetalol Hydrochloride Tablets
Lafutidine
Lenograstim (Genetical Recombination)
Levofloxacin Hydrate
Levofloxacin Tablets
Lincomycin Hydrochloride Hydrate
Lobenzarit Sodium
Losartan Potassium
Losartan Potassium Tablets
Losartan Potassium and Hydrochlorothiazide Tablets
Loxoprofen Sodium Tablets
Lysozyme Hydrochloride
Mecobalamin
Mefloquine Hydrochloride
Metenolone Enanthate Injection
Methamphetamine Hydrochloride
Methylcellulose
Methyldopa Hydrate
Methylprednisolone Succinate
Metildigoxin
Metoprolol Tartrate Tablets
Minocycline Hydrochloride Tablets
Mizoribine Tablets
Morphine Hydrochloride Hydrate
Morphine Sulfate Hydrate
Mosapride Citrate Hydrate
Nafamostat Mesilate
Naftopidil
Naftopidil Orally Disintegrating Tablets
Nartograstim (Genetical Recombination)
Nartograstim for Injection (Genetical Recombination)
Nateglinide
Nateglinide Tablets
Nifedipine
Nifedipine Delayed-release Fine Granules
Nizatidine Capsules
Nortriptyline Hydrochloride
Noscapine Hydrochloride Hydrate
Olmesartan Medoxomil
Olopatadine Hydrochloride
Omeprazole
Omeprazole Delayed-release Tablets

Oxycodone Hydrochloride Hydrate
Compound Oxycodone Injection
Compound Oxycodone and Atropine Injection
Ozagrel Sodium for Injection
Panipenem
Paroxetine Hydrochloride Hydrate
Pemirolast Potassium
Penbutolol Sulfate
Perphenazine Maleate
Liquefied Phenol
Phenol for Disinfection
Phenolated Water
Phenolated Water for Disinfection
Phenytoin Tablets
Pilsicainide Hydrochloride Hydrate
Pilsicainide Hydrochloride Capsules
Pimozide
Pioglitazone Hydrochloride
Pioglitazone Hydrochloride Tablets
Piperazine Adipate
Piperazine Phosphate Hydrate
Pirenzepine Hydrochloride Hydrate
Pitavastatin Calcium Hydrate
Polymixin B Sulfate
Polysorbate 80
Potassium Guaiacolsulfonate
Povidone
Pranlukast Hydrate
Prasterone Sodium Sulfate Hydrate
Pravastatin Sodium
Prazosin Hydrochloride
Prednisolone Sodium Phosphate
Probucol
Procaterol Hydrochloride Hydrate
Prochlorperazine Maleate
Progesterone Injection
L-Proline
Propafenone Hydrochloride
Propafenone Hydrochloride Tablets
Propiverine Hydrochloride
Propylthiouracil Tablets
Protirelin Tartrate Hydrate
Pyrantel Pamoate
Pyridoxine Hydrochloride
Quetiapine Fumarate
Quetiapine Fumarate Tablets
Quinapril Hydrochloride
Quinidine Sulfate Hydrate
Quinine Ethyl Carbonate
Quinine Hydrochloride Hydrate
Quinine Sulfate Hydrate
Rabeprazole Sodium
Rebamipide
Rebamipide Tablets
Risperidone
Roxatidine Acetate Hydrochloride Extended-release Capsules
Roxithromycin
Saccharin
Sarpogrelate Hydrochloride
Sarpogrelate Hydrochloride Tablets
Scopolamine Hydrobromide Hydrate
Sevoflurane
Simvastatin
Sivelestat Sodium Hydrate
Sodium Aurothiomalate
Sodium Chloride
Purified Sodium Hyaluronate
Dibasic Sodium Phosphate Hydrate
Sodium Risedronate Hydrate
Sodium Risedronate Tablets
Sodium Starch Glycolate
Sodium Valproate Tablets
Spectinomycin Hydrochloride Hydrate
Spironolactone Tablets
Rice Starch
Wheat Starch
Stearic Acid
Sulindac
Sulpiride Capsules
Sulpiride Tablets
Sultamicillin Tosilate Hydrate
Suxamethonium Chloride Hydrate
Tacalcitol Hydrate
Tacrolimus Hydrate
Taltirelin Hydrate
Tamoxifen Citrate
Tazobactam
Teceleukin (Genetical Recombination)
Teceleukin for Injection (Genetical Recombination)
Telmisartan
Temocapril Hydrochloride
Teprenone
Terbinafine Hydrochloride
Thiamine Chloride Hydrochloride
Thioridazine Hydrochloride
Tiapride Hydrochloride
Tiapride Hydrochloride Tablets
Ticlopidine Hydrochloride
Timepidium Bromide Hydrate
Tipepidine Hibenzate
Todralazine Hydrochloride Hydrate
Tosufloxacin Tosilate Hydrate
Tosufloxacin Tosilate Tablets
Tranilast
Tranilast Capsules
Trimetoquinol Hydrochloride Hydrate
Troxipide
Troxipide Tablets
Tulobuterol Hydrochloride
Ursodeoxycholic Acid Tablets
Valsartan

Valsartan Tablets
Wine
Zaltoprofen Tablets
Zolpidem Tartrate
Acacia
Powdered Acacia
Alisma Tuber
Amomum Seed
Powdered Amomum Seed
Anemarrhena Rhizome
Aralia Rhizome
Artemisia Capillaris Flower
Asiasarum Root
Asparagus Root
Astragalus Root
Bakumondoto Extract
Belladonna Root
Belladonna Extract
Belladonna Total Alkaloids
Burdock Fruit
Capsicum
Powdered Capsicum
Chotosan Extract
Cimicifuga Rhizome
Cinnamon Bark
Powdered Cinnamon Bark
Clematis Root
Coptis Rhizome
Powdered Coptis Rhizome
Corydalis Tuber
Powdered Corydalis Tuber
Daiokanzoto Extract
Daisaikoto Extract
Dioscorea Rhizome
Powdered Dioscorea Rhizome
Fennel
Powdered Fennel
Fennel Oil
Forsythia Fruit
Fritillaria Bulb
Gastrodia Tuber
Ginseng
Powdered Ginseng
Glycyrrhiza
Powdered Glycyrrhiza
Glycyrrhiza Extract
Crude Glycyrrhiza Extract
Goshajinkigan Extract
Hachimijiogan Extract
Hangekobokuto Extract
Hangeshashinto Extract
Hochuekkito Extract
Ipecac
Powdered Ipecac
Ipecac Syrup
Japanese Angelica Root
Powdered Japanese Angelica Root
Powdered Japanese Gentian
Japanese Valerian
Powdered Japanese Valerian
Japanese Zanthoxylum Peel
Powdered Japanese Zanthoxylum Peel
Juzentaihoto Extract
Kakkonto Extract
Kakkontokasenkyushin'i Extract
Kamishoyosan Extract
Keishibukuryogan Extract
Lilium Bulb
Lithospermum Root
Loquat Leaf
Lycium Fruit
Magnolia Flower
Maoto Extract
Mentha Herb
Mukoi-Daikenchuto Extract
Notopterygium
Nux Vomica
Nux Vomica Extract
Nux Vomica Extract Powder
Nux Vomica Tincture
Ophiopogon Root
Orengedokuto Extract
Otsujito Extract
Peach Kernel
Powdered Peach Kernel
Peony Root
Powdered Peony Root
Peucedanum Root
Phellodendron Bark
Powdered Phellodendron Bark
Compound Phellodendron Powder for Cataplasm
Phellodendron, Albumin Tannate and Bismuth Subnitrate Powder
Plantago Seed
Prepared Glycyrrhiza
Processed Aconite Root
Powdered Processed Aconite Root
Processed Ginger
Pueraria Root
Red Ginseng
Rhubarb
Powdered Rhubarb
Rikkunshito Extract
Ryokeijutsukanto Extract
Saibokuto Extract
Saikokeishito Extract
Saireito Extract
Saussurea Root
Scopolia Rhizome
Scopolia Extract
Scopolia Extract Powder
Scopolia Extract and Ethyl Aminobenzoate Powder

Scutellaria Root
Powdered Scutellaria Root
Senna Leaf
Powdered Senna Leaf
Shakuyakukanzoto Extract
Shimbuto Extract
Shosaikoto Extract
Shoseiryuto Extract
Sweet Hydrangea Leaf
Swertia Herb
Powdered Swertia Herb
Swertia and Sodium Bicarbonate Powder
Tokishakuyakusan Extract
Tribulus Fruit

The following revisions were made in the above monographs:

(1) The item ''Uniformity of dosage units'' in the monographs (chemical drugs, etc.) on preparations such as tablets and capsules for which the test for Mass Variation is applicable was revised according to the General Test ''Uniformity of Dosage Units''.
(2) A part of the commonly used names were deleted from the monographs (chemical drugs, etc.).
(3) The specification of residual solvent ''unless otherwise specified'' or ''Being specified separately when the drug is granted approval based on the Law'' in the Purity tests was deleted according to the Paragraph 34 of General Notices.
(4) Other descriptions were improved.

19. The following monographs were deleted:
Chlorpheniramine and Calcium Powder
Estradiol Benzoate Injection
Griseofulvin
Griseofulvin Tablets
Iodamide
Meglumine Sodium Iodamide Injection
Serum Gonadotrophin
Serum Gonadotrophin for Injection
Siccanin
Vitamin A Oil Capsules

20. The following articles were newly added to Ultraviolet-visible Reference Spectra:
Ampiroxicam
Cilnidipine
Citicoline
Eplerenone
Irbesartan
Lansoprazole
Medroxyprogesterone Acetate
Mitiglinide Calcium Hydrate
Montelukast Sodium
Ribavirin
Silodosin
Tulobuterol
Valaciclovir Hydrochloride
Voriconazole

21. The following articles in Ultraviolet-visible Reference Spectra were deleted:
Griseofulvin
Siccanin

22. The following articles were newly added to Infrared Reference Spectra:
Ampiroxicam
Cefroxadine Hydrate
Cilnidipine
Ciprofloxacin
Ciprofloxacin Hydrochloride Hydrate
Citicoline
Diflorasone Diacetate
Eplerenone
Hydroxypropylcellulose
Irbesartan
Lansoprazole
Medroxyprogesterone Acetate
Miglitol
Mitiglinide Calcium Hydrate
Montelukast Sodium
Ribavirin
Silodosin
Trientine Hydrochloride
Tulobuterol
Valaciclovir Hydrochloride
Voriconazole

23. The following articles in Infrared Reference Spectra were revised:
Benserazide Hydrochloride
Thiamine Chloride Hydrochloride

24. The following articles in Infrared Reference Spectra were deleted:
Griseofulvin
Iodamide
Siccanin

Those who were engaged in the preparation of JP 17 are as follows:
Fumiaki Akahori
Teruo Amagasa
Mitsuo Aoki
Nobuo Aoyagi**
Hiroyuki Arai
Keiko Arimoto
Yuichi Arimoto
Naoki Aruga
Naoki Asai
Hiroshi Asama
Toshiki Asano
Kazuhide Ashizawa
Shinichiro Aso
Yukio Aso

Toshio Azuma
Takashi Bamba
Yosuke Demizu
Makoto Emura
Hiroyuki Fuchino
Makiko Fujii
Yuzo Fujimoto
Kiyoshi Fukuhara
Yukihiro Goda
Shingo Goto
Takashi Goto
Tamami Goto
Noriaki Habasaki
Yuji Haishima
Hideki Hakamata
Takashi Hakamatsuka
Kentaro Hanada
Ruri Hanajiri
Toshikazu Harada
Akira Harazono
Mitsuru Hashida*
Noritaka Hashii
Rika Hatano
Yoshiro Hattori
Takao Hayakawa
Ai Hayashi
Masahiro Hayashi
Yoshinori Hayashi
Taro Higano
Kenji Higuchi
Kenichi Hirai
Yuuki Hirata
Yukio Hiyama
Masatoshi Hori
Naoki Hosono
Kenji Hosoya
Masashi Hyuga
Koji Ichinose
Nobukazu Igoshi
Yoshiaki Ikarashi
Shingo Ikedo
Kazuhiko Ikegami
Koichi Inubushi
Akiko Ishii
Tsuneo Ishizuka
Shigeru Itai
Michiho Ito
Ryoichi Ito
Yuji Ito
Chizuko Itoh
Takashi Itoh
Kenichi Izutsu
Kazuaki Kakehi
Takemine Kanai
Makoto Kanebako
Norio Karibe
Hirohito Katayama
Haru Kato
Kumiko Kato
Yoshiaki Kato
Noriko Katori
Nobuo Kawahara
Urao Kawakami
Tomomi Kawamata
Toru Kawanishi**
Yoshihiko Kawarasaki
Nana Kawasaki
Keiji Kijima
Yutaka Kikuchi
Yuuichi Kikuchi
Kimio Kiryu
Yasuhiro Kishimoto
Mitsukazu Kitada
Akihito Kitajima
Atsushi Kittaka
Fumiyuki Kiuchi
Junko Kizu
Takashi Kobayashi
Rika Kochi
Yukari Koga
Masayoshi Kohase
Tatsuo Koide
Hajime Kojima
Shigeo Kojima
Hiroyasu Kokubo
Katsuko Komatsu
Toshifumi Konda
Kenji Kondo
Seizo Kondo
Atsutaka Kubosaki
Kenichi Kumasaka
Masaaki Kurihara
Fumiyo Kusu
Hideki Kusunoki
Midori Makita
Toshinobu Makiura
Takuro Maruyama
Makoto Matsumoto
Hajime Matsumura
Eiichi Mikami
Takao Mitsuhashi
Naoki Miyata
Tamaki Miyazaki
Mitsuo Mori
Nobuaki Moriguchi
Seiki Morimoto
Takashi Morimoto
Takahito Morisaki
Katsuhiro Morisawa
Osamu Morita
Takashi Morita
Toshimi Murai

Masashi Muroi
Takashi Nagashima
Takahiro Naito
Shinsaku Nakagawa
Yukari Nakagawa
Toru Nakai
Emi Nakajima
Hirohide Nakamura
Hiroshi Nakamura
Tatsuya Nakano
Tatsumi Nakashima
Mitsuo Nanaura
Masao Nasu
Shingo Niimi
Yasuhiko Nishi
Yutaka Nishihara
Atsushi Nukanobu
Sumiaki Oba
Yoshiaki Ogasawara
Yasumitsu Ogura
Yuko Ohfuku
Yasutaka Ohgami
Ryozo Oishi
Akira Okonogi
Tsuneo Okubo
Akihiro Okuda
Haruhiro Okuda
Takamasa Okugawa
Hiroshi Onodera
Yuko Osumi
Masami Otsuka
Tadashi Ouchi
Yukio Saito
Yoshikazu Sakagami
Eiji Sakai
Tomoaki Sakamoto
Shingou Sakurai
Tomofumi Santa
Hiroshi Sasaki
Kunio Sasaki
Satoshi Sasaki
Tomoko Sasaki
Tsuguo Sasaki
Yuko Sasaki
Kazumichi Sasaoki
Kyoko Sato
Fujio Sekigawa
Michiko Sekiguchi
Setsuko Sekita
Hiroko Shibata
Keigo Shibayama
Shizuka Shida
Yasuo Shimada
Masaro Shimizu
Kozo Shimoda
Katsuaki Shinohara
Osamu Shirokizawa
Osamu Shirota
Takuji Shoda
Junya Souma
Keiichi Sudo
Chishio Sugimoto
Naoki Sugimoto
Daisuke Sugiura
Mikio Suzuki
Shigeo Suzuki
Sumiko Suzuki
Tomoyuki Suzuki
Katsumi Tachibana
Minoru Tada
Shinichi Tadaki
Nobuo Taguchi
Wataru Takada
Yoshikazu Takahashi
Kunihiro Takai
Akihito Takano
Masaki Takao
Kikuo Takatera
Osami Takeda
Tadahiro Takeda
Tomoko Takeda
Hirohumi Takeuchi
Hisashi Takeuchi
Toyoshige Tanabe
Masakazu Tanaka
Setsuko Tanaka
Tomohide Tanaka
Tomoyuki Tanaka
Toshihiro Tanaka
Kenichi Tanamoto
Tsuyoshi Tanimoto
Susumu Terabayashi
Katsuhide Terada
Reiko Teraoka
Hiroshi Tokunaga
Shogo Tokuoka
Kiyoshi Tomioka
Hiroyuki Tomitsuka
Toshiaki Tomitsuka
Taichi Toyoda
Shigeki Tsuda
Hideya Tsuge
Hiroyuki Tsujimoto
Eriko Uchida
Yoshimasa Uehara
Atsuhiro Uetake
Yoshio Wada
Eiji Watanabe
Hitoshi Watanabe
Takehiko Yajima
Toshiyasu Yamada
Teruhide Yamaguchi

Tetsuji Yamaguchi
Hiroki Yamaji
Kohji Yamakage
Keiji Yamamoto
Tosuke Yamamoto
Chikamasa Yamashita
Hitoshi Yamauchi
Takeshi Yamazaki
Masato Yasuhara
Shiho Yasuo
Hikaru Yoden
Chikako Yomota
Etsuo Yonemochi
Hiroyuki Yoshida
Kumi Yoshida
Hiroki Yotsuhashi

*: Chairman, the Committee on JP
**: Acting Chairman, the Committee on JP

The Japanese Pharmacopoeia

SEVENTEENTH EDITION

GENERAL NOTICES

1. The official name of this pharmacopoeia is 第十七改正日本薬局方, and may be abbreviated as 日局十七, 日局17, JP XVII or JP 17.

2. The English name of this pharmacopoeia is The Japanese Pharmacopoeia, Seventeenth Edition.

3. Among drugs, the Japanese Pharmacopoeia Drugs (the JP Drugs) are those specified in the monographs. The title names and the commonly used names adopted in the monograph should be used as official names. In the drug monograph, in addition to English name, chemical name or Latin name can be mentioned in the title, as appropriate.

4. Crude Drugs and their related products are placed together in ''Crude Drugs and Related Drugs'' in the posterior part of the Official Monographs. These include: Extracts, Powders, Tinctures, Syrups, Spirits, Fluidextracts or Suppositories containing Crude Drugs as the active ingredient, and combination preparations containing Crude Drugs as the principal active ingredient.

5. The JP Drugs are to be tested according to the provisions given in the pertinent monographs, General Notices, General Rules for Crude Drugs, General Rules for Preparations, and General Tests for their conformity to the Japanese Pharmacopoeia. However, the headings of ''Description'' and in addition ''Containers and storage'' in the monographs on preparations are given for information, and should not be taken as indicating standards for conformity. Nevertheless, Containers under ''Containers and storage'' in the monograph on preparations containing crude drugs as main active ingredients are the standards for conformity.

6. In principle, unless otherwise specified, animals used for preparing the JP Drugs or their source materials must be healthy.

7. In this English version, the JP Drugs described in the monographs begin with a capital letter.

8. The molecular formulas or constitution formulas in parentheses ( ) after the name of drugs or chemicals designate chemically pure substances. Atomic masses adopted in the Japanese Pharmacopoeia conform to the table of ''Standard Atomic Weights 2010''. Molecular masses are indicated to two decimal places rounded from three decimals.

9. The following abbreviations are used for the principal units.

meter	m
centimeter	cm
millimeter	mm
micrometer	μm
nanometer	nm
kilogram	kg
gram	g
milligram	mg
microgram	μg
nanogram	ng
picogram	pg
Celsius degree	°C
mole	mol
millimole	mmol
square centimeter	cm^2
liter	L
milliliter	mL
microliter	μL
megahertz	MHz
per centimeter	cm^{-1}
newton	N
kilopascal	kPa
pascal	Pa
pascal second	Pa·s
millipascal second	mPa·s
square millimeter per second	mm^2/s
lux	lx
mole per liter	mol/L
millimole per liter	mmol/L
mass per cent	%
mass parts per million	ppm
mass parts per billion	ppb
volume per cent	vol%
volume parts per million	vol ppm
mass per volume per cent	w/v%
microsimens per centimeter	$\mu S \cdot cm^{-1}$
endotoxin unit	EU
colony forming unit	CFU

Note: ''ppm'' used in the Nuclear Magnetic Resonance Spectroscopy indicates the chemical shift, and ''w/v%'' is used in the formula or composition of preparations.

10. The unit used for expressing the potency of the JP Drugs is recognized as the quantity of drug. Usually it is expressed by a definite quantity of a definite standard substance which shows a definite biological activity, and differs according to each drug. The units are determined, in principle, by comparison with each reference standard by means of biological methods. The term ''Unit'' used for the JP articles indicates the unit defined in the Japanese Pharmacopoeia.

11. The statement ''Being specified separately.'' in the monographs means that the tests are to be specified when the drugs are granted approval based on the Low on Securing Quality, Efficacy and Safety of Products including Pharmaceuticals and Medical Devices.

12. From the point of view of quality assurance, requirements that should be noted on manufacturing processes, if appropriate in addition to the specifications, are shown in the heading ''Manufacture'' in monograph. It may contain requirements regarding control of materials, manufacturing processes and intermediates, and requirements regarding tests in process and omission of tests for the release. The fulfilment of requirements mentioned in this heading are confirmed based on the information obtained during the establishment of manufacturing method at the development stage, the control of manufacturing processes, or the tests for the release. Also even in the case of absence of the heading ''Manufacture'' in monograph, it is important to note appropriate controls of materials, manufacturing processes and intermediates in individual drugs.

13. When an assurance that a product is of the JP Drug quality is obtained consistently from data derived from the manufacturing process validation studies, and from the records of appropriate manufacturing process control and of the test results of the quality control, some of the test items in the monograph being performed for the release of a product may be omitted as occasion demands.

14. The test methods specified in the Japanese Pharmacopoeia can be replaced by alternative methods which give better accuracy and precision. However, where a difference in test results is suspected, only the result obtained by the procedure given in the Pharmacopoeia is effective for the final judgment.

15. The details of the biological test methods may be changed insofar as they do not affect the essential qualities of the test.

16. The temperature for the tests or storage is described, in principle, in specific figures. However, the following expressions may be used instead.

Standard temperature, ordinary temperature, room temperature, and lukewarm are defined as 20°C, 15 – 25°C, 1 – 30°C, and 30 – 40°C, respectively. A cold place, unless otherwise specified, shall be a place having a temperature of 1 – 15°C.

The temperature of cold water, lukewarm water, warm water, and hot water are defined as not exceeding 10°C, 30 – 40°C, 60 – 70°C, and about 100°C, respectively.

The term ''heated solvent'' or ''hot solvent'' means a solvent heated almost to the boiling point of the solvent, and the term ''warmed solvent'' or ''warm solvent'' usually means a solvent heated to a temperature between 60°C and 70°C. The term ''heat on or in a water bath'' indicates, unless otherwise specified, heating with a boiling water bath or a steam bath at about 100°C.

Cold extraction and warm extraction are usually performed at temperatures of 15 – 25°C and 35 – 45°C, respectively.

17. To measure the number of drops, a dropping device which delivers 20 drops of water weighing 0.90 – 1.10 g at 20°C shall be used.

18. The term ''in vacuum'' indicates, unless otherwise specified, a pressure not exceeding 2.0 kPa.

19. The acidity or alkalinity of a solution, unless otherwise specified, is determined by blue or red litmus papers. To indicate these properties more precisely, pH values are used.

20. The terms in Table 1 are used to express the degree of cutting of Crude Drugs or fineness of powder Drugs.

Table 1

Sieve No.	4	6.5	8.6	18	50	100	200
Nominal Designation of sieve	4750 μm	2800 μm	2000 μm	850 μm	300 μm	150 μm	75 μm
Names of the drugs which pass through the respective sieves	Coarse cutting	Moderately fine cutting	Fine cutting	Coarse powder	Moderately fine powder	Fine powder	Very fine powder

21. The water to be used in the tests of drugs shall be the water suitable for performing the relevant test, such as the water not containing any substance that would interfere with the test.

22. As for wording ''solution of a solute'', where the name of the solvent is not stated, the term ''solution'' indicates a solution in water.

23. For solution an expression such as ''(1 in 3)'', ''(1 in 10)'', or ''(1 in 100)'' means that 1 g of a solid is dissolved in, or 1 mL of a liquid is diluted with the solvent to make the total volume of 3 mL, 10 mL or 100 mL, respectively. For the liquid mixture an expression such as ''(10:1)'' or ''(5:3:1)'' means that the respective numbers of parts, by volume, of the designated liquids are to be mixed.

24. The term ''weigh accurately'' means to weigh down to the degree of 0.1 mg, 10 μg, 1 μg or 0.1 μg by taking into account the purpose of the test and using a relevant weighing device. The term ''weigh exactly'' means to weigh to the given decimal places.

25. A value of ''n'' figures in a test of the JP Drugs shall be obtained by rounding off a value of ''n + 1'' figures.

26. Unless otherwise specified, all tests of the JP Drugs shall be performed at the ordinary temperature and observations of the results shall follow immedi-

ately after the operations. However, the judgment for a test which is affected by temperature should be based on the conditions at the standard temperature.

27. The terms ''immediately''/''at once'' used in the test of the JP Drugs mean that the procedure is to be performed within 30 seconds after the preceding procedure.

28. In the section under the heading Description, the term ''white'' is used to indicate white or practically white, and ''colorless'' is colorless or practically colorless. Unless otherwise specified, the test of color is carried out by placing 1 g of a solid drug on a sheet of white paper or in a watch glass placed on white paper. A liquid drug is put into a colorless test tube of 15-mm internal diameter and is observed in front of a white background through a layer of 30 mm. For the test of clarity of liquid drugs the same procedure is applied with either a black or white background. For the observation of fluorescence of a liquid drug, only a black background shall be used.

29. In the section under the heading Description, the term ''odorless'' is used to indicate odorless or practically odorless. Unless otherwise specified, the test of odor shall be carried out by placing 1 g of a solid drug or 1 mL of a liquid drug in a beaker.

30. In the section under the heading Description, solubilities are expressed by the terms in Table 2. Unless otherwise specified, solubility means the degree of dissolution of the JP Drugs, previously powdered in the case of a solid drug, within 30 minutes in a solvent at 20 ± 5°C, by vigorous shaking for 30 seconds each time at 5-minute intervals.

Table 2

Descriptive term	Volume of solvent required for dissolving 1 g or 1 mL of solute
Very soluble	Less than 1 mL
Freely soluble	From 1 mL to less than 10 mL
Soluble	From 10 mL to less than 30 mL
Sparingly soluble	From 30 mL to less than 100 mL
Slightly soluble	From 100 mL to less than 1000 mL
Very slightly soluble	From 1000 mL to less than 10000 mL
Practically insoluble, or insoluble	10000 mL and over

31. In the test of a drug, the term ''dissolve'' or ''miscible'' indicates that it dissolves in, or mixes in arbitrary proportion with the solvent to form a clear solution or mixture. Insoluble materials other than the drug including fibers should not be detected or practically invisible, if any.

32. Identification is the test to identify the active ingredient(s) of the drug based upon its specific property.

33. Purity is the test to detect impurities/contaminants in drugs, and it, as well as other requirements in each monograph, specifies the purity of the drug usually by limiting the kind/nature and quantity of the impurities/contaminants. The impurities/contaminants subject to the purity test are those supposed to generate/contaminate during the manufacturing process or storage, including hazardous agents such as heavy metals, arsenic, etc. If any foreign substances are used or supposed to be added, it is necessary to perform tests to detect or limit the presence of such substances.

34. In principle, unless specified in the monograph, the JP Drugs are controlled appropriately according to the direction under Residual Solvents of the general tests.

35. Concerning harmful substances reported as intentionally contaminated to drugs, the control requirement for the presence or absence of contamination is described in the heading ''Potential adulteration'' in the monograph, as necessary. These substances are controlled by tests on materials, manufacturing processes, intermediates, or final products. The necessity and frequency of the tests are specified separately on individual drugs depending on the control strategy established as part of quality risk management.

36. The term ''constant mass'' in drying or ignition, unless otherwise specified, means that the mass difference after an additional 1 hour of drying or ignition is not more than 0.10% of the preceding mass of the dried substance or ignited residue. For Crude Drugs, the difference is not more than 0.25%. However, when the difference does not exceed 0.5 mg in a chemical balance, 50 μg in a semi-microbalance, or 5 μg in a microbalance, the constant mass has been attained.

37. Assay is the test to determine the composition, the content of the active ingredients, and the potency unit of medicine by physical, chemical or biological procedures.

38. In stating the appropriate quantities to be taken for assay, the use of the word ''about'' indicates a quantity within 10% of the specified mass. The word ''dry'' in respect of the sample indicates drying under the same conditions, as described in Loss on drying in the monograph.

39. For the content of an ingredient determined by Assay in the monographs, if it is expressed simply as ''not less than a certain percentage'' without indicating its upper limit, 101.0% is understood as the upper limit.

40. Sterility means a condition when no target microorganism is detected by the specified method. Sterilization means a process whereby killing or removal of all living microorganisms in an object to be sterilized is accomplished. Aseptic technique is con-

trolled technique to maintain the aseptic condition.

41. The container is the device which holds the JP Drugs. The stopper or cap, etc., is considered as part of the container. The containers have no physical and chemical reactivity affecting the specified description and quality of the contents.

42. A well-closed container protects the contents from extraneous solids and from loss of the drug under ordinary or customary conditions of handling, shipment, and storage.

Where a well-closed container is specified, it may be replaced by a tight container.

43. A tight container protects the contents from extraneous solids or liquids, from loss of the contents, and from efflorescence, deliquescence, or evaporation under ordinary or customary conditions of handling, shipment, and storage.

Where a tight container is specified, it may be replaced by a hermetic container.

44. A hermetic container is impervious to air or any other gas under ordinary or customary conditions of handling, shipment, and storage.

45. The term ''light-resistant'' means that it can prevent transmittance of light affecting in the specified properties and quality of the contents and protect the contained medicament from the light under ordinary or customary conditions of handling, shipment, and storage.

46. For the JP Drugs, the contents or potency in terms of units of the active ingredient(s), or the specified expiration date in the monographs have to be shown on the immediate container or wrapping of them.

47. The origin, numerical value or physical properties of the JP Drugs, being stipulated by the special labeling requirements in the monographs, have to be shown on the immediate container or wrapping of them.

48. The harmonized General Tests and Monographs among the Japanese Pharmacopoeia, the European Pharmacopoeia and the United States Pharmacopeia are preceded by the statement as such.

The parts of the text, being not harmonized, are surrounded by the symbols (◆ ◆ or ◇ ◇).

—Abbreviations—

CS: Colorimetric Stock Solution
RS: Reference Standard
TS: Test Solution
VS: Refer to a solution listed in Standard Solutions for Volumetric Analysis <*9.21*>.

GENERAL RULES FOR CRUDE DRUGS

1. Crude drugs in the monographs include medicinal parts obtained from plants or animals, cell inclusions and secretes separated from the origins, their extracts, and minerals. General Rules for Crude Drugs and Crude Drugs Test are applicable to the following:

Acacia, Achyranthes Root, Agar, Akebia Stem, Alisma Tuber, Aloe, Alpinia Officinarum Rhizome, Aluminum Silicate Hydrate with Silicon Dioxide, Amomum Seed, Anemarrhena Rhizome, Angelica Dahurica Root, Apricot Kernel, Aralia Rhizome, Areca, Artemisia Capillaris Flower, Artemisia Leaf, Asiasarum Root, Asparagus Root, Astragalus Root, Atractylodes Lancea Rhizome, Atractylodes Rhizome, Bear Bile, Bearberry Leaf, Belladonna Root, Benincasa Seed, Benzoin, Bitter Cardamon, Bitter Orange Peel, Brown Rice, Bupleurum Root, Burdock Fruit, Calumba, Capsicum, Cardamon, Cassia Seed, Catalpa Fruit, Cherry Bark, Chrysanthemum Flower, Cimicifuga Rhizome, Cinnamon Bark, Cistanche Herb, Citrus Unshiu Peel, Clematis Root, Clove, Cnidium Monnieri Fruit, Cnidium Rhizome, Codonopsis Root, Coix Seed, Condurango, Coptis Rhizome, Cornus Fruit, Corydalis Tuber, Crataegus Fruit, Cyperus Rhizome, Digenea, Dioscorea Rhizome, Dolichos Seed, Eleutherococcus Senticosus Rhizome, Ephedra Herb, Epimedium Herb, Eucommia Bark, Euodia Fruit, Fennel, Forsythia Fruit, Fritillaria Bulb, Gambir, Gardenia Fruit, Gastrodia Tuber, Gentian, Geranium Herb, Ginger, Ginseng, Glehnia Root and Rhizome, Glycyrrhiza, Gypsum, Hedysarum Root, Hemp Fruit, Honey, Houttuynia Herb, Immature Orange, Imperata Rhizome, Ipecac, Japanese Angelica Root, Japanese Gentian, Japanese Valerian, Japanese Zanthoxylum Peal, Jujube, Jujube Seed, Koi, Leonurus Herb, Lilium Bulb, Lindera Root, Lithospermum Root, Longan Aril, Longgu, Lonicera Leaf and Stem, Loquat Leaf, Lycium Bark, Lycium Fruit, Magnolia Bark, Magnolia Flower, Mallotus Bark, Malt, Mentha Herb, Moutan Bark, Mulberry Bark, Nelumbo Seed, Notopterygium, Nuphar Rhizome, Nutmeg, Nux Vomica, Ophiopogon Root, Oriental Bezoar, Oyster Shell, Panax Japonicus Rhizome, Peach Kernel, Peony Root, Perilla Herb, Peucedanum Root, Pharbitis Seed, Phellodendron Bark, Picrasma Wood, Pinellia Tuber, Plantago Herb, Plantago Seed, Platycodon Root, Pogostemon Herb, Polygala Root, Polygonatum Rhizome, Polygonum Root, Polyporus Sclerotium, Poria Sclerotium, Powdered Acacia, Powdered Agar, Powdered Alisma Tuber, Powdered Aloe, Powdered Amomum Seed, Powdered Atractylodes Lancea Rhizome, Powdered Atractylodes Rhizome, Powdered Calumba, Powdered Capsicum, Powdered Cinnamon Bark, Powdered Clove, Powdered Cnidium Rhizome, Powdered Coix Seed, Powdered Coptis Rhizome, Powdered Corydalis Tuber, Powdered Cyperus Rhizome, Powdered Dioscorea Rhizome, Powdered Fennel, Powdered Gambir, Powdered Gardenia Fruit, Powdered Gentian, Powdered Geranium Herb, Powdered Ginger, Powdered Ginseng, Powdered Glycyrrhiza, Powdered Ipecac, Powdered Japanese Angelica Root, Powdered Japanese Gentian, Powdered Japanese Valerian, Powdered Longgu, Powdered Magnolia Bark, Powdered Moutan Bark, Powdered Oyster Shell, Powdered Panax Japonicus Rhizome, Powdered Peach Kernel, Powdered Peony Root, Powdered Phellodendron Bark, Powdered Picrasma Wood, Powdered Platycodon Root, Powdered Polygala Root, Powdered Polyporus Sclerotium, Powdered Poria Sclerotium, Powdered Processed Aconite Root, Powdered Rhubarb, Powdered Rose Fruit, Powdered Scutellaria Root, Powdered Senega, Powdered Senna Leaf, Powdered Smilax Rhizome, Powdered Sophora Root, Powdered Sweet Hydrangea Leaf, Powdered Swertia Herb, Powdered Tragacanth, Powdered Turmeric, Powdered Zanthoxylum Fruit, Prepared Glycyrrhiza, Processed Aconite Root, Processed Ginger, Prunella Spike, Pueraria Root, Quercus Bark, Red Ginseng, Rehmannia Root, Rhubarb, Rose Fruit, Rosin, Royal Jelly, Safflower, Saffron, Salvia Miltiorrhiza Root, Saposhnikovia Root and Rhizome, Sappan Wood, Saussurea Root, Schisandra Fruit, Schizonepeta Spike, Scopolia Rhizome, Scutellaria Root, Senega, Senna Leaf, Sesame, Sinomenium Stem and Rhizome, Smilax Rhizome, Sophora Root, Sweet Hydrangea Leaf, Swertia Herb, Toad Cake, Tragacanth, Tribulus Fruit, Trichosanthes Root, Turmeric, Uncaria Hook, Zedoary.

2. Crude drugs are usually used in the forms of whole crude drugs, cut crude drugs or powdered crude drugs.

Whole crude drugs are the medicinal parts or their ingredients prepared by drying and/or simple processes, as specified in the monographs.

Cut crude drugs are small pieces or small blocks prepared by cutting or crushing of the whole crude drugs,

and also coarse, moderately fine or fine cutting of the crude drugs in whole, and, unless otherwise specified, are required to conform to the specifications of the whole crude drugs used as original materials.

Powdered crude drugs are coarse, moderately fine, fine or very fine powder prepared from the whole crude drugs or the cut crude drugs; usually powdered crude drugs as fine powder are specified in the monographs.

3. Unless otherwise specified, crude drugs are used in dried form. The drying is usually carried out at a temperature not exceeding 60°C.

4. The origin of crude drugs is to serve as the acceptance criteria. Such statements as 'other species of the same genus' and 'allied plants' or 'allied animals' appearing in the origin of crude drugs usually indicate plants or animals which may be used as materials for crude drugs containing the same effective constituents.

5. Description in each monograph for crude drugs covers the crude drug derived from its typical original plant or animal and includes statements of characteristic properties of the crude drug, which are all to serve as the evaluation criteria as well as the aspects obtained by microscopic observatoin. As for the color, odor and solubility, apply correspondingly to the prescription of the General Notices, except the odor which is to serve as the acceptance criteria. The taste is to serve as the acceptance criteria.

6. Powdered crude drugs, otherwise specified, may be mixed with diluents so as to attain proper content and potency.

7. Powdered crude drugs do not contain fragments of tissues, cells, cell inclusions or other foreign matter alien to the original crude drugs or cut crude drugs.

8. Crude drugs are as free as possible from contaminants and other impurities due to molds, insects and other animals and from other foreign matters, and are required to be kept in a clean and hygienic state.

9. Crude drugs are preserved under protection from moisture and insect damage, unless otherwise specified. In order to avoid insect damage, suitable fumigants may be used to preserve crude drugs, provided that the fumigants are so readily volatilized as to be harmless at the usual dosage of the crude drugs, and such fumigants that may affect the therapeutic efficacy of the crude drugs or interfere with the testing are precluded.

10. Crude drugs are preserved in well-closed containers unless otherwise specified.

GENERAL RULES FOR PREPARATIONS

[1] General Notices for Preparations

(1) General Notices for Preparations present general rules for pharmaceutical dosage forms.

(2) In [3] Monographs for Preparations, dosage forms are classified mainly by administration routes and application sites, and furthermore are subdivided according to their forms, functions and characteristics.

Those preparations containing mainly crude drugs as active raw materials are described under [4] Monographs for Preparations Related to Crude Drugs.

(3) In Monographs for Preparations and Monographs for Preparations Related to Crude Drugs, dosage forms, which are generally or widely used, are described. However, any other appropriate dosage forms may be used where appropriate. For example, a dosage form suitable for a particular application may be designated by combining an administration route and a name of a dosage form listed in these chapters.

(4) In these monographs, preparation characteristics are specified for the dosage forms. The preparation characteristics are confirmed by appropriate tests.

(5) In the case of preparations, functions that control the release rate of active substance(s) may be added for the purpose of controlling the onset and duration of therapeutic effects and/or decreasing adverse or side effects. The preparations modified in release rate must have an appropriate function of controlled release for the intended use. The added functional modification must generally be displayed on the pack insert and on the direct container or packaging of these preparations.

(6) Pharmaceutical excipients are substances other than active substances contained in preparations, and they are used to increase the utility of the active substance(s) and preparation, to make formulation process easier, to keep the product quality, to improve the usability, and so forth. Suitable excipients may be added for these purposes. The excipients to be used, however, must be pharmacologically inactive and harmless in the administered amount and must not interfere with the therapeutic efficacy of the preparations.

(7) Purified water to be used for preparations is Purified Water or Purified Water in Containers, and water for injection is Water for Injection or Water for Injection in Containers.

Vegetable oils to be used for preparations are usually edible oils listed in the Pharmacopoeia. When starch is called for, unless otherwise specified, any kind of starch listed in the Pharmacopoeia may be used.

In addition, ethanol specified in vol% is prepared by adding Purified Water or Water for Injection to ethanol at the specified vol%.

(8) Sterile preparations are preparations verified to be aseptic. There are terminal sterilization and aseptic technique as basic manufacturing process of sterile preparations.

Terminal sterilization is a process to sterilize preparations after filling in a container. In this process microbial lethality after sterilization is quantitatively measured or evaluated, and this process is performed under the condition where the sterility assurance level of 10^{-6} or less is ensured by using suitable biological indicators.

Aseptic technique is a process for appropriate control of a risk of microbial contamination, and is a manufacturing process of preparations using a series of aseptic processes with sterile raw materials or after filtration sterilization.

This technique generally requires the presterilization of all equipments and materials used, and this process is performed under the condition to give a defined sterility assurance level in the clean areas where microbial and particulate levels are adequately maintained by using appropriate techniques.

(9) Even non-sterile preparations should be prepared with precautions to prevent contamination and growth of microorganisms, and they are applied to the test of Microbiological Examination of Non-sterile Products <*4.05*>, if necessary.

(10) The test for Content Uniformity under the Uniformity of Dosage Units and the Dissolution Test are not intended to apply to the crude drug component of preparations which are prepared using crude drugs or preparations related to crude drugs as raw materials.

(11) Unless otherwise specified, preserve preparations at room temperature. Store them in light-resistant containers or packaging, if light affects the quality of the preparation.

[2] General Notices for Packaging of Preparations

(1) General notices for packaging of preparations describe the basic items on a principle and the packaging suitability for packaging of preparations using container and wrapper.

(2) Principle of packaging of preparations

In the development phase of preparations, it is important for the packaging of preparations to fully evaluate its suitability for maintaining the specified quality of preparations over the shelf life. Based on the evaluation of the packaging suitability depending on the characteristics of the preparation, items such as specification and test methods of finished products, in-process tests and evaluation of the materials used for packaging and the like to control the quality appropriately are established. The properness of the established requirements can be verified conclusively by the stability studies of finished products.

For the change of the packaging, it is necessary to examine the items described above.

It is necessary to perform the appropriate test to confirm that the unintended changes of the packaging exert no influence to the quality of finished product.

(3) Packaging suitability

Packaging suitability includes the components of Protection of preparation, Compatibility of preparation and package, Safety of the materials used for package, and additional Performance at the time of administration.

Depending on the characteristics of preparation, the package should have functions such as of moisture-proofness, light-resistance, barrier property for gases and microorganisms, and protection against the shock that might occur at the time of transportation, and the like (Protection).

The package should be composed of the shape and material that do not cause physical and chemical interaction with the preparation (Compatibility).

It should be composed of the materials which leaching and migrating quantity of the constituents and impurities to preparations are sufficiently low from the point of view of safety (Safety).

The packaging performance shall include not merely the protection of preparations but also the improvement of patient compliance, ease of use, etc. Functions of ensuring safety of patients such as a prevention of accidental ingestion and improvement of safety of medical staffs should also be included (Performance).

The packaging suitability is examined based on the test methods listed in the General Tests and appropriate techniques depending on the dosage form and the characteristics of the preparation. Items for appropriate quality control are established based on the test methods and the like used for packaging suitability.

For designing of the packaging of injections, the packaging suitability is examined by appropriate selection from Test for Glass Containers for Injections <*7.01*>, Test Methods for Plastic Containers <*7.02*>, Test for Rubber Closure for Aqueous Infusions <*7.03*>, a container integrity test, a light stability test, the descriptions in Monographs, and the like. Items for appropriate quality control are established based on the adopted techniques for the packaging suitability.

[3] Monographs for Preparations

(1) In the Monographs for Preparations, the definitions of dosage forms, manufacturing methods, test methods, containers and packaging, and storage are described.

(2) The descriptions of the test methods in these monographs are fundamental requirements, and the manufacturing methods represent commonly used methods.

(3) Preparation in single-dose package means a preparation packaged for single-dose use.

1. Preparations for Oral Administration

(1) Immediate-release dosage forms are preparations showing a release pattern of active substance(s) that is not intentionally modified and is generally dependent on the intrinsic solubility of the active substance.

(2) Modified-release dosage forms are preparations showing a release pattern of active substance(s) that is suitably modified for the desired purpose by means of a specific formulation design and/or manufacturing method. Modified-release dosage forms include delayed-release and extended-release preparations.

(i) Delayed-release preparations

Delayed-release preparations are designed to release the bulk of the active substance(s) not in stomach but mainly in small intestine, in order to prevent degradation or decomposition of the active substance(s) in stomach or to decrease the irritation of the active substance(s) on stomach. Delayed-release preparations are generally coated with an acid-insoluble enteric film. Delayed-release preparations are included in a group of modified-release dosage forms that delay the start to release active substance(s).

(ii) Extended-release preparations

Extended-release preparations are designed to control the release rate and release period of active substance(s) and to restrict the release to appropri-

ate sites in the gastrointestinal tracts in order to decrease the dosing frequency and/or to reduce adverse or side effects. Extended-release preparations are generally prepared by using suitable agents that prolong the release of the active substance(s).

(3) Oral dosage forms such as capsules, granules and tablets can be coated with appropriate coating agents, such as sugars, sugar alcohols, or polymers, for the purpose of enabling the ingestion easy or of preventing degradation of the active substance(s).

1-1. Tablets

(1) Tablets are solid preparations having a desired shape and size, intended for oral administration. Orally Disintegrating Tablets, Chewable Tablets, Effervescent Tablets, Dispersible Tablets and Soluble Tablets are included in this category.

(2) Tablets are usually prepared by the following procedures. Delayed-release or extended-release tablets can be prepared by appropriate methods.

(i) Mix homogeneously active substance(s) and excipients such as diluents, binders and disintegrators, granulate with water or a binder solution by a suitable method, mix with a lubricant, and then compress into a desired shape and size.

(ii) Mix homogeneously active substance(s) and excipients such as diluents, binders, and disintegrators, and then directly compress with a lubricant, or compress after adding active substance(s) and a lubricant to granules previously prepared from excipients and then mixing homogeneously.

(iii) Mix homogeneously active substance(s) and excipients such as diluents and binders, moisten with a solvent, form into a certain shape and size or mold the mixed mass into a certain shape and size, and then dry by a suitable method.

(iv) Plain Tablets are usually prepared according to (i), (ii) or (iii).

(v) Film-coated Tablets can be prepared, usually, by coating Plain Tablets with thin films using suitable film coating agents such as polymers.

(vi) Sugar-coated Tablets can be prepared, usually, by coating Plain Tablets using suitable coating agents including sugars or sugar alcohols.

(vii) Multiple-layer Tablets can be prepared by compressing granules of different compositions to form layered tablets by a suitable method.

(viii) Pressure-coated Tablets can be prepared by compressing granules to cover inner core tablets with different compositions.

(3) Unless otherwise specified, Tablets meet the requirements of Uniformity of Dosage Units <*6.02*>.

(4) Unless otherwise specified, Tablets meet the requirements of Dissolution Test <*6.10*> or Disintegration Test <*6.09*>. For Effervescent tablets from which active substance(s) are dissolved before use and Soluble tablets, these tests are not required.

(5) Well-closed containers are usually used for the preparations. For preparations susceptible to degradation by moisture, a moisture-proof container or packaging may be used.

1-1-1. Orally Disintegrating Tablets/Orodispersible Tablets

(1) Orally Disintegrating Tablets are tablets which are quickly dissolved or disintegrated in the oral cavity.

(2) Orally Disintegrating Tablets shows an appropriate disintegration.

1-1-2. Chewable Tablets

(1) Chewable Tablets are tablets which are administered by chewing.

(2) Chewable Tablets must be in shape and size avoiding danger of suffocation.

1-1-3. Effervescent Tablets

(1) Effervescent Tablets are tablets which are quickly dissolved or dispersed with bubbles in water.

(2) Effervescent tablets are usually prepared using suitable acidic substances and carbonates or hydrogen carbonates.

1-1-4. Dispersible Tablets

(1) Dispersible Tablets are tablets which are administered after having been dispersed in water.

1-1-5. Soluble Tablets

(1) Soluble Tablets are tablets which are administered after having been dissolved in water.

1-2. Capsules

(1) Capsules are preparations enclosed in capsules or wrapped with capsule bases, intended for oral administration. Capsules are classified into Hard Capsules and Soft Capsules.

(2) Capsules are usually prepared by the following methods. Delayed-release or extended-release capsules can be prepared by a suitable method. Coloring agents, preservatives, etc. may be added to the capsule bases.

(i) Hard Capsules: A homogeneous mixture of active substance(s) with diluents and other suitable excipients, or granules or formed masses prepared by a suitable method, are filled into capsule shells as they are or after slight compression.

(ii) Soft Capsules: Active substance(s) and suitable excipients (including solvents) are mixed, enclosed by a suitable capsule base such as gelatin plasticized by addition of glycerin, D-sorbitol, etc. and molded in a suitable shape and size.

(3) Unless otherwise specified, Capsules meet the requirements of Uniformity of Dosage Units <*6.02*>.

(4) Unless otherwise specified, Capsules meet the requirements of Dissolution Test <*6.10*> or Disintegration Test <*6.09*>.

(5) Well-closed containers are usually used for

Capsules. For Capsules susceptible to degradation by moisture, a moisture-proof container or packaging may be used.

1-3. Granules

(1) Granules are preparations prepared by granulation, intended for oral administration. Effervescent Granules are included in this category.

(2) Granules are usually prepared by the following methods. Granules can be coated using suitable coating agents if necessary. Extended-release or delayed-release granules can also be prepared by a suitable method.

(i) To powdery active substance(s) add diluents, binders, disintegrators, or other suitable excipients, mix to homogenize, and granulate by a suitable method.

(ii) To previously granulated active substance(s) add excipients such as diluents, and mix to homogenize.

(iii) To previously granulated active substance(s) add excipients such as diluents, and granulate by a suitable method.

(3) Among Granules, the preparations may be referred to as ''Fine Granules'' if, when Particle Size Distribution Test for Preparations <*6.03*> is performed, all granules pass through a No. 18 (850 μm) sieve, and not more than 10% of which remain on a No. 30 (500 μm) sieve.

(4) Unless otherwise specified, the Granules in single-dose packages meet the requirements of Uniformity of Dosage Units <*6.02*>.

(5) Unless otherwise specified, Granules comply with Dissolution Test <*6.10*> or Disintegration Test <*6.09*>. However, this provision is not to be applied to Effervescent granules, which are dissolved before use, and Disintegration Test <*6.09*> is not required for the Granules not more than 10% of which remain on a No. 30 (500 μm) sieve when the test is performed as directed under Particle Size Distribution Test for Preparations <*6.03*>.

(6) Among Granules, the particulate preparations may be referred to as ''Powders'' if, when the Particle Size Distribution Test for Preparations <*6.03*> is performed, all granules pass through a No. 18 (850 μm) sieve, and not more than 5% remain on a No. 30 (500 μm) sieve.

(7) Well-closed containers are usually used for Granules. For the preparations susceptible to degradation by moisture, a moisture-proof container or packaging may be used.

1-3-1. Effervescent Granules

(1) Effervescent granules are granules which are quickly dissolved or dispersed with bubbles in water.

(2) Effervescent granules are usually prepared using suitable acidic substances and carbonates or hydrogen carbonates.

1-4. Powders

(1) Powders are preparations in powder form, intended for oral administration.

(2) Powders are usually prepared by homogeneously mixing active substance(s) with diluents or other suitable excipients.

(3) Unless otherwise specified, the Powders in single-dose packages meet the requirements of Uniformity of Dosage Units <*6.02*>.

(4) Unless otherwise specified, Powders meet the requirements of Dissolution Test <*6.10*>.

(5) Well-closed containers are usually used for Powders. For the preparations susceptible to degradation by moisture, a moisture-proof container or packaging may be used.

1-5. Liquids and Solutions for Oral Administration

(1) Liquids and Solutions for Oral Administration are preparations in liquid form or flowable and viscous gelatinous state, intended for oral administration. Elixirs, Suspensions, Emulsions and Lemonades are included in this category.

(2) Liquids and Solutions for Oral Administration are usually prepared by dissolving, emulsifying or suspending active substance(s) in Purified Water together with excipients, and by filtering if necessary.

(3) For Liquids and Solutions for Oral Administration which are apt to deteriorate, prepare before use.

(4) Unless otherwise specified, the preparations in single-dose packages meet the requirement of Uniformity of Dosage Units <*6.02*>.

(5) Tight containers are usually used for Liquids and Solutions for Oral Administration. For the preparations susceptible to degradation by evaporation of water, a low-moisture-permeability container or packaging may be used.

1-5-1. Elixirs

(1) Elixirs are clear, sweetened and aromatic liquid preparations, containing ethanol, intended for oral administration.

(2) Elixirs are usually prepared by dissolving solid active substance(s) or their extractives in ethanol and Purified Water, adding aromatic agents and sucrose, other sugars or sweetening agents, and clarifying by filtration or other procedure.

1-5-2. Suspensions

(1) Suspensions are liquid preparations of active substance(s) suspended finely and homogeneously in a vehicle, intended for oral administration.

(2) Suspensions are usually prepared by adding suspending agent or other suitable excipients and Purified Water or oil to solid active substance(s), and suspending homogeneously as the whole by a suitable

method.

(3) Mix homogeneously before use, if necessary.

(4) Unless otherwise specified, Suspensions meet the requirements of Dissolution Test <*6.10*>.

1-5-3. Emulsions

(1) Emulsions are liquid preparations of active substance(s) emulsified finely and homogeneously in a liquid vehicle, intended for oral administration.

(2) Emulsions are usually prepared by adding emulsifying agents and Purified Water to liquid active substance(s), and emulsifying finely and homogeneously by a suitable method.

(3) Mix homogeneously before use, where necessary.

1-5-4. Lemonades

(1) Lemonades are sweet and sour, clear liquid preparations, intended for oral administration.

1-6. Syrups

(1) Syrups are viscous liquid or solid preparations containing sugars or sweetening agents, intended for oral administration. Preparations for Syrups are included in this category.

(2) Syrups are usually prepared by dissolving, mixing, suspending or emulsifying active substance(s) in a solution of sucrose, other sugars or sweetening agents, or in Simple Syrup. Where necessary, the mixture is boiled, and filtered while hot.

(3) For Syrups which are apt to deteriorate, prepare before use.

(4) Unless otherwise specified, Syrups in single-dose packages meet the requirements of Uniformity of Dosage Units <*6.02*>.

(5) Unless otherwise specified, Syrups in which active substance(s) is suspended meet the requirements of Dissolution Test <*6.10*>.

(6) Tight containers are usually used for Syrups. For the preparations susceptible to degradation by evaporation of water, a low-moisture-permeability container or packaging may be used.

1-6-1. Preparations for Syrups

(1) Preparations for Syrups are preparations in form of granules or powders, which become syrups by adding water. They may be termed ''Dry Syrups''.

(2) Preparations for Syrup are usually prepared with sugars or sweetening agents as directed under 1-3. Granules or 1-4. Powders.

(3) Preparations for Syrups are usually to be used after having been dissolved or suspended in water.

(4) Unless otherwise specified, the Preparations for Syrups other than preparations which are to be used after having been dissolved meet the requirements of Dissolution Test <*6.10*> or Disintegration Test <*6.09*>. However, Disintegration Test <*6.09*> is not required for the Preparations, if, when the Particle Size Distribution Test for Preparations <*6.03*> is performed, not more than 10% of the total amount remains on a No. 30 (500 μm) sieve.

(5) Well-closed containers are usually used for Preparations for Syrups. For the Preparations for Syrups susceptible to degradation by moisture, a moisture-proof container or packaging may be used.

1-7. Jellies for Oral Administration

(1) Jellies for Oral Administration are non-flowable gelatinous preparations having a certain shape and size, intended for oral administration.

(2) Jellies for oral application are usually prepared by mixing active substance(s) with suitable excipients and polymer gel base, gelatinizing and forming into a certain shape and size by a suitable method.

(3) Unless otherwise specified, Jellies for Oral Administration meet the requirements of Uniformity of Dosage Units <*6.02*>.

(4) Unless otherwise specified, Jellies for Oral Administration meet the requirements of Dissolution Test <*6.10*> or show an appropriate disintegration.

(5) Tight containers are usually used for Jellies for Oral Administration. For the preparations susceptible to degradation by evaporation of water, a low-moisture-permeability container or packaging may be used.

2. Preparations for Oro-mucosal Application

2-1. Tablets for Oro-mucosal Application

(1) Tablets for Oro-mucosal Application are solid preparations having a certain form, intended for oral cavity application.

Troches/Lozenges, Sublingual Tablets, Buccal Tablets, Mucoadhesive Tablets and Medicated Chewing Gums are included in this category.

(2) Tablets for Oro-mucosal Application are prepared as directed under 1-1. Tablets.

(3) Unless otherwise specified, Tablets for Oro-mucosal Application meet the requirements of Uniformity of Dosage Units <*6.02*>.

(4) Tablets for Oro-mucosal Application have an appropriate dissolution or disintegration.

(5) Well-closed containers are usually used for Tablets for Oro-mucosal Application. For the preparations susceptible to degradation by moisture, a moisture-proof container or packaging may be used.

2-1-1. Troches/Lozenges

(1) Troches/Lozenges are tablets for oro-mucosal application, which are gradually dissolved or disintegrated in the mouth, and are intended for application locally to the oral cavity or the throat.

(2) Troches/Lozenges must be in shape and size avoiding danger of suffocation.

2-1-2. Sublingual Tablets

(1) Sublingual Tablets are tablets for oro-mucosal application, from which active substance(s) are

quickly dissolved sublingually and absorbed via the oral mucosa.

2-1-3. Buccal Tablets

(1) Buccal Tablets are tablets for oro-mucosal application, from which the active substance(s) are dissolved gradually between the cheek and teeth, and absorbed via the oral mucosa.

2-1-4. Mucoadhesive Tablets

(1) Mucoadhesive Tablets are tablets for oro-mucosal application that are applied by adhesion to the oral mucosa.

(2) Mucoadhesive Tablets are usually prepared by using hydrophilic polymers to form hydrogel.

2-1-5. Medicated Chewing Gums

(1) Medicated Chewing Gums are tablets for oro-mucosal application, releasing active substance(s) by chewing.

(2) Medicated Chewing Gums are usually prepared using suitable gum bases such as vegetable resin, thermoplastic resin and elastomer.

2-2. Liquids and Solutions for Oro-mucosal Application

(1) Liquids and Solutions for Oro-mucosal Application are preparations in liquid form or flowable and viscous gelatinous state, intended for oral cavity application.

(2) Liquids and Solutions for Oro-mucosal Application are usually prepared by mixing active substance(s) with suitable excipients and Purified Water or suitable vehicles to dissolve homogenously or to emulsify or suspend, and by filtering if necessary.

(3) For Liquids and Solutions for Oro-mucosal Application which are apt to deteriorate, prepare before use.

(4) Unless otherwise specified, the preparations in single-dose packages meet the requirement of the Uniformity of Dosage Units <*6.02*>.

(5) Tight containers are usually used for Liquids and Solutions for Oro-mucosal Application. For the preparations susceptible to degradation by evaporation of water, a low-moisture-permeability container or packaging may be used.

2-2-1. Preparations for Gargles

(1) Preparations for Gargles are liquid preparations intended to apply locally to the oral and throat cavities. Solid type preparations to be dissolved in water before use are also included in this category.

(2) Solid type preparations to be dissolved in water before use are prepared as directed under 1-1. Tablets or 1-3. Granules.

2-3. Sprays for Oro-mucosal Application

(1) Sprays for Oro-mucosal Application are preparations that are applied active substance(s) by spraying into the oral cavity in mist, powder, foam or paste forms.

(2) Sprays for Oro-mucosal Application are usually prepared by the following methods:

(i) Dissolve or suspend active substance(s) and suitable excipients in a solvent, filter, where necessary, and fill into a container together with liquefied or compressed gas.

(ii) Dissolve or suspend active substance(s) and suitable excipients in a solvent, fill into a container, and fit with a pump for spraying.

(3) Unless otherwise specified, metered-dose types among Sprays for Oro-mucosal Application have an appropriate uniformity of delivered dose.

(4) Tight containers or pressure-resistant containers are usually used for Sprays for Oro-mucosal Application.

2-4. Semi-solid Preparations for Oro-mucosal Application

(1) Semi-solid Preparations for Oro-mucosal Application are preparations in cream, gel or ointment forms, intended for application to the oral mucosa.

(2) Semi-solid Preparations for Oro-mucosal Application are usually prepared by emulsifying active substance(s) together with excipients using ''Purified Water'' and oil component such as petrolatum, or by homogenizing active substance(s) together with suitable excipients using polymer gel or oil and fats as the base.

(i) Creams for oro-mucosal application are prepared as directed under 11-5. Creams.

(ii) Gels for oro-mucosal application are prepared as directed under 11-6. Gels.

(iii) Ointments for oro-mucosal application are prepared as directed under 11-4. Ointments.

For Semi-solid Preparations for Oro-mucosal Application which are apt to deteriorate, prepare before use.

(3) Sufficient amounts of suitable preservatives to prevent the growth of microorganisms may be added for Semi-solid Preparations for Oro-mucosal Application filled in multiple-dose containers.

(4) Semi-solid Preparations for Oro-mucosal Application have a suitable viscosity to apply to the oral mucosa.

(5) Tight containers are usually used for Semi-solid Preparations for Oro-mucosal Application. For the preparations susceptible to degradation by evaporation of water, a low-moisture-permeability container or packaging may be used.

3. Preparations for Injection

3-1. Injections

(1) Injections are sterile preparations to be administered directly into the body through skin, muscle or blood vessel, usually in form of a solution, a suspen-

sion or an emulsion of active substance(s), or of a solid that contains active substance(s) to be dissolved or suspended before use.

Parenteral Infusions, Implants/Pellets and Prolonged-Release Injections are included in this category.

(2) Injections in solution, suspension or emulsion form are usually prepared by the following methods.

(i) Dissolve, suspend or emulsify active substance(s) with or without excipients in Water for Injection or an aqueous or nonaqueous vehicle homogeneously, fill into containers for injection, seal, and sterilize.

(ii) Dissolve, suspend or emulsify active substance(s) with or without excipients in Water for Injection or an aqueous or nonaqueous vehicle, and filtrate aseptically, or prepare aseptically a homogeneous liquid, fill into containers for injection, and seal.

Every care should be taken to prevent contamination with microorganisms. The overall processes of preparing injections, from the preparation of active solution to the sterilization, should be completed as rapidly as possible, taking into consideration the composition of the injection and the storage conditions. The concentration of active substance(s) expressed in % represents w/v%.

Injections that are to be dissolved or suspended before use and are designated in the name as ''for injection'' may be accompanied by a suitable vehicle to dissolve or suspend the supplied preparation (hereinafter referred to as ''vehicle attached to preparation'').

(3) Injections may be prepared as Freeze-dried Injections or Powders for Injections to prevent degradation or deactivation of the active substance(s) in solution.

(i) Freeze-dried Injections

Freeze-dried Injections are usually prepared by dissolving active substance(s) with or without excipients such as diluents in Water for Injection, sterilizing the solution by aseptic filtration, filling the filtrate directly into individual containers for injection and being freeze-dried, or dividing the filtrate in special containers, being freeze-dried and transferred into individual containers for injection.

(ii) Powders for Injections

Powders for injections are usually prepared by filtrating aseptically a solution of active substance(s), obtaining powders by crystallization from the solution or mixing additionally the powders with sterilized excipients, and filling the powders into individual containers for injections.

(4) To prevent errors in the preparation with vehicles attached or administration of injections, or bacterial or foreign matter contamination, or for the purpose of urgent use, prefilled syringes or cartridges may be prepared.

(i) Prefilled Syringes for Injections

Prefilled Syringes for injections are usually prepared by dissolving, suspending or emulsifying active substance(s) with or without excipients in a vehicle, and filling into syringes.

(ii) Cartridges for Injections

Cartridges for Injections are usually prepared by dissolving, suspending or emulsifying active substance(s) with or without excipients in a vehicle, and filling into cartridges.

The cartridges are used by fixing in an injection device for exclusive use.

(5) Vehicles used in Injections or attached to preparations must be harmless in the amounts usually administered and must not interfere with the therapeutic efficacy of the active substance(s).

The vehicles are classified into the following two groups. They should meet each requirement.

(i) Aqueous vehicles: As the vehicle of aqueous injections, Water for Injection is usually used. Isotonic Sodium Chloride Solution, Ringer's Solution, or other suitable aqueous solutions may be used instead.

Unless otherwise specified, these aqueous vehicles, other than those exclusively for intracutaneous, subcutaneous or intramuscular administration, meet the requirements of Bacterial Endotoxins Test <*4.01*>.

When the Bacterial Endotoxins Test <*4.01*> is not applicable to aqueous vehicles, the Pyrogen Test <*4.04*> may be applied instead.

(ii) Non-aqueous vehicles: Vegetable oils are usually used as vehicles for oily injections. These oils, unless otherwise specified, are clear at 10°C, the acid value is not more than 0.56, the saponification value is between 185 and 200, and the iodine value falls between 79 and 137. They meet the requirements of Mineral Oil Test <*1.05*>.

Organic vehicles miscible with water, such as ethanol, are usually used as vehicles for hydrophilic injections.

(6) Unless otherwise specified, any coloring agent must not be added solely for the purpose of coloring the preparations.

(7) Sodium chloride or other excipients may be added to aqueous injections to adjust them isotonic to blood or other body fluids. Acids or alkalis may be added to adjust the pH.

(8) Injections supplied in multiple-dose containers may be added sufficient amounts of suitable preservatives to prevent the growth of microorganisms.

(9) Unless otherwise specified, Injections and vehicles attached to preparations other than those used exclusively for intracutaneous, subcutaneous or intramuscular administration meet the requirements of Bacterial Endotoxins Test <*4.01*>. In the case where the

Bacterial Endotoxins Test <*4.01*> is not applicable, Pyrogen Test <*4.04*> may be applied instead.

(10) Unless otherwise specified, Injections and vehicles attached to preparations meet the requirements of Sterility Test <*4.06*>.

(11) Containers of Injections are colorless and meet the requirements of Test for Glass Containers for Injections <*7.01*>. Where specified in individual monographs, these containers may be replaced by colored containers meeting the requirements of Test for Glass Containers for Injections <*7.01*> or by plastic containers for aqueous injections meeting the requirements of Test Methods for Plastic Containers <*7.02*>.

(12) Unless otherwise specified, rubber closures used for glass containers of 100 mL or more of aqueous infusions meet the requirements of Test for Rubber Closure for Aqueous Infusions <*7.03*>.

(13) Unless otherwise specified, Injections and vehicles attached to preparations meet the requirements of Foreign Insoluble Matter Test for Injections <*6.06*>.

(14) Unless otherwise specified, Injections and vehicles attached to preparations meet the requirements of Insoluble Particulate Matter Test for Injections <*6.07*>.

(15) Unless otherwise specified, the actual volume of Injections meets the requirements of Test for Extractable Volume of Parenteral Preparations <*6.05*>.

(16) Unless otherwise specified, Injections to be dissolved or suspended before use meet the requirements of Uniformity of Dosage Units <*6.02*>.

(17) Among the suspensions for injection in unit-dose containers, the preparations that could impair the uniform dispersion upon standing have an appropriate uniformity.

(18) Suspensions for injection are usually not to be injected into the blood vessels or spinal cord, and emulsions for injection are not to be injected into the spinal cord.

(19) The maximum size of particles observed in suspensions for injection is usually not larger than 150 μm, and that of particles in emulsions for injection is usually not larger than 7 μm.

(20) The following information, unless otherwise specified, must be written on the package leaflet, or the container or wrapper.

(i) In cases where the vehicle is not specified, the name of the employed vehicle, with the exception of Water for Injection, sodium chloride solution not exceeding 0.9 w/v% and those vehicles in which acids or alkalis are used in order to adjust the pH.

(ii) In case of vehicle attached to preparation, the name of the vehicle, content volume, ingredients and quantities or ratios, and a statement of the presence of the vehicle on the outer container or outer wrapper.

(iii) Name and quantity of stabilizers, preservatives, and diluents if added. In the case where nitrogen or carbon dioxide is filled in the container to replace the air inside, a statement of this replacement is not required.

(21) For ampoules or other containers of 2 mL or less, the designations "injection", "for injection" and "aqueous suspension for injection" may be replaced by "inj.", "for inj." and "aq. susp. for inj.", respectively.

For ampoules or other containers of more than 2 mL and not exceeding 10 mL, made of glass or similar materials, the designations "injection", "for injection" and "aqueous suspension for injection" may be abbreviated in the same way as above, when the information is printed directly on the surface of ampoules or containers.

(22) Hermetic containers or tight containers which are able to prevent microbial contamination are usually used for the preparations. For the preparations susceptible to degradation by evaporation of water, a low-moisture-permeability container or packaging may be used.

3-1-1. Parenteral Infusions

(1) Parenteral Infusions are usually injections of not less than 100 mL, intended for intravenous administration.

(2) Parenteral Infusions are mainly administered for the purpose of water supply, correction of electrolyte abnormality and nutritional support, and they are also used by mixing with other injections for treatments by continual infusion.

3-1-2. Implants/Pellets

(1) Implants/Pellets are solid or gel-like form injections, intended for subcutaneous or intramuscular administration by means of an implant device or operative treatment, for the purpose of releasing active substance(s) for a long period of time.

(2) Implants/Pellets are usually prepared in a form of pellet, microsphere or gel using biodegradable polymers.

(3) Unless otherwise specified, Implants/Pellets meet the requirements of Uniformity of Dosage Units <*6.02*>.

(4) Implants/Pellets have an appropriate function of controlled release.

(5) Foreign Insoluble Matter Test for Injections, Insoluble Particulate Matter for Injections and Test for Extractable Volume of Parenteral Preparations are not required for Implants/Pellets.

3-1-3. Prolonged Release Injections

(1) Prolonged Release Injections are injections to be used for intramuscular administration, for the purpose of releasing active substance(s) for a long period of time.

(2) Prolonged Release Injections are usually prepared by dissolving or suspending active substance(s)

in a non-aqueous vehicle such as vegetable oil, or by suspending microspheres prepared with biodegradable polymers.

(3) Prolonged Release Injections have an appropriate function of controlled release.

4. Preparations for Dialysis

4-1. Dialysis Agents

(1) Dialysis Agents are preparations in liquid, or in solid which are to be dissolved before use, intended for peritoneal dialysis or hemodialysis.

They are classified into Peritoneal dialysis agents and Hemodialysis agents.

(2) Unless otherwise specified, Dialysis Agents meet the requirements of Bacterial Endotoxins Test <*4.01*>.

(3) The solid preparations which are to be dissolved before use among Dialysis agents have an appropriate uniformity of dosage units.

4-1-1. Peritoneal Dialysis Agents

(1) Peritoneal Dialysis Agents are sterile dialysis agents, intended to be used for peritoneal dialysis.

(2) Peritoneal Dialysis Agents are usually prepared by dissolving active substance(s) with suitable excipients in a vehicle to make a certain volume, or by filling active substance(s) combined with suitable excipients in a container, and sealing it. Sterilize if necessary. Every care should be taken to prevent microbial contamination. The overall processes from preparation to sterilization for preparing the agents should be completed as rapidly as possible, taking into consideration the composition of the agents and the storage conditions. The concentration of Peritoneal dialysis agents expressed in % represents w/v%. In the case of solid preparations which are dissolved before use, prepare as directed under 1-1. Tablets or 1-3. Granules.

(3) If necessary, pH adjusting agents, isotonic agents or other excipients may be added.

(4) Unless otherwise specified, the vehicle used for Peritoneal dialysis agents is Water for Injection.

(5) Unless otherwise specified, Peritoneal Dialysis Agents meet the requirements of Sterility Test <*4.06*>.

(6) Unless otherwise specified, Peritoneal Dialysis Agents meet the requirements of 4. Parenteral infusions under Test for Extractable Volume of Parenteral Preparations <*6.05*>. The mass (g) of content may convert to the volume (mL) by dividing by the density.

(7) Unless otherwise specified, Peritoneal Dialysis Agents meet the requirements of Foreign Insoluble Matter Test for Injections <*6.06*>.

(8) Unless otherwise specified, Peritoneal Dialysis Agents meet the requirements of Insoluble Particulate Matter Test for Injections <*6.07*>.

(9) Colorless containers meeting the requirements of Test for Glass Containers for Injections <*7.01*> are used for Peritoneal Dialysis Agents. Where specified otherwise, the colored containers meeting the requirements of Test for Glass Containers for Injections <*7.01*> or the plastic containers for aqueous injections meeting the requirements of Test Methods for Plastic Containers <*7.02*> may be used.

(10) Unless otherwise specified, the rubber closures of the containers meet the requirements of Test for Rubber Closure for Aqueous Infusions <*7.03*>.

(11) Hermetic containers, or tight containers which are able to prevent microbial contamination are usually used for Peritoneal Dialysis Agents. For the preparations susceptible to degradation by evaporation of water, a low-moisture-permeability container or packaging may be used.

4-1-2. Hemodialysis Agents

(1) Hemodialysis agents are dialysis agents to be used for hemodialysis.

(2) Hemodialysis Agents are usually prepared by dissolving active substance(s) with excipients in a vehicle to make a certain volume, or by filling active substance(s) with excipient(s) in a container. In the case of the solid preparations to be dissolved before use, prepare as directed under 1-1. Tablets or 1-3. Granules.

(3) If necessary, pH adjusting agents, isotonic agents or other excipients may be added.

(4) Unless otherwise specified, the vehicle used for Hemodialysis agents is Water for Injection or water suitable for dialysis.

(5) Tight containers which are able to prevent microbial contamination are usually used for Hemodialysis Agents. For the preparations susceptible to degradation by evaporation of water, a low-moisture-permeability container or packaging may be used.

5. Preparations for Inhalation

5-1. Inhalations

(1) Inhalations are preparations intended for administration as aerosols to the bronchial tubes or lung.

Inhalations are classified into Dry Powder Inhalers, Inhalation Liquid Preparations and Metered-dose Inhalers.

(2) For administration of Inhalations, suitable devices or apparatus are used, or they are placed in containers which have a appropriate function of inhalation device.

5-1-1. Dry Powder Inhalers

(1) Dry Powder Inhalers are preparations which deliver a constant respiratory intake, intended for administration as solid particle aerosols.

(2) Dry Powder Inhalers are usually prepared by pulverizing active substance(s) into fine particles. Where necessary, lactose or other suitable excipients are added to make homogenous mixture.

(3) Metered-dose types among Dry Powder Inhalers have an appropriate uniformity of delivered dose

of the active substance(s).

(4) The particles of active substance(s) in Dry Powder Inhalers have an aerodynamically appropriate size.

(5) Well-closed containers are usually used for Dry Powder Inhalers. For the preparations susceptible to degradation by moisture, a moisture-proof container or packaging may be used.

5-1-2. Inhalation Liquids and Solutions

(1) Inhalation Liquid Preparations are liquid inhalations which are administered by an inhalation device such as operating nebulizers.

(2) Inhalation Liquid Preparations are usually prepared by mixing active substance(s) with a vehicle and suitable isotonic agents and/or pH adjusting agents to make a solution or suspension, and by filtering where necessary.

(3) Sufficient amounts of suitable preservatives may be added to Inhalation Liquid Preparations to prevent the growth of microorganisms.

(4) Tight containers are usually used for Inhalation Liquid Preparations. For the preparations susceptible to degradation by evaporation of water, a low-moisture-permeability container or packaging may be used.

5-1-3. Metered-dose Inhalers

(1) Metered-dose Inhalers are preparations which deliver a constant dose of active substance(s) from the container together with propellant filled in.

(2) Metered-dose Inhalers are usually prepared by dissolving active substance(s) with a suitable dispersing agents and stabilizers in a vehicle to make a solution or suspension, and by filling in pressure-resistant containers together with liquid propellant, and setting metering valves.

(3) Metered-dose Inhalers have an appropriate uniformity of delivered dose of active substance(s).

(4) Particles of active substance(s) in Metered-dose Inhalers have an aerodynamically appropriate size.

(5) Pressure-resistant and hermetic containers are usually used for Metered-dose Inhalers.

6. Preparations for Ophthalmic Application

6-1. Ophthalmic Liquids and Solutions

(1) Ophthalmic Liquids and Solutions are sterile preparations of liquid, or solid to be dissolved or suspended before use, intended for application to the conjunctival sac or other ocular tissues.

(2) Ophthalmic Liquids and Solutions are usually prepared by dissolving, suspending active substance(s) in a vehicle after adding excipients to make a constant volume, or mixing active substance(s) and excipients, and filling into containers. The overall processes, from preparation to sterilization, should be completed with sufficient care to prevent microbial contamination as rapidly as possible, taking into consideration the composition of the preparations and the storage conditions. The concentration of active substance expressed in % represents w/v%.

Ophthalmic Liquids and Solutions to be dissolved or suspended before use and designated in the name as ''for ophthalmic application'' may be accompanied by a vehicle for dissolving or suspending the preparation (hereinafter referred to as ''vehicle attached to preparation'').

(3) Vehicles to prepare Ophthalmic Liquids and Solutions or vehicle attached to the preparations must be harmless in the amounts usually administered and must not interfere with the therapeutic efficacy of the active substance(s).

Vehicles for Ophthalmic Liquids and Solutions are classified into the following two groups.

(i) Aqueous vehicles: As the vehicles for the aqueous preparations Purified Water or suitable aqueous vehicles are used. For vehicles attached to the preparations sterilized Purified Water or sterilized aqueous vehicles are used.

(ii) Non-aqueous vehicles: As the vehicles for the non-aqueous preparations vegetable oils are usually used. Suitable organic solvents may be also used as the non-aqueous vehicles.

(4) Unless otherwise specified, any coloring agents must not be added solely for the purpose of coloring Ophthalmic Liquids and Solutions or vehicles attached to the preparations.

(5) Sodium chloride or other excipients may be added to Ophthalmic Liquids and Solutions to adjust them isotonic to lacrimal fluid. Acids or alkalis may be also added to adjust the pH.

(6) Unless otherwise specified, Ophthalmic Liquids and Solutions and vehicles attached to the preparations meet the requirements of Sterility Test 〈*4.06*〉.

(7) Sufficient amounts of appropriate preservatives to prevent the growth of microorganisms may be added to the preparations filled in multiple dose containers.

(8) Unless otherwise specified, Ophthalmic Liquids and Solutions prepared in aqueous solutions or the vehicles attached to the preparations meet the requirements of Foreign Insoluble Matter Test for Ophthalmic Solutions 〈*6.11*〉.

(9) Unless otherwise specified, Ophthalmic Liquids and Solutions and the vehicles attached to the preparations meet the requirements of Insoluble Particulate Matter Test for Ophthalmic Solutions 〈*6.08*〉.

(10) The maximum particle size observed in Ophthalmic suspensions is usually not larger than 75 μm.

(11) Transparent tight containers, which do not disturb the test of Foreign Insoluble Matter Test for Ophthalmic Solutions 〈*6.11*〉, are usually used for Ophthalmic Liquids and Solutions. For the preparations susceptible to degradation by evaporation of

water, a low-moisture-permeability container or packaging may be used.

6-2. Ophthalmic Ointments

(1) Ophthalmic Ointments are sterile preparations of semi-solid, intended for application to the conjunctival sac or other ocular tissues.

(2) Ophthalmic Ointments are usually prepared by mixing homogeneously solution of or finely powdered active substance(s) with petrolatum or other bases, and filling into containers. The overall processes, from preparation to sterilization, should be completed with sufficient care to prevent microbial contamination as rapidly as possible, taking into consideration the composition of the preparations and the storage conditions.

(3) Sufficient amounts of suitable preservatives may be added to Ophthalmic Ointments filled in multiple dose containers to prevent the growth of microorganisms.

(4) Unless otherwise specified, Ophthalmic Ointments meet the requirements of Sterility Test <*4.06*>, and unless otherwise specified, the test is carried out by the Membrane filtration method.

(5) Unless otherwise specified, Ophthalmic Ointments meet the requirements of Test for Metal Particles in Ophthalmic Ointments <*6.01*>.

(6) The maximum particle size of active substance(s) in Ophthalmic Ointments is usually not larger than 75 μm.

(7) Ophthalmic Ointments have a suitable viscosity for applying to the ocular tissues.

(8) Tight containers which are able to prevent microbial contamination are usually used for Ophthalmic Ointments. For the preparations susceptible to degradation by evaporation of water, a low-moisture-permeability container or packaging may be used.

7. Preparations for Otic Application

7-1. Ear Preparations

(1) Ear Preparations are liquid, semi-solid, or solid preparations which are to be dissolved or suspended before use, intended for application to the external or internal ear.

(2) Ear Preparations are usually prepared by filling in containers with liquids in which active substance(s) and excipients are dissolved or suspended in a vehicle to make a constant volume, or with powders in which active substance(s) and excipients are mixed. The overall processes, from preparation to sterilization, should be completed with sufficient care to prevent microbial contamination as rapidly as possible, taking into consideration the composition of the preparations and the storage conditions. The concentration of active substance of Ear Preparations expressed in % represents w/v%.

In the case where the sterile preparations are prepared, proceed as directed under 6-1. Ophthalmic Liquids and Solutions.

Ear Preparations which are to be dissolved or suspended before use and designated in the name as ''for otic preparation'' may be accompanied by a vehicle to dissolve or suspend (hereinafter referred to as ''vehicle attached to preparation'').

(3) Vehicles used for Ear Preparations or the vehicle attached to the preparation are classified into the following two groups.

(i) Aqueous vehicles: As the vehicles for the aqueous preparations or the vehicles attached to the preparations, Purified Water or suitable aqueous vehicles are used. For the sterile preparations, Sterilized Purified Water or suitable sterilized aqueous vehicles are used as the vehicle attached to the preparations.

(ii) Non-aqueous vehicles: As the vehicles for the non-aqueous preparations vegetable oils are usually used. Suitable organic solvents may be also used as non-aqueous vehicles.

(4) Unless otherwise specified, any coloring agents must not be added solely for the purpose of coloring Ear Preparations or vehicle attached to the preparations.

(5) Sufficient amounts of suitable preservatives to prevent the growth of microorganisms may be added to the preparations filled in multiple dose containers.

(6) Unless otherwise specified, sterile Ear preparations and the vehicles attached to the sterile preparations meet the requirements of Sterility Test <*4.06*>.

(7) Tight containers are usually used for Ear Preparations. For the preparations susceptible to degradation by evaporation of water, a low-moisture-permeability container or packaging may be used.

8. Preparations for Nasal Application

8-1. Nasal Preparations

(1) Nasal Preparations are preparations intended for application to the nasal cavities or nasal mucous membrane.

Nasal preparations are classified into Nasal dry powder inhalers and Nasal Liquid Preparations.

(2) Where necessary, Nasal Preparations are sprayed for inhalation by using a suitable atomizing device such as spray-pump.

(3) Unless otherwise specified, metered-dose type preparations among Nasal Preparations show the appropriate uniformity of delivered dose.

8-1-1. Nasal Dry Powder Inhalers

(1) Nasal Dry Powder Inhalers are fine powdered preparations, intended for application to the nasal cavities.

(2) Nasal Dry Powder Inhalers are usually prepared by pulverizing active substance(s) into moder-

ately fine particles, or by mixing homogeneously with excipients where necessary.

(3) Well-closed containers are usually used for Nasal Dry Powder Inhalers. For the preparations susceptible to degradation by moisture, a moisture-proof container or packaging may be used.

8-1-2. Nasal Liquids and Solutions

(1) Nasal Liquids and Solutions are liquid preparations, or solid preparations to be dissolved or suspended before use, intended for application to the nasal cavities.

(2) Nasal Liquids and Solutions are usually prepared by dissolving or suspending active substance(s) in a vehicle together with excipients, and filtering where necessary. Isotonic agents and/or pH adjusting agents may be used.

(3) Nasal Liquids and Solutions, which are to be dissolved or suspended before use and designated in the name as ''for nasal application'', may be accompanied by a vehicle to dissolve or suspend.

(4) Sufficient amounts of suitable preservatives to prevent the growth of microorganisms may be added to the preparations filled in multiple dose containers.

(5) Tight containers are usually used for Nasal Liquids and Solutions. For the preparations susceptible to degradation by evaporation of water, a low-moisture-permeability container or packaging may be used.

9. Preparations for Rectal Application

9-1. Suppositories for Rectal Application

(1) Suppositories for Rectal Application are semi-solid preparations of a desired shape and size, intended for intrarectal application, which release active substance(s) by melting at body temperature or dissolving or dispersing gradually in the secretions.

(2) Suppositories for Rectal Application are usually prepared by mixing homogenously active substance(s) and excipients such as dispersing agents and emulsifying agents, dissolving or suspending uniformly in a base which is liquefied by warming, filling a constant volume of the resultant material into containers, and molding it into a shape and size. Lipophilic bases or hydrophilic bases are usually used.

(3) Suppositories for Rectal Application are usually a conical- or spindle-shaped.

(4) Unless otherwise specified, Suppositories for Rectal Application meet the requirements of Uniformity of Dosage Units *<6.02>*.

(5) Suppositories for Rectal Application show an appropriate release.

(6) Well-closed containers are usually used for Suppositories for Rectal Application. For the preparations susceptible to degradation by moisture, a moisture-proof container or packaging may be used.

9-2. Semi-solid Preparations for Rectal Application

(1) Semi-solid Preparations for Rectal Application are preparations which are in a form of cream, gel or ointment intended for application to around or inside of the anus.

(2) Semi-solid Preparations for Rectal Application are usually prepared by emulsifying active substance(s) with excipients in Purified Water and oil component such as vaseline, or by homogenously mixing active substance(s) and excipients in a base of polymer gel or grease.

(i) Creams for rectal application: Prepare as directed under 11-5. Creams.

(ii) Gels for rectal application: Prepare as directed under 11-6. Gels.

(iii) Ointments for rectal application: Prepare as directed under 11-4. Ointments.

For the preparations which are apt to deteriorate, prepare before use.

(3) Sufficient amounts of suitable preservatives to prevent the growth of microorganisms may be added to the Preparations filled in multiple dose containers.

(4) Semi-solid Preparations for Rectal Application have a suitable viscosity for applying to the rectum.

(5) Tight containers are usually used for Semi-solid Preparations for Rectal Application. For the preparations susceptible to degradation by evaporation of water, a low-moisture-permeability container or packaging may be used.

9-3. Enemas for Rectal Application

(1) Enemas for Rectal Application are preparations in liquid form or viscous and gelatinous state, intended for application via the anus.

(2) Enemas for Rectal Application are usually prepared by dissolving or suspending active substance(s) in Purified Water or a suitable aqueous vehicle to make a given volume, and filling in containers. Dispersing agents, stabilizers and/or pH adjusting agents may be used.

(3) Tight containers are usually used for Enemas for Rectal Application. For the preparations susceptible to degradation by evaporation of water, a low-moisture-permeability container or packaging may be used.

10. Preparations for Vaginal Application

10-1. Tablets for Vaginal Use

(1) Tablets for Vaginal Use are solid preparations of a desired shape and size, intended for application to the vagina, which release active substance(s) by dissolving or dispersing gradually in the secretions.

(2) Tablets for Vaginal Use are usually prepared as directed under 1-1. Tablets.

(3) Unless otherwise specified, Tablets for Vaginal Use meet the requirements of Uniformity of Dosage

Units <*6.02*>.

(4) Tablets for Vaginal Use show an appropriate release.

(5) Well-closed containers are usually used for Tablets for Vaginal Use. For the preparations susceptible to degradation by moisture, a moisture-proof container or packaging may be used.

10-2. Suppositories for Vaginal Use

(1) Suppositories for Vaginal Use are semi-solid preparations of a desired shape and size, intended for application to the vagina, which release active substance(s) by melting at body temperature or by dissolving or dispersing gradually in the secretions.

(2) Suppositories for Vaginal Use are prepared according to 9-1. Suppositories for Rectal Application.

(3) Suppositories for Vaginal Use are usually spherical or ovoid shaped.

(4) Unless otherwise specified, Suppositories for Vaginal Use meet the requirements of Uniformity of Dosage Units <*6.02*>.

(5) Suppositories for Vaginal Use show an appropriate release.

(6) Well-closed containers are usually used for Suppositories for Vaginal Use. For the preparations susceptible to degradation by moisture, a moisture-proof container or packaging may be used.

11. Preparations for Cutaneous Application

(1) Preparations for Cutaneous Application also include Transdermal Systems which are intended for percutaneous absorption to deliver active substance(s) to the systemic circulation through the skin. The release rate of active substance(s) from Transdermal Systems is generally appropriately controlled.

11-1. Solid Preparations for Cutaneous Application

(1) Solid Preparations for Cutaneous Application are solid preparations intended for application to the skin (including scalp) or nails. Powders for Cutaneous Application are included in this category.

(2) Unless otherwise specified, Solid Preparations for Cutaneous Application in single-dose packages meet the requirements of Uniformity of Dosage Units <*6.02*>.

(3) Well-closed containers are usually used for Solid Preparations for Cutaneous Application. For the preparations susceptible to degradation by moisture, a moisture-proof container or packaging may be used.

11-1-1. Powders for Cutaneous Application

(1) Powders for Cutaneous Application are powdery solid preparations intended for external application.

(2) Powders for Cutaneous Application are usually prepared by mixing homogeneously active substance(s) and excipients such as diluents and pulverizing the mixture.

11-2. Liquids and Solutions for Cutaneous Application

(1) Liquids and Solutions for Cutaneous Application are liquid preparations intended for application to the skin (including scalp) or nails. Liniments and Lotions are included in this category.

(2) Liquids and Solutions for Cutaneous Application are usually prepared by mixing active substance(s) and excipients in a vehicle, and filtering if necessary.

For the preparations which are apt to deteriorate, prepare before use.

(3) Unless otherwise specified, Liquids and Solutions for Cutaneous Application in single-dose packages meet the requirements of Uniformity of Dosage Units <*6.02*>, except for emulsified or suspended preparations.

(4) Tight containers are usually used for Liquids and Solutions for Cutaneous Application. For the preparations susceptible to degradation by evaporation of water, a low-moisture-permeability container or packaging may be used.

11-2-1. Liniments

(1) Liniments are liquid or muddy preparations intended for external application to the skin by rubbing.

11-2-2. Lotions

(1) Lotions are external liquids in which active substance(s) are dissolved, emulsified or finely dispersed in an aqueous vehicle.

(2) Lotions are usually prepared by dissolving, suspending or emulsifying active substance(s) in Purified Water with excipients and making homogeneous as a whole.

(3) Lotions in which the components have separated out during storage may be used after mixing to re-homogenize them, provided that the active substance(s) has not deteriorated.

11-3. Sprays for Cutaneous Application

(1) Sprays for Cutaneous Application are preparations intended for spraying active substance(s) onto the skin in mists, powders, foams or paste state.

Sprays for Cutaneous Application are classified into Aerosols for Cutaneous Application and Pump Sprays for Cutaneous Application.

(2) Sprays for Cutaneous Application are usually prepared by dissolving or suspending active substance(s) in a vehicle, filtering where necessary, and filling in containers.

(3) Unless otherwise specified, metered-dose type sprays show an appropriate uniformity of delivered dose.

11-3-1. Aerosols for Cutaneous Application

(1) Aerosols for Cutaneous Application are sprays which atomize active substance(s) together with lique-

fied or compressed gas filled in containers.

(2) Aerosols for Cutaneous Application are usually prepared by dissolving or suspending active substance(s) in a vehicle, filling with liquefied propellants in pressure-resistant containers, and setting a continuous spray valve. If necessary, dispersing agents and stabilizers may be used.

(3) Pressure-resistant containers are usually used for Aerosols for Cutaneous Application.

11-3-2. Pump Sprays for Cutaneous Application

(1) Pump Sprays for Cutaneous Application are sprays which atomize active substance(s) in containers by pumping.

(2) Pump Sprays for Cutaneous Application are usually prepared by dissolving or suspending active substance(s) with excipients in a vehicle, filling in containers and setting pumps to the containers.

(3) Tight containers are usually used for Pump Sprays for Cutaneous Application. For the preparations susceptible to degradation by evaporation of water, a low-moisture-permeability container or packaging may be used.

11-4. Ointments

(1) Ointments are semi-solid preparations to be applied to the skin, which dissolve or disperse active substance(s) in a base. There are two types, hydrophobic ointments and hydrophilic ointments.

(2) Hydrophobic ointments are usually prepared by warming to melt hydrophobic bases such as fatty oils, waxes or paraffin, adding and mixing active substance(s) in the bases to be dissolved or dispersed, and kneading the whole to make homogeneous.

Hydrophilic ointments are usually prepared by warming to melt hydrophilic bases such as macrogol, adding and mixing active substance(s) in the bases, and kneading the whole to make homogeneous.

For Ointments which are apt to deteriorate, prepare before use.

(3) Ointments have a suitable viscosity for application to the skin.

(4) Tight containers are usually used for Ointments. For the preparations susceptible to degradation by evaporation of water, a low-moisture-permeability container or packaging may be used.

11-5. Creams

(1) Creams are semi-solid preparations to be applied to the skin, which are in the form of oil-in-water or water-in-oil emulsions. Hydrophobic preparations in the form of water-in-oil emulsions may be termed ''Oily creams''.

(2) Creams are usually prepared by mixing homogenously and emulsifying an oil-phase component and a water-phase component, both warmed, of which either one contains the active substance(s). These components have the following constituents.

Oil-phase component: Vaseline, fatty alcohols, etc., with or without emulsifying agent(s) or other suitable excipients.

Water-phase component: Purified Water with or without emulsifying agent(s) or other suitable excipients.

For Creams which are apt to deteriorate, prepare before use.

(3) Creams have a suitable viscosity for applying to the skin.

(4) Tight containers are usually used for Creams. For the preparations susceptible to degradation by evaporation of water, a low-moisture-permeability container or packaging may be used.

11-6. Gels

(1) Gels are gelatinous preparations intended for application to the skin.

There are Aqueous Gels and Oily Gels.

(2) Gels are usually prepared by the following methods.

(i) Aqueous Gels: To active substance(s) add polymers, other excipients and Purified Water, dissolve or suspend, and gelatinize by warming and cooling or by adding a gelatinizing agents.

(ii) Oily Gels: To active substance(s) add liquid oily bases such as glycols, fatty alcohols and other excipients, and mix.

(3) Gels have a suitable viscosity for application to the skin.

(4) Tight containers are usually used for Gels. For the preparations susceptible to degradation by evaporation of water, a low-moisture-permeability container or packaging may be used.

11-7. Patches

(1) Patches are preparations intended to be attached on the skin.

Patches are classified into Tapes/Plasters and Cataplasms/Gel Patches.

(2) Patches are usually prepared by mixing active substance(s) homogeneously with a base such as a polymer or a mixture of polymers, spreading on a backing layer or liner, and cutting into a given size. Percutaneous absorption type preparations may be prepared by using a release rate-controlling membrane. Where necessary, adhesive agents or penetration enhancers may be used.

(3) Unless otherwise specified, Patches of Transdermal Systems meet the requirements of Uniformity of Dosage Units <*6.02*>.

(4) Unless otherwise specified, Patches meet the requirement of Methods of Adhesion Testing <*6.12*>.

(5) Unless otherwise specified, Patches meet the requirement of Release Test for Preparations for

Cutaneous Application <6.13>.

11-7-1. Tapes

(1) Tapes are patches which are prepared with bases containing practically no water.

Plasters are included in this category.

(2) Tapes are usually prepared by mixing homogeneously active substance(s) with or without excipients and a base of non water-soluble natural or synthetic polymers such as resins, plastics or rubber, and spreading on a cloth or spreading and sealing on a cloth or plastic film, cutting into a given size. The preparations may be also prepared by filling a mixture of active substance(s) and a base with or without other excipients in releasers composed with a release-controlling film, supporter and liner.

(3) Well-closed containers are usually used for Tapes. For the preparations susceptible to degradation by moisture, a moisture-proof container or packaging may be used.

11-7-2. Cataplasms/Gel Patches

(1) Cataplasms/Gel Patches are patches using water containing bases.

(2) Cataplasms/Gel patches are usually prepared by mixing active substance(s), Purified Water, and Glycerin or other liquid materials, or by mixing and kneading natural or synthetic polymers, which are soluble in water or absorbent of water, with Purified Water, adding active substance(s), mixing the whole homogeneously, spreading on a cloth or film, and cutting into a given size.

(3) Tight containers are usually used for Cataplasms/Gel Patches. For the preparations susceptible to degradation by evaporation of water, a low-moisture-permeability container or packaging may be used.

[4] Monographs for Preparations Related to Crude Drugs

Preparations Related to Crude Drugs

(1) Preparations related to crude drugs are preparations mainly derived from crude drugs. Extracts, Pills, Spirits, Infusions and Decoctions, Teabags, Tinctures, Aromatic Waters, and Fluidextracts are included in this category.

Definitions, methods of preparations, test methods, containers and packaging, and storage of these preparations are described in this chapter.

(2) The descriptions of the test methods and the containers and packaging in this chapter are fundamental requirements, and the preparation methods represent commonly used methods.

1. Extracts

(1) Extracts are preparations, prepared by concentrating extractives of crude drugs. There are following two kinds of extracts.

(i) Viscous extracts

(ii) Dry extracts

(2) Unless otherwise specified, Extracts are usually prepared as follows.

(i) Crude drugs, pulverized to suitable sizes, are extracted for a certain period of time with suitable solvents by means of cold extraction or warm extraction, or by percolation as directed in (ii) of (2) under 6. Tinctures. The extractive is filtered, and the filtrate is concentrated or dried by a suitable method to make a millet jelly-like consistency for the viscous extracts, or to make crushable solid masses, granules or powder for the dry extracts.

Extracts, which are specified the content of active substance(s), are prepared by assaying active substance(s) in a portion of sample and adjusting, if necessary, to specified strength with suitable diluents.

(ii) Weigh crude drugs, pulverized to suitable sizes, according to the prescription and heat for a certain period of time after adding 10 – 20 times amount of water. After separating the solid and liquid by centrifugation, the extractive is concentrated or dried by a suitable method to make a millet jelly-like consistency for the viscous extracts, or to make crushable solid masses, granules or powder for the dry extracts.

(3) Extracts have order and taste derived from the crud drugs used.

(4) Unless otherwise specified, Extracts meet the requirements of Heavy Metals Limit Test <1.07> when the test solution and the control solution are prepared as follows.

Test solution: Ignite 0.30 g of Extracts to ash, add 3 mL of dilute hydrochloric acid, warm, and filter. Wash the residue with two 5-mL portions of water. Neutralize the combined filtrate and washings (indicator: a drop of phenolphthalein TS) by adding ammonia TS until the color of the solution changes to pale red, filter where necessary, and add 2 mL of dilute acetic acid and water to make 50 mL.

Control solution: Proceed with 3 mL of dilute hydrochloric acid in the same manner as directed in the preparation of the test solution, and add 3.0 mL of Standard Lead Solution and water to make 50 mL.

(5) Tight containers are used for these preparations.

2. Pills

(1) Pills are spherical preparations, intended for oral administration.

(2) Pills are usually prepared by mixing drug substance(s) uniformly with diluents, binders, disintegrators or other suitable excipient(s) and rolling into spherical form by a suitable method. They may be coated with a coating agent by a suitable method.

(3) Unless otherwise specified, Pills comply with Disintegration Test <*6.09*>.

(4) Well-closed or tight containers are usually used for these preparations.

3. Spirits

(1) Spirits are fluid preparations, usually prepared by dissolving volatile drug substance(s) in ethanol or in a mixture of ethanol and water.

(2) Spirits should be stored remote from fire.

(3) Tight containers are used for these preparations.

4. Infusions and Decoctions

(1) Infusions and Decoctions are fluid preparations, usually obtained by macerating crude drugs in water.

(2) Infusions and Decoctions are usually prepared by the following method.

Cut crude drugs into a size as directed below, and transfer suitable amounts to an infusion or decoction apparatus.

Leaves, flowers and whole plants: Coarse cutting
Woods, stems, barks, roots and rhizomes
: Moderately fine cutting
Seeds and fruits: Fine cutting

(i) Infusions: Usually, damp 50 g of crude drugs with 50 mL of water for about 15 minutes, pour 900 mL of hot water to them, and heat for 5 minutes with several stirrings. Filter through a cloth after cooling.

(ii) Decoctions: Usually, heat one-day dose of crude drugs with 400 – 600 mL of water until to lose about a half amount of added water spending more than 30 minutes, and filter through a cloth while warm.

Prepare Infusions or Decoctions when used.

(3) These preparations have odor and taste derived from the crude drugs used.

(4) Tight containers are usually used for these preparations.

5. Teabags

(1) Teabags are preparations, usually packed one-day dose or one dose of crude drugs cut into a size of between coarse powder and coarse cutting in paper or cloth bags.

(2) Teabags are usually used according to the preparation method as directed under 4. Infusions and Decoctions.

(3) Well-closed or tight containers are usually used for these preparations.

6. Tinctures

(1) Tinctures are liquid preparations, usually prepared by extracting crude drugs with ethanol or with a mixture of ethanol and purified water.

(2) Unless otherwise specified, Tinctures are usually prepared from coarse powder or fine cuttings of crude drugs by means of either maceration or percolation as described below.

(i) Maceration: Place crude drugs in a suitable container, and add an amount of a solvent, equivalent to the same volume or about three-fourths of the volume of the crude drugs. Stopper container, and allow the container to stand for about 5 days or until the soluble constituents have satisfactorily dissolved at room temperature with occasional stirring. Separate the solid and liquid by centrifugation or other suitable methods. In the case where about three-fourths volume of the solvent is added, wash the residue with a suitable amount of the solvent, and squeeze the residue, if necessary. Combine the extract and washings, and add sufficient solvent to make up the volume. In the case where the total volume of the solvent is added, sufficient amounts of the solvent may be added to make up for reduced amount, if necessary. Allow the mixture to stand for about 2 days, and obtain a clear liquid by decantation or filtration.

(ii) Percolation: Pour solvent in small portions to crude drugs placed in a container, and mix well to moisten the crude drugs. Stopper container, and allow it to stand for about 2 hours at room temperature. Pack the contents as tightly as possible in an appropriate percolator, open the lower opening, and slowly pour sufficient solvent to cover the crude drugs. When the percolate begins to drip, close the opening, and allow the mixture to stand for 2 to 3 days at room temperature. Then, open the opening, and allow the percolate to drip at a rate of 1 to 3 mL per minute. Add an appropriate quantity of the solvent to the percolator, and continue to percolate until the desired volume has passed. Mix thoroughly, allow standing for 2 days, and obtain a clear liquid by decantation or filtration. The time of standing and the flow rate may be varied depending on the kind and amount of crude drugs to be percolated.

Tinctures, prepared by either of the above methods and specified the content of marker constituent or ethanol, are prepared by assaying the content using a portion of the sample and adjusting the content with a sufficient amount of the percolate or solvent as required on the basis of the result of the assay.

(3) Tinctures should be stored remote from fire.

(4) Tight containers are used for these preparations.

7. Aromatic Waters

(1) Aromatic Waters are clear liquid preparations, saturated essential oils or other volatile substances in water.

(2) Unless otherwise specified, Aromatic Waters are usually prepared by the following process.

Shake thoroughly for 15 minutes 2 mL of an essential oil or 2 g of a volatile substance with 1000 mL of lukewarm purified water, set the mixture aside for 12 hours or longer, filter through moistened filter paper, and add purified water to make 1000 mL. Alternatively, incorporate thoroughly 2 mL of an essential oil or 2 g of a volatile substance with sufficient talc, refined siliceous earth or pulped filter-paper, add 1000 mL of purified water, agitate thoroughly for 10 minutes, and then filter the mixture. To obtain a clear filtrate repeat the filtration if necessary, and add sufficient purified water passed through the filter paper to make 1000 mL.

(3) Aromatic Waters have odor and taste derived from the essential oils or volatile substances used.

(4) Tight containers are used for these preparations.

8. Fluidextracts

(1) Fluidextracts are liquid percolates of crude drugs, usually prepared so that each mL contains soluble constituents from 1 g of the crude drugs. Where the content is specified, it takes precedence.

(2) Unless otherwise specified, Fluidextracts are usually prepared from coarse powder or fine cutting of crude drugs by either of following maceration or percolation.

(i) Maceration: Place a certain amounts of crude drugs in a suitable vessel, add a solvent to cover the crude drugs, close the vessel, and allow the vessel to stand at room temperature with occasional stirring for about 5 days or until the soluble constituents have satisfactorily dissolved. Separate the solid and liquid by centrifugation or other suitable method. Usually, reserve a volume of the liquid equivalent to about three-fourths of the total volume, and use it as the first liquid. Wash the residue with appropriate amount of the solvent, combine the washings and the remaining of the first liquid, concentrate if necessary, mix with the first liquid, and use it as solution (A). To the solution (A) add the solvent, if necessary, to make equal amount of the mass of the crude drugs. Allow the mixture to stand for about 2 days, and collect a clear liquid by decantation or filtration.

(ii) Percolation: Mix well 1000 g of the crude drugs with the first solvent to moisten them, close the container, and allow it to stand for about 2 hours at room temperature. Transfer the content to a suitable percolator, stuff it as tightly as possible, open the lower opening of the percolator, and slowly pour the second solvent to cover the crude drugs. Close the lower opening when the solvent begins to drop, and allow the mixture to stand for 2 to 3 days at room temperature. Open the lower opening, and allow the percolate to run out at the rate of 0.5 to 1.0 mL per minute.

Set aside the first 850 mL of the percolate as the first percolate. Add the second solvent to the percolator, then drip the percolate, and use it as the second percolate.

The period of standing and the flow rate during percolation may be varied depending on the kind and the amount of crude drugs used. The flow rate is usually regulated as follows, depending on the using amount of crude drugs.

Mass of crude drug	Volume of solution running per minute
Not more than 1000 g	0.5 – 1.0 mL
Not more than 3000 g	1.0 – 2.0 mL
Not more than 10000 g	2.0 – 4.0 mL

Concentrate the second percolate, taking care not to lose the volatile substances of the crude drug, mix with the first percolate, and use it as solution (A). To the solution (A) add the second solvent to make 1000 mL, and allow the mixture to stand for about 2 days. Decant the supernatant liquid or filter the liquid to obtain a clear solution.

Fluidextracts for which the content of marker constituent or ethanol is specified are obtained by adjusting the content with a sufficient amount of the second solvent as required on the basis of the result of the assay made with a portion of the solution (A).

(3) Fluidextracts have odor and taste derived from the crude drugs used.

(4) Unless otherwise specified, Fluidextracts meet the requirements of Heavy Metals Limit Test *<1.07>* when the test solution and the control solution are prepared as follows.

Test solution: Ignite 1.0 g of Fluidextracts to ash, add 3 mL of dilute hydrochloric acid, warm, and filter. Wash the residue with two 5-mL portions of water. Neutralize the combined filtrate and washings (indicator: a drop of phenolphthalein TS) by adding ammonia TS until the color of the solution changes to pale red, filter if necessary, and add 2 mL of the dilute acetic acid and water to make 50 mL.

Control solution: Proceed with 3 mL of dilute hydrochloric acid in the same manner as directed in the preparation of the test solution, and add 3.0 mL of Standard Lead Solution and water to make 50 mL.

(5) Tight containers are used for these preparations.
