# Supplementary material for: Effects of the Kampo medicine Yokukansan for perioperative anxiety and postoperative pain in women undergoing breast surgery: A randomized, controlled trial
Source: PLoS One. 2021 Nov 24;16(11):e0260524. doi: 10.1371/journal.pone.0260524 (PMC8612547; doi:10.1371/journal.pone.0260524)
Supplement: S4 File — (PDF) [file pone.0260524.s005.pdf]

2020年11月（改訂第10版）

日本標準商品分類番号875200

医薬品インタビューフォーム

日本病院薬剤師会のＩＦ記載要領（1998年9月）に準拠して作成

漢方製剤

ツムラ抑肝散エキス顆粒（医療用）

TSUMURA Yokukansan Extract Granules for Ethical Use

54®

剤形	顆粒剤
規格・含量	本品7.5g中、下記の割合の混合生薬の乾燥エキス3.25gを含有する。<br>日局ソウジュツ 4.0g　日局トウキ 3.0g<br>日局ブクリョウ 4.0g　日局サイコ 2.0g<br>日局センキュウ 3.0g　日局カンゾウ 1.5g<br>日局チョウトウコウ 3.0g
一般名（処方名）	抑肝散
製造販売承認年月日<br>薬価基準収載・発売年月日	製造販売承認年月日：昭和61年３月５日<br>薬価基準収載年月日：昭和61年10月30日<br>発売年月日：昭和61年10月30日
開発・製造販売（輸入）・提携・販売会社名	製造販売　株式会社ツムラ
医薬情報担当者の連絡先	
問い合わせ窓口	株式会社ツムラ　お客様相談窓口<br>TEL 0120-329-970　FAX 03-5574-6610<br>医療関係者向けホームページ<br>http://www.tsumura.co.jp/password/top.htm

本ＩＦは2014年11月改訂の添付文書の記載に基づき改訂した。
最新の添付文書情報は、PMDAホームページ「医薬品に関する情報」
http://www.pmda.go.jp/ にてご確認ください。

IF利用の手引きの概要－日本病院薬剤師会－

1．医薬品インタビューフォーム作成の経緯

当該医薬品について製薬企業の医薬情報担当者（以下、MRと略す）等にインタビューし、当該医薬品の評価を行うのに必要な医薬品情報源として使われていたインタビューフォームを、昭和63年日本病院薬剤師会（以下、日病薬と略す）学術第2小委員会が「医薬品インタビューフォーム」（以下、IFと略す）として位置付けを明確化し、その記載様式を策定した。そして、平成10年日病薬学術第3小委員会によって新たな位置付けとIF記載要領が策定された。

2．IFとは

IFは「医療用医薬品添付文書等の情報を補完し、薬剤師等の医療従事者にとって日常業務に必要な医薬品の適正使用や評価のための情報あるいは薬剤情報提供の裏付けとなる情報等が集約された総合的な医薬品解説書として、日病薬が記載要領を策定し、薬剤師等のために当該医薬品の製薬企業に作成及び提供を依頼している学術資料」と位置付けられる。

しかし、薬事法の規制や製薬企業の機密等に関わる情報、製薬企業の製剤意図に反した情報及び薬剤師自らが評価・判断・提供すべき事項等はIFの記載事項とはならない。

3．IFの様式・作成・発行

規格はA4判、横書きとし、原則として9ポイント以上の字体で記載し、印刷は一色刷りとする。表紙の記載項目は統一し、原則として製剤の投与経路別に作成する。IFは日病薬が策定した「IF記載要領」に従って記載するが、本IF記載要領は、平成11年1月以降に承認された新医薬品から適用となり、既発売品については「IF記載要領」による作成・提供が強制されるものではない。また、再審査及び再評価（臨床試験実施による）がなされた時点ならびに適応症の拡大等がなされ、記載内容が大きく異なる場合にはIFが改訂・発行される。

4．IF利用にあたって

IF策定の原点を踏まえ、MRへのインタビュー、自己調査のデータを加えてIFの内容を充実させ、IFの利用性を高めておく必要がある。

MRへのインタビューで調査・補足する項目として、開発の経緯、製剤的特徴、薬理作用、臨床成績、非臨床試験等の項目が挙げられる。また、随時改訂される使用上の注意等に関する事項に関しては、当該医薬品の製薬企業の協力のもと、医療用医薬品添付文書、お知らせ文書、緊急安全性情報、Drug Safety Update（医薬品安全対策情報）等により薬剤師等自らが加筆、整備する。そのための参考として、表紙の下段にIF作成の基となった添付文書の作成又は改訂年月を記載している。なお適正使用や安全確保の点から記載されている「臨床成績」や「主な外国での発売状況」に関する項目等には承認外の用法・用量、効能・効果が記載されている場合があり、その取扱いには慎重を要する。

目　　次

Ⅰ．概要に関する項目
1．開発の経緯……1
2．製品の特徴及び有用性……1

Ⅱ．名称に関する項目
1．販売名
⑴和名……2
⑵洋名……2
⑶名称の由来……2
2．一般名
⑴和名（命名法）……2
⑵洋名（命名法）……2
3．構造式又は示性式……2
4．分子式及び分子量……3
5．化学名（命名法）……3
6．慣用名、別名、略号、記号番号……3
7．ＣＡＳ登録番号……3

Ⅲ．有効成分に関する項目
1．有効成分の規制区分……4
2．物理化学的性質
⑴外観・性状……4
⑵溶解性……4
⑶吸湿性……4
⑷融点（分解点）、沸点、凝固点……4
⑸酸塩基解離定数……4
⑹分配係数……4
⑺その他の主な示性値……4
3．有効成分の各種条件下における安定性……4
4．有効成分の確認試験法……5
5．有効成分の定量法……5

Ⅳ．製剤に関する項目
1．剤形
⑴剤形の区別及び性状……6
⑵製剤の物性……6
⑶識別コード……6
⑷pH、浸透圧比、粘度、無菌の旨及び安定なpH域等……6
2．製剤の組成
⑴有効成分（活性成分）の含量……6
⑵添加物……6
3．製剤の各種条件下における安定性……6

4．他剤との配合変化（物理化学的変化）…… 7
5．混入する可能性のある夾雑物…… 7
6．溶出試験…… 8
7．製剤中の有効成分の確認試験法…… 9
8．製剤中の有効成分の定量法…… 9
9．容器の材質…… 9
10．その他……10

Ⅴ．治療に関する項目
1．効能又は効果……11
2．用法及び用量……11
3．臨床成績
⑴臨床効果……11
⑵臨床薬理試験：忍容性試験……11
⑶探索的試験：用量反応探索試験……11
⑷検証的試験
1)無作為化平行用量反応試験……11
2)比較試験……11
3)安全性試験……11
4)患者・病態別試験……11
⑸治療的使用
1)使用成績調査・特定使用成績調査・製造販売後臨床試験……11
2)承認条件として実施予定の内容又は実施した試験の概要……11

Ⅵ．薬効薬理に関する項目
1．薬理学的に関連ある化合物又は化合物群……12
2．薬理作用
⑴作用部位・作用機序……12
⑵薬効を裏付ける試験成績……12

Ⅶ．薬物動態に関する項目
1．血中濃度の推移・測定法
⑴治療上有効な血中濃度……13
⑵最高血中濃度到達時間……13
⑶通常用量での血中濃度……13
⑷中毒症状を発現する血中濃度……14
2．薬物速度論的パラメータ
⑴吸収速度定数……14
⑵バイオアベイラビリティ……14
⑶消失速度定数……14
⑷クリアランス……14
⑸分布容積……14

⑹血漿蛋白結合率……14
3．吸収……14
4．分布
⑴血液－脳関門通過性……15
⑵胎児への移行性……15
⑶乳汁中への移行性……15
⑷髄液への移行性……15
⑸その他の組織への移行性……15
5．代謝
⑴代謝部位及び代謝経路……15
⑵代謝に関与する酵素（CYP450等）の分子種……16
⑶初回通過効果の有無及びその割合……16
⑷代謝物の活性の有無及び比率……16
⑸活性代謝物の速度論的パラメータ……16
6．排泄
⑴排泄部位……16
⑵排泄率……16
⑶排泄速度……16
7．透析等による除去率
⑴腹膜透析……16
⑵血液透析……16
⑶直接血液灌流……16

Ⅷ．安全性（使用上の注意等）に関する項目
1．警告内容とその理由……17
2．禁忌内容とその理由……17
3．効能・効果に関連する使用上の注意とその理由……17
4．用法・用量に関連する使用上の注意とその理由……17
5．慎重投与内容とその理由……17
6．重要な基本的注意とその理由及び処置方法……17
7．相互作用
⑴併用禁忌とその理由……18
⑵併用注意とその理由……18
8．副作用
⑴副作用の概要……18
1)重大な副作用と初期症状……18
2)その他の副作用……19
⑵項目別副作用発現頻度及び臨床検査値異常一覧……20
⑶基礎疾患、合併症、重症度及び手術の有無等背景別の副作用発現頻度……22
⑷薬物アレルギーに対する注意及び試験法……23
9．高齢者への投与……23
10．妊婦、産婦、授乳婦等への投与……23

11．小児等への投与……23
12．臨床検査結果に及ぼす影響……23
13．過量投与……23
14．適用上及び薬剤交付時の注意（患者等に留意すべき必須事項等）……23
15．その他の注意……23
16．その他……23

Ⅸ．非臨床試験に関する項目
1．一般薬理……24
2．毒性
⑴単回投与毒性試験……24
⑵反復投与毒性試験……24
⑶生殖発生毒性試験……24
⑷その他の特殊毒性……24

Ⅹ．取扱い上の注意等に関する項目
1．有効期間又は使用期限……25
2．貯法・保存条件……25
3．薬剤取扱い上の注意点……25
4．承認条件……25
5．包装……25
6．同一成分・同効薬……25
7．国際誕生年月日……25
8．製造・輸入承認年月日及び承認番号……25
9．薬価基準収載年月日……25
10．効能・効果追加、用法・用量変更追加等の年月日及びその内容……25
11．再審査結果、再評価結果公表年月日及びその内容……25
12．再審査期間……26
13．長期投与の可否……26
14．厚生労働省薬価基準収載医薬品コード……26
15．保険給付上の注意……26

Ⅺ．文献
1．引用文献……27
2．その他の参考文献……28

Ⅻ．参考資料
主な外国での発売状況……28

XIII．備考
その他の関連資料……28

Ⅰ. 概要に関する項目

１. 開発の経緯

本剤は、漢方の古典（保嬰撮要）に記載されている薬方（抑肝散）をツムラ独自の乾式造粒法により服用しやすい顆粒剤として製剤化し、これを「厚生省薬務局薬審2第120号通知（S.60.5.31付）」に基づき製造承認申請し、承認された医療用漢方エキス製剤「ツムラ抑肝散エキス顆粒（医療用）」である。

２. 製品の特徴及び有用性

⑴本剤は7種類の生薬（ソウジュツ、ブクリョウ、センキュウ、チョウトウコウ、トウキ、サイコ、カンゾウ）を水のみで煎出し、噴霧乾燥法により製した乾燥エキスを、有機溶媒や水を一切使用しないツムラ独自の乾式造粒法により顆粒剤とした漢方エキス製剤である。

⑵効能又は効果は、以下のとおりである。

虚弱な体質で神経がたかぶるものの次の諸症：

神経症、不眠症、小児夜なき、小児疳症

⑶薬効薬理試験で、抗不安様作用、攻撃性抑制作用及び睡眠障害改善作用が確認されている。

II. 名称に関する項目

1. 販売名

(1)和名

ツムラ抑肝散エキス顆粒（医療用）

(2)洋名

TSUMURA Yokukansan Extract Granules for Ethical Use

(3)名称の由来

ツムラ　　株式会社ツムラの商号

抑肝散　　経絡の一種である肝経の機能失調により起こるとされる発熱、興奮、ひきつけ等を抑制するという効能に基づいて名付けられた。

2. 一般名

(1)和名（命名法）

抑肝散

(2)洋名（命名法）

yokukansan

3. 構造式又は示性式

［参考］

7種類の生薬を一定の割合で配合したものを抽出して得たエキス製剤で、ソウジュツ由来のβ−オイデスモール、センキュウ由来の(*E*)−フェルラ酸、サイコ由来のサイコサポニン類（b_1, b_2)、カンゾウ由来のグリチルリチン酸等が含有される。その代表的な成分の構造式を以下に示す。

fuc=fucose
glc A = glucuronic acid
glc = glucose

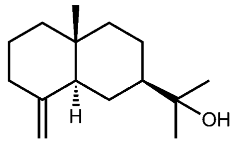

β-eudesmol
（ソウジュツ）

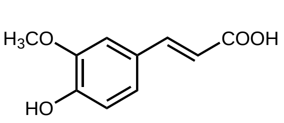

(*E*)−ferulic acid
（センキュウ）

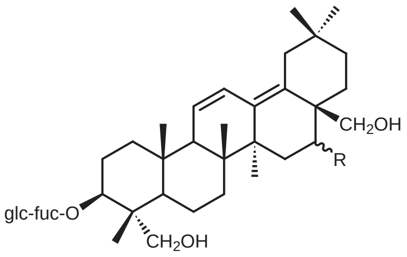

saikosaponin b_1：R＝β-OH
saikosaponin b_2：R＝α-OH
（サイコ）

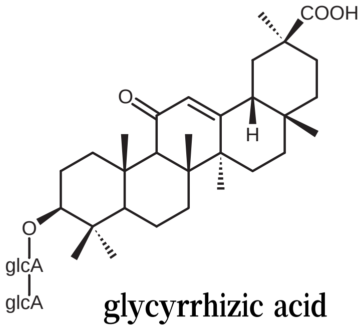

glycyrrhizic acid
（カンゾウ）

４．分子式及び分子量

該当しない

５．化学名（命名法）

該当しない

６．慣用名、別名、略号、記号番号

記号番号　TJ－54

７．CAS登録番号

該当しない

III. 有効成分に関する項目

1．有効成分の規制区分

該当しない

2．物理化学的性質

⑴外観・性状

黄褐色の粉末で、特異なにおいがあり、味はわずかに甘い。

⑵溶解性

該当しない

⑶吸湿性

臨界相対湿度は特定できない。参考のため、吸湿曲線を示す。

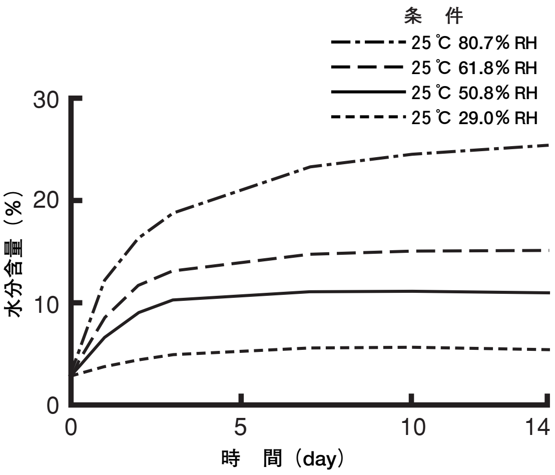

エキス粉末の吸湿曲線

⑷融点（分解点）、沸点、凝固点

該当しない

⑸酸塩基解離定数

該当しない

⑹分配係数

該当しない

⑺その他の主な示性値

該当しない

3．有効成分の各種条件下における安定性

吸湿性が高い。［**「III. 2．⑶吸湿性」**を参照すること。］

４．有効成分の確認試験法

「Ⅳ．製剤に関する項目」に記載した試験方法によりエキス粉末中の下記構成生薬を確認する。

ソウジュツ、センキュウ、チョウトウコウ、トウキ、サイコ、カンゾウ

５．有効成分の定量法

「Ⅳ．製剤に関する項目」に記載した試験方法によりエキス粉末中の無水エタノールエキス及び下記含量規格成分の含量を求める。

「グリチルリチン酸」、「サイコサポニン b_2」、「(E)-フェルラ酸」

Ⅳ．製剤に関する項目

１．剤形

⑴剤形の区別及び性状

剤　形	性　状		
	色	におい	味
顆粒剤	淡灰褐色	特異なにおい	わずかに甘くて渋い

⑵製剤の物性

顆粒の安息角、分散度等

見掛密度（g/mL）		安息角（度）	分散度（%）
ゆるみ	固め		
約0.67	約0.77	約37	約6.1

パウダーテスターによる（25℃ 50％RH）

⑶識別コード

ツムラ／54

⑷ pH、浸透圧比、粘度、無菌の旨及び安定なpH域等

［溶液のpH］

本品2.5gに水50mLを加えてかき混ぜた液のpHは約5.4である。

２．製剤の組成

⑴有効成分（活性成分）の含量

本品7.5g中、下記の割合の混合生薬の乾燥エキス3.25ｇを含有する。

日局ソウジュツ	4.0ｇ	日局トウキ	3.0ｇ
日局ブクリョウ	4.0ｇ	日局サイコ	2.0ｇ
日局センキュウ	3.0ｇ	日局カンゾウ	1.5ｇ
日局チョウトウコウ	3.0ｇ		

⑵添加物

添加物として、日局ステアリン酸マグネシウム、日局乳糖水和物を含有する。

３．製剤の各種条件下における安定性

⑴製剤の性状・含量規格成分等の変化

保存形態	保存条件	結果*
アルミ分包	室温5カ年	変化なし
ポリエチレンボトル	室温5カ年	変化なし

＊項目（性状、確認試験、含量規格成分の定量値、製剤試験等）

(2) 製剤の外観の変化（開封後）

保存形態	保存条件	結　果
未 包 装	25℃ 81%RH 1日	ケーキング
	25℃ 62%RH 1日	ケーキング
	25℃ 51%RH 1日	ケーキング
	25℃ 29%RH 14日	変化なし
グラシン紙	25℃ 81%RH 7日	ケーキング
	25℃ 62%RH 7日	ケーキング
	25℃ 51%RH 14日	ケーキング
	25℃ 29%RH 14日	変化なし

1)未包装状態におけるエキス顆粒の吸湿曲線

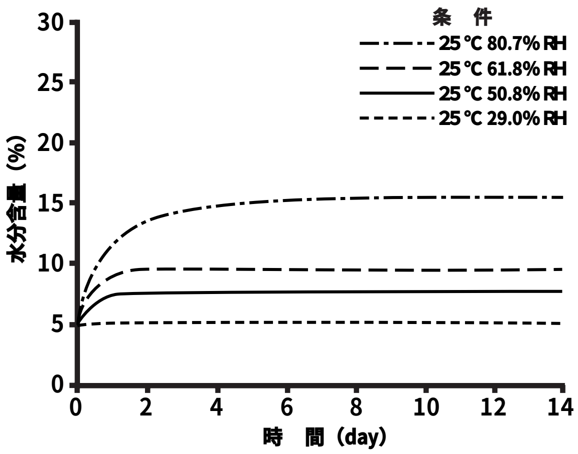

2)グラシン紙分包の吸湿曲線

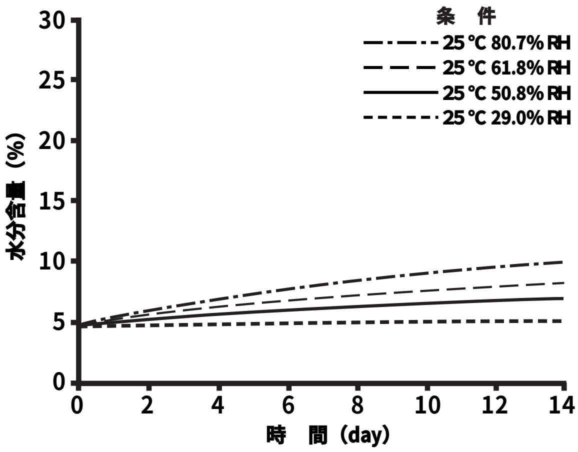

4．他剤との配合変化（物理化学的変化）

該当資料なし

5．混入する可能性のある夾雑物

副生成物、分解物の特定はできない。

６．溶出試験

本品中の含量規格成分溶出挙動の測定結果を以下に示す。

なお、溶出率は製剤一回服用量中の含量規格成分含量の測定値を100%とした。

試験方法：日本薬局方「溶出試験法（パドル法）」による。

条件　試験サンプル量：2.5g

回転速度：100rpm

試験液：精製水

(1)グリチルリチン酸の溶出挙動

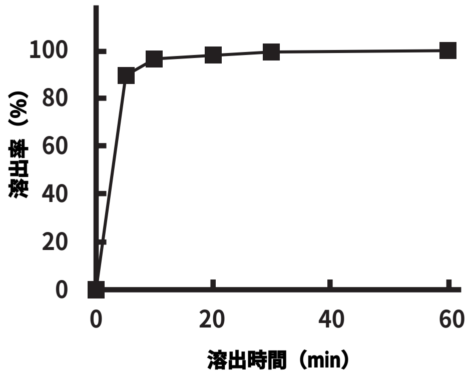

(2)サイコサポニンb_2の溶出挙動

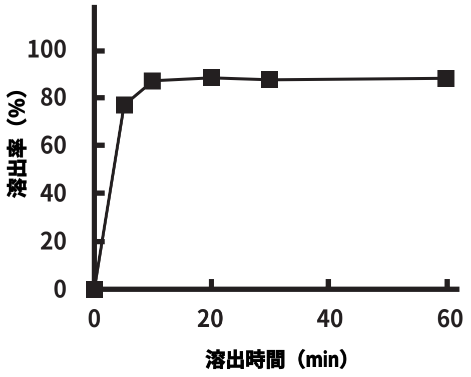

(3)(*E*)−フェルラ酸の溶出挙動

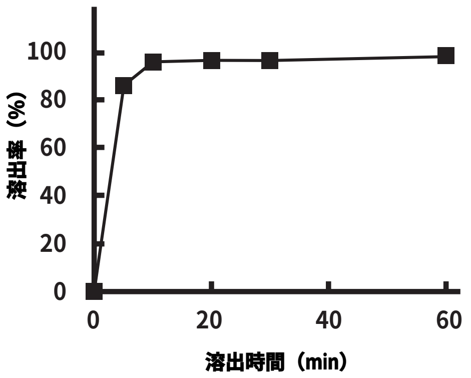

７．製剤中の有効成分の確認試験法

⑴ソウジュツ

薄層クロマトグラフィーにより「ソウジュツ」由来のスポットを確認する。

⑵センキュウ・トウキ

薄層クロマトグラフィーにより「センキュウ」及び「トウキ」由来のスポットを確認する。

⑶チョウトウコウ

薄層クロマトグラフィーにより「チョウトウコウ」由来のスポットを確認する。

⑷サイコ

薄層クロマトグラフィーにより「サイコ」由来のスポットを確認する。

⑸カンゾウ

薄層クロマトグラフィーにより「カンゾウ」由来のスポットを確認する。

８．製剤中の有効成分の定量法

⑴無水エタノールエキス

本品中に含まれるエキス粉末由来のエタノール（99.5）可溶成分の量を把握する試験である。

抽 出 溶 媒：エタノール（99.5）

操　作　法：日本薬局方、一般試験法「生薬試験法」のエキス含量の項「エーテルエキス定量法」に準じる。

⑵グリチルリチン酸

本品中に含まれる「カンゾウ」由来のグリチルリチン酸を、液体クロマトグラフィーにより定量する。

⑶サイコサポニンb_2

本品中に含まれる「サイコ」由来のサイコサポニンb_2を、液体クロマトグラフィーにより定量する。

⑷(E)-フェルラ酸

本品中に含まれる「センキュウ」由来の(E)-フェルラ酸を、液体クロマトグラフィーにより定量する。

９．容器の材質

プラスチック容器：ポリエチレン・ポリプロピレン・ナイロン

ア　ル　ミ　分　包：アルミ箔・ポリエチレン・ポリエチレンテレフタレート

ア　　ル　　ミ　　袋：アルミ箔・ポリエチレン・ポリエチレンテレフタレート

10．その他

(1)微生物限度

生菌数限度値は日本薬局方、参考情報の「非無菌医薬品の微生物学的品質特性」に記載の「非無菌製剤の微生物学的品質に対する許容基準値」中の「経口(非水性製剤)」に準ずる。
特定微生物に関しては、同項にて例示されている大腸菌に加え、日本薬局方、微生物限度試験法に試験法が収載されているサルモネラを設定している。

項目		試験方法	限度値
生菌数試験	総好気性微生物数	日本薬局方、微生物限度試験法に準ずる。	10^3 CFU/g 以下
	総真菌数		10^2 CFU/g 以下
特定微生物試験	大腸菌		認めない
	サルモネラ		認めない

CFU：Colony Forming Unit

(2)無機元素含量

以下に、製剤中の代表的無機元素の実測例を示す。
測定は誘導結合プラズマ質量分析（ICP - MS）法で実施した。

元素	Na	K	Ca	Mg	P	Fe	Al	Zn	I
一日換算量 (mg)	1.5	68.4	7.5	8.1	11.4	0.3	0.8	0.03	0.001 未満
当量 (mEq)	0.06	1.75	0.37	0.66	1.11	0.01	0.08	0.001	0.00001 未満

Ⅴ. 治療に関する項目

1. 効能又は効果

虚弱な体質で神経がたかぶるものの次の諸症：

神経症、不眠症、小児夜なき、小児疳症

［参考］

使用目標：体力中等度の人で、神経過敏で興奮しやすく、怒りやすい、イライラする、眠れないなどの精神神経症状を訴える場合に用いる。

1)おちつきがない、ひきつけ、夜泣きなどのある小児

2)眼瞼痙攣や手足のふるえなどを伴う場合

3)腹直筋の緊張している場合

2. 用法及び用量

通常、成人1日7.5gを2〜3回に分割し、食前又は食間に経口投与する。なお、年齢、体重、症状により適宜増減する。

3. 臨床成績

⑴臨床効果

該当資料なし

⑵臨床薬理試験：忍容性試験

該当資料なし

⑶探索的試験：用量反応探索試験

該当資料なし

⑷検証的試験

1)無作為化平行用量反応試験

該当資料なし

2)比較試験

該当資料なし

3)安全性試験

該当資料なし

4)患者・病態別試験

該当資料なし

⑸治療的使用

1)使用成績調査・特定使用成績調査・製造販売後臨床試験

該当資料なし

2)承認条件として実施予定の内容又は実施した試験の概要

該当資料なし

Ⅵ．薬効薬理に関する項目

１．薬理学的に関連ある化合物又は化合物群

［参考］

「Ⅱ．３．構造式又は示性式」を参照すること。

２．薬理作用

⑴作用部位・作用機序

本剤は、以下の作用により薬理効果を示すことが示唆されている。

1)攻撃性抑制作用

①グルタミン酸放出抑制作用

亜鉛欠乏ラットに経口投与したところ、海馬細胞外液グルタミン酸濃度の上昇並びに海馬スライス標本におけるグルタミン酸神経終末開口放出が抑制された[1)]。

②グルタミン酸取込是正作用

チアミン欠乏下のラット培養アストロサイトにおいて、グルタミン酸取込能の低下、グルタミン酸トランスポーターのmRNA並びに蛋白の発現低下を改善した（*in vitro*）[2)]。

③セロトニン 2A 受容体ダウンレギュレーション作用

正常マウスに経口投与したところ、前頭前野セロトニン 2A 受容体発現量が低下し、セロトニン 2A 受容体作動薬（ジメトキシヨードアンフェタミン）誘発首振り運動が抑制された[3)]。

④セロトニン 1A 受容体刺激作用

・パラクロロアンフェタミン処置ラット[4)]及び隔離ストレスマウス[5)]に経口投与したところ、攻撃性が抑制され、その作用はセロトニン1A受容体拮抗薬（WAY-100635）で消失した。

・*in vitro*受容体結合試験において、セロトニン1A受容体部分刺激作用を示した[6)]。

・電気ショックラットにおける不安抑制作用は、WAY-100635 で消失した[7)]。

⑵薬効を裏付ける試験成績

1)抗不安様作用

正常マウスに経口投与したところ、高架式十字迷路実験において抗不安様作用を示した[8)]。この作用は脳虚血ラット（経口）[9)] 及び老齢ラット（混餌）[10)] でも認められた。

2)攻撃性抑制作用

アミロイド前駆体蛋白過剰発現マウス（混餌）[11)] 、アミロイドβ蛋白脳室内注入マウス（経口）[12)] 及び亜鉛欠乏マウス（飲水）[13)] に投与したところ、攻撃性が抑制された。

3)睡眠障害改善作用

隔離ストレスマウスに経口投与したところ、ペントバルビタール誘発睡眠時間の短縮が抑制された[14)] 。

Ⅶ. 薬物動態に関する項目

1．血中濃度の推移・測定法

(1)治療上有効な血中濃度

該当資料なし

(2)最高血中濃度到達時間

「1．(3)通常用量での血中濃度」を参照すること。

(3)通常用量での血中濃度

［参考］グリチルレチン酸

健常人12例にカンゾウエキス顆粒剤（カンゾウエキス粉末約0.63g*を含む）を単回経口投与した際の血漿中において、グリチルリチン酸の代謝物であるグリチルレチン酸は下図のように推移した[15]。

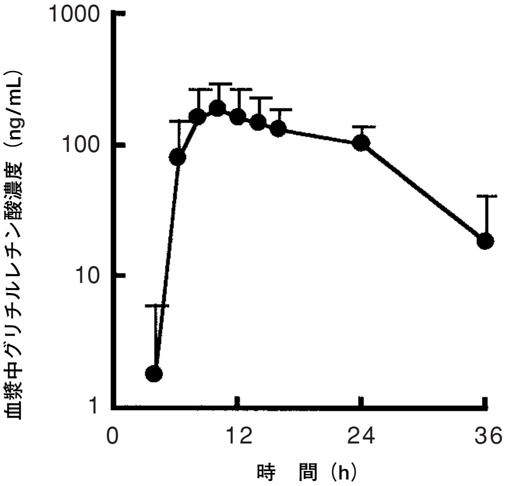

［平均値+標準偏差, HPLCにより測定］

グリチルレチン酸の薬物速度論的パラメータ

t_{max}[15] (h)	C_{max}[15] (ng/mL)	$t_{1/2}$[15] (h)	AUC(0－lim)[15] (ng･h/mL)	吸収速度定数[15] (h^{-1})	消失速度定数[15] (h^{-1})	みかけの分布容積[15] (L)	みかけのクリアランス[15] (mL/min)	血清蛋白結合率[16] (%)
12.8±5.7[a)]	228.0±105.7[a)]	7.4±2.7[b)]	3248.9±1146.0[a)]	0.812±1.417[c)]	0.096±0.039[c)]	119.5±80.6[c)]	154.6±39.1[c)]	＞99.9

平均値±標準偏差　a) n=12, b) n=9, c) n=11

*グリチルリチン酸として60.2mg、グリチルレチン酸に換算すると34.4mg含有

［参考］構成生薬中の各種成分のラットにおける薬物動態

1) β-オイデスモール (ソウジュツの成分)[17]

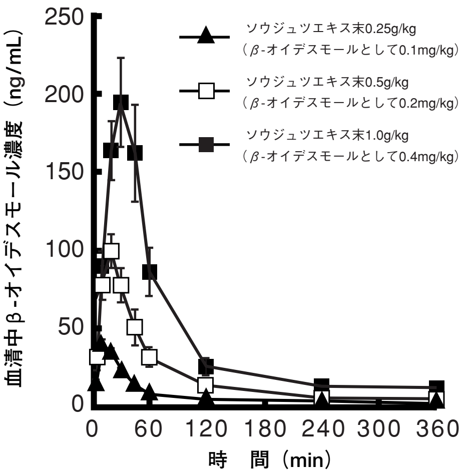

ラットにソウジュツエキス末を経口投与した際の血清中β-オイデスモール濃度推移
［平均値±標準誤差, n=6～8, EIAにより測定］

2)サイコサポニンb_2 (サイコの成分)[18]

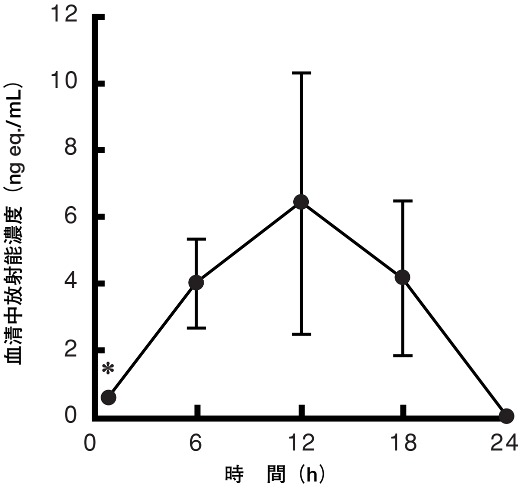

ラットに^{3}H-サイコサポニンb_2 1mg/kgを経口投与した際の血清中放射能濃度推移
［平均値±標準偏差, n=3, *;平均値, n=2, ^{3}H-サイコサポニンb_2 放射活性測定］

⑷中毒症状を発現する血中濃度

該当資料なし

2．薬物速度論的パラメータ

⑴吸収速度定数

「1．⑶通常用量での血中濃度」を参照すること。

⑵バイオアベイラビリティ

「1．⑶通常用量での血中濃度」を参照すること。

⑶消失速度定数

「1．⑶通常用量での血中濃度」を参照すること。

⑷クリアランス

「1．⑶通常用量での血中濃度」を参照すること。

⑸分布容積

「1．⑶通常用量での血中濃度」を参照すること。

⑹血漿蛋白結合率

「1．⑶通常用量での血中濃度」を参照すること。

3．吸収

該当資料なし

4．分布

⑴血液-脳関門通過性

該当資料なし

⑵胎児への移行性

該当資料なし

⑶乳汁中への移行性

該当資料なし

⑷髄液への移行性

該当資料なし

⑸その他の組織への移行性

該当資料なし

5．代謝

⑴代謝部位及び代謝経路

［参考］

1)マウス腸内細菌によるサイコの成分サイコサポニンb_2の代謝経路（*in vitro*）[19)]

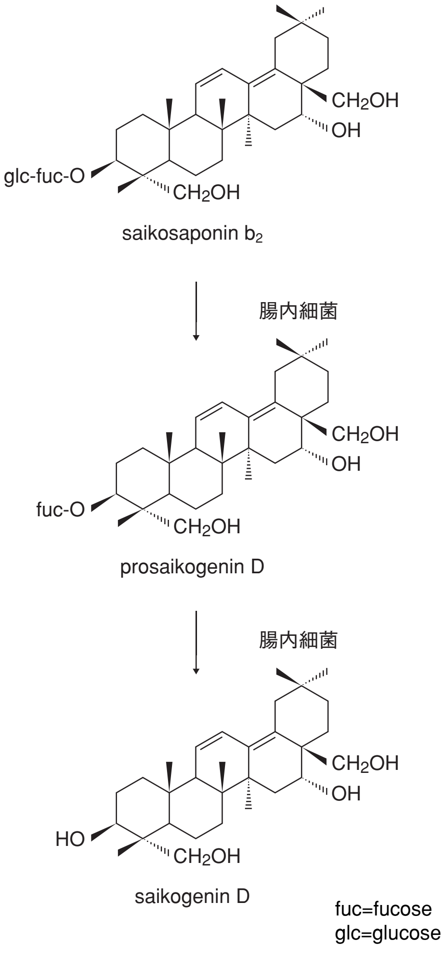

2）ヒト腸内細菌によるカンゾウの成分グリチルリチン酸の代謝経路（*in vitro*）[20]

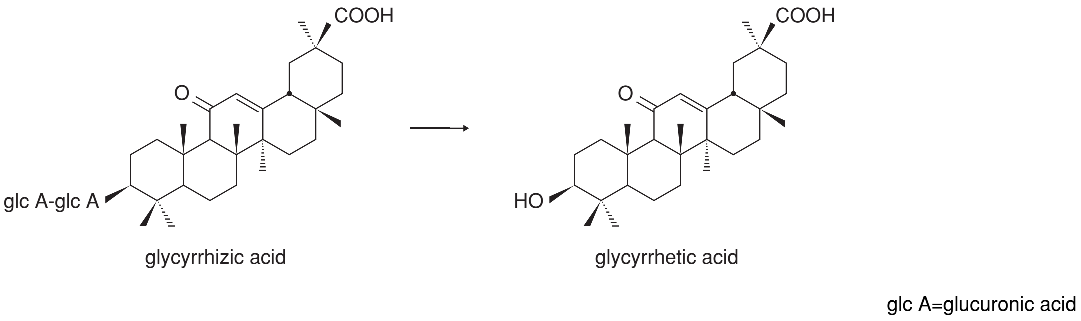

⑵代謝に関与する酵素（CYP450等）の分子種

該当資料なし

⑶初回通過効果の有無及びその割合

該当資料なし

⑷代謝物の活性の有無及び比率

該当資料なし

⑸活性代謝物の速度論的パラメータ

該当資料なし

6．排泄

⑴排泄部位

該当資料なし

⑵排泄率

該当資料なし

⑶排泄速度

該当資料なし

7．透析等による除去率

⑴腹膜透析

該当資料なし

⑵血液透析

該当資料なし

⑶直接血液灌流

該当資料なし

Ⅷ. 安全性（使用上の注意等）に関する項目

1. 警告内容とその理由

特になし

2. 禁忌内容とその理由

特になし

3. 効能・効果に関連する使用上の注意とその理由

「Ⅴ. 治療に関する項目」を参照すること。

4. 用法・用量に関連する使用上の注意とその理由

「Ⅴ. 治療に関する項目」を参照すること。

5. 慎重投与内容とその理由

**慎重投与（次の患者には慎重に投与すること）**
(1)著しく胃腸の虚弱な患者［食欲不振、胃部不快感、悪心、下痢等があらわれることがある。］
(2)食欲不振、悪心、嘔吐のある患者［これらの症状が悪化するおそれがある。］

［理由］

(1)本剤にはセンキュウ[21)~25)]・トウキ[25)26)]が含まれているため、著しく胃腸の虚弱な患者に投与すると食欲不振、胃部不快感、悪心、下痢等があらわれるおそれがあり、記載した。また、本剤によると思われる消化器症状が文献・学会で報告されている[27)]。

(2)本剤にはセンキュウ・トウキが含まれているため、食欲不振、悪心、嘔吐のある患者に投与するとこれらの症状が悪化するおそれがあり、記載した。

6. 重要な基本的注意とその理由及び処置方法

(1)本剤の使用にあたっては、患者の証（体質・症状）を考慮して投与すること。なお、経過を十分に観察し、症状・所見の改善が認められない場合には、継続投与を避けること。
(2)本剤にはカンゾウが含まれているので、血清カリウム値や血圧値等に十分留意し、異常が認められた場合には投与を中止すること。
(3)他の漢方製剤等を併用する場合は、含有生薬の重複に注意すること。

［理由］

(1)医療用漢方製剤のより一層の適正使用を図るため、漢方医学の考え方を考慮して使用する旨を記載した。

(2)カンゾウは多くの処方に配合されているため、過量になりやすく副作用があらわれやすくなるので記載した。

(3)医療用漢方製剤を併用する場合には、重複生薬の量的加減が困難であるため記載した。

7．相互作用

⑴併用禁忌とその理由

特になし

⑵併用注意とその理由

併用注意（併用に注意すること）

薬剤名等	臨床症状・措置方法	機序・危険因子
(1)カンゾウ含有製剤<br>(2)グリチルリチン酸及びその塩類を含有する製剤	偽アルドステロン症があらわれやすくなる。また、低カリウム血症の結果として、ミオパチーがあらわれやすくなる。（「重大な副作用」の項参照）	グリチルリチン酸は尿細管でのカリウム排泄促進作用があるため、血清カリウム値の低下が促進されることが考えられる。

［理由］

厚生省薬務局長より通知された昭和53年2月13日付薬発第158号「グリチルリチン酸等を含有する医薬品の取り扱いについて」に基づき、上記の併用注意を記載した。

8．副作用[28)]

⑴副作用の概要

副作用発現頻度調査（2012年10月～2014年3月）において、3,156例中、136例（4.3%）162件に臨床検査値の異常を含む副作用が報告された。

1)**重大な副作用と初期症状**

1)**間質性肺炎（頻度不明）**：発熱、咳嗽、呼吸困難、肺音の異常等があらわれた場合には、本剤の投与を中止し、速やかに胸部X線、胸部CT等の検査を実施するとともに副腎皮質ホルモン剤の投与等の適切な処置を行うこと。

［理由］

本剤によると思われる間質性肺炎の企業報告の集積により、厚生労働省内で検討された結果、上記の副作用を記載した。（平成22年7月6日付薬食安発0706第1号「使用上の注意」の改訂について　に基づく改訂）

［処置方法］

直ちに投与を中止し、胸部X線撮影・CT・血液ガス圧測定等により精検し、ステロイド剤投与等の適切な処置を行うこと。

2)**偽アルドステロン症（頻度不明）**：低カリウム血症、血圧上昇、ナトリウム・体液の貯留、浮腫、体重増加等の偽アルドステロン症があらわれることがあるので、観察（血清カリウム値の測定等）を十分に行い、異常が認められた場合には投与を中止し、カリウム剤の投与等の適切な処置を行うこと。

3)**心不全（0.1％未満）**：心不全があらわれることがあるので、観察を十分に行い、体液貯留、急激な体重増加、心不全症状・徴候（息切れ、心胸比拡大、胸水等）が認められた場合には、投与を中止し、適切な処置を行うこと。

4)**ミオパチー、横紋筋融解症（頻度不明）**：低カリウム血症の結果として、ミオパチー、横紋筋融解症があらわれることがあるので、観察を十分に行い、脱力感、筋力低下、筋肉痛、四肢痙攣・麻痺、CK（CPK）上昇、血中及び尿中のミオグロビン上昇が認められた場合には投与を中止し、カリウム剤の投与等の適切な処置を行うこと。

［理由］〔2）4）共〕

厚生省薬務局長より通知された昭和53年2月13日付薬発第158号「グリチルリチン酸等を含有する医薬品の取り扱いについて」に基づき、また、本剤によると思われる横紋筋融解症、CK（CPK）の上昇等の企業報告の集積により、厚生労働省内で検討された結果、上記の副作用を記載した。平成26年2月18日付薬食安発0218第1号「使用上の注意」の改訂についてに基づく改訂）

3）本剤によると思われる心不全、うっ血性心不全の企業報告の集積により、厚生労働省内で検討された結果、上記の副作用を記載した。（平成26年2月18日付薬食安発0218第1号「使用上の注意」の改訂について　に基づく改訂）

［処置方法］

原則的に投与を中止し、血清カリウム値のほか、血中アルドステロン・レニン活性、CK(CPK)、血中及び尿中ミオグロビン等の検査を行う。偽アルドステロン症、心不全・うっ血性心不全、またはミオパチー・横紋筋融解症と判定された場合は、症状の種類や程度により適切な治療を行うこと。低カリウム血症に対してはカリウム剤の補給等により電解質バランスの適正化を行う[29)30)]。

5)**肝機能障害、黄疸（頻度不明）**：AST（GOT）、ALT（GPT）、Al-P、γ-GTP等の著しい上昇を伴う肝機能障害、黄疸があらわれることがあるので、観察を十分に行い、異常が認められた場合には投与を中止し、適切な処置を行うこと。

［理由］

本剤によると思われる肝機能障害、黄疸の企業報告の集積により、厚生労働省内で検討された結果、上記の副作用を記載した。（平成22年7月6日付薬食安発0706第1号「使用上の注意」の改訂について　に基づく改訂）

［処置方法］

原則的には投与中止により改善するが、病態に応じて適切な処置を行うこと。

2)その他の副作用

	0.1～5%未満	0.1%未満
過敏症[注1)]		発疹、発赤、瘙痒等
肝臓	肝機能異常	
消化器	食欲不振、胃部不快感、悪心、下痢等	
精神神経系	傾眠	
その他	低カリウム血症、浮腫、血圧上昇、倦怠感	

注1）このような症状があらわれた場合には投与を中止すること。

過敏症

［理由］

本剤によると思われる発疹、発赤、瘙痒等が報告されている（企業報告）ため、上記の副作用を記載した。

［処置方法］
原則的には投与中止にて改善するが、必要に応じて抗ヒスタミン剤・ステロイド剤投与等の適切な処置を行うこと。

肝臓

［理由］
副作用発現頻度調査の結果、本剤によると思われる症例が報告されているため、上記副作用を記載した。

［処置方法］
原則的には投与中止により改善するが、病態に応じて適切な処置を行うこと。

消化器

［理由］
本剤にはセンキュウ[22)~26)]・トウキ[23)~26)]が含まれているため、食欲不振、胃部不快感、悪心、下痢等の消化器症状があらわれるおそれがある。また、本剤によると思われる消化器症状が文献・学会で報告されている[28)]。これらのため、上記の副作用を記載した。

［処置方法］
原則的には投与中止により改善するが、病態に応じて適切な処置を行うこと。

精神神経系（傾眠）

［理由］
副作用発現頻度調査の結果、本剤によると思われる症例が報告されているため、上記副作用を記載した。

［処置方法］
原則的には投与中止により改善するが、病態に応じて適切な処置を行うこと。

その他（低カリウム血症に係る症状）

「8.⑴副作用の概要　1）重大な副作用　2）偽アルドステロン症」を参照すること。

その他（倦怠感）

［理由］
副作用発現頻度調査の結果、本剤によると思われる症例が報告されているため、上記副作用を記載した。

［処置方法］
原則的には投与中止により改善するが、病態に応じて適切な処置を行うこと。

⑵項目別副作用発現頻度及び臨床検査値異常一覧

副作用発現頻度

	全体		投与期間26週未満		投与期間26週以上		投与期間不明	
	発現例数	割合（%）	発現例数	割合（%）	発現例数	割合（%）	発現例数	割合（%）
安全性解析対象集団	3156		1690		1343		123	
副作用発現症例数（例）	136		97		34		5	
副作用発現件数（件）	162		112		45		5	
副作用発現症例率（%）	4.3%		5.7%		2.5%		4.1%	

副作用等の種類(器官別大分類:SOC、基本語:PT)※	副作用の種類別発現症例数(症例数(率%))			
心臓障害	4 0.1%	3 0.2%	1 0.1%	0
大動脈弁閉鎖不全症	1 0.0%	1 0.1%	0	0
急性心不全	1 0.0%	0	1 0.1%	0
動悸	2 0.1%	2 0.1%	0	0
内分泌障害	1 0.0%	1 0.1%	0	0
偽アルドステロン症	1 0.0%	1 0.1%	0	0
胃腸障害	19 0.6%	18 1.1%	1 0.1%	0
腹部不快感	4 0.1%	4 0.2%	0	0
上腹部痛	1 0.0%	1 0.1%	0	0
便秘	1 0.0%	1 0.1%	0	0
下痢	6 0.2%	5 0.3%	1 0.1%	0
胃腸障害	1 0.0%	1 0.1%	0	0
悪心	6 0.2%	6 0.4%	0	0
嘔吐	1 0.0%	1 0.1%	0	0
一般・全身障害および投与部位の状態	32 1.0%	26 1.5%	5 0.4%	1 0.8%
胸部不快感	1 0.0%	1 0.1%	0	0
異常感	2 0.1%	2 0.1%	0	0
倦怠感	4 0.1%	4 0.2%	0	0
浮腫	18 0.6%	14 0.8%	3 0.2%	1 0.8%
末梢性浮腫	5 0.2%	3 0.2%	2 0.1%	0
発熱	1 0.0%	1 0.1%	0	0
口渇	1 0.0%	1 0.1%	0	0
肝胆道系障害	10 0.3%	8 0.5%	2 0.1%	0
肝機能異常	8 0.3%	7 0.4%	1 0.1%	0
肝障害	2 0.1%	1 0.1%	1 0.1%	0
臨床検査	15 0.5%	7 0.4%	7 0.5%	1 0.8%
血中クロール減少	1 0.0%	0	1 0.1%	0
血中クロール増加	1 0.0%	0	1 0.1%	0
血中クレアチニン増加	1 0.0%	0	1 0.1%	0
血中カリウム減少	6 0.2%	3 0.2%	3 0.2%	0
血圧上昇	5 0.2%	4 0.2%	1 0.1%	0
血中尿素増加	1 0.0%	1 0.1%	0	0
血中アルカリホスファターゼ増加	4 0.1%	0	3 0.2%	1 0.8%
代謝および栄養障害	45 1.4%	23 1.4%	20 1.5%	2 1.6%
高ナトリウム血症	1 0.0%	0	1 0.1%	0
低カリウム血症	41 1.3%	21 1.2%	18 1.3%	2 1.6%
食欲減退	4 0.1%	2 0.1%	2 0.1%	0
筋骨格系および結合組織障害	2 0.1%	2 0.1%	0	0
関節痛	1 0.0%	1 0.1%	0	0
筋肉痛	1 0.0%	1 0.1%	0	0
神経系障害	14 0.4%	12 0.7%	2 0.1%	0
浮動性めまい	2 0.1%	2 0.1%	0	0
味覚異常	1 0.0%	1 0.1%	0	0
ジスキネジー	1 0.0%	1 0.1%	0	0
頭痛	1 0.0%	1 0.1%	0	0
片頭痛	1 0.0%	1 0.1%	0	0
鎮静	1 0.0%	0	1 0.1%	0
傾眠	7 0.2%	6 0.4%	1 0.1%	0
精神障害	4 0.1%	3 0.2%	0	1 0.8%
気力低下	1 0.0%	1 0.1%	0	0
性嗜好異常	1 0.0%	1 0.1%	0	0
徘徊癖	1 0.0%	1 0.1%	0	0
落ち着きのなさ	1 0.0%	1 0.1%	0	0
異常行動	1 0.0%	0	0	1 0.8%
腎および尿路障害	3 0.1%	3 0.2%	0	0
頻尿	2 0.1%	2 0.1%	0	0
多尿	1 0.0%	1 0.1%	0	0

皮膚および皮下組織障害	2　0.1%	1　0.1%	1　0.1%	0
紅斑	1　0.0%	1　0.1%	0	0
そう痒症	1　0.0%	1　0.1%	0	0
発疹	1　0.0%	0	1　0.1%	0
蕁麻疹	1　0.0%	1　0.1%	0	0
血管障害	2　0.1%	0	2　0.1%	0
高血圧	2　0.1%	0	2　0.1%	0

⑶基礎疾患、合併症、重症度及び手術の有無等背景別の副作用発現頻度

患者背景別の副作用発現頻度

背景項目		例数	発現例数	割合(%)	Fisherの正確な検定(p値)
安全性解析対象集団		3,156	136	4.3%	-
性別	男	1,172	48	4.1%	0.717
	女	1,984	88	4.4%	
年齢(歳)	65歳未満	1,189	33	2.8%	0.001
	65歳以上	1,967	103	5.2%	
入院外来別	入院	484	41	8.5%	0.000
	外来	2,525	87	3.4%	
	入院⇔外来	147	8	5.4%	
BMI(kg/m^2)	25.0未満	1,392	67	4.8%	0.865
	25.0以上	217	11	5.1%	
	不明	1,547	58	3.7%	-
アレルギー歴	なし	3,066	129	4.2%	0.109
	あり	90	7	7.8%	
肝機能障害	なし	2,365	99	4.2%	0.128
	軽度	143	10	7.0%	
	中等度	16	2	12.5%	
	重度	2	0		
	不明	630	25	4.0%	-
腎機能障害	なし	2,318	95	4.1%	0.040
	軽度	157	14	8.9%	
	中等度	31	2	6.5%	
	重度	10	0		
	不明	640	25	3.9%	-
合併症	なし	1,954	55	2.8%	0.000
	あり	1,202	81	6.7%	
平均1日投与量(g)	≦1.0	3	0		0.000
	1.0＜～≦2.5	391	3	0.8%	
	2.5＜～≦5.0	914	45	4.9%	
	5.0＜～≦7.5	1,707	81	4.7%	
	7.5＜	18	2	11.1%	
	不明	123	5	4.1%	-

⑷薬物アレルギーに対する注意及び試験法

「8．副作用 ⑴副作用の概要 2)その他の副作用 過敏症」を参照すること。

9．高齢者への投与

一般に高齢者では生理機能が低下しているので減量するなど注意すること。

［理由］

平成4年4月1日付薬安第30号「高齢者への投与に関する医療用医薬品の使用上の注意の記載について」に基づき上記の使用上の注意を記載した。

10．妊婦、産婦、授乳婦等への投与

妊娠中の投与に関する安全性は確立していないので、妊婦又は妊娠している可能性のある婦人には、治療上の有益性が危険性を上回ると判断される場合にのみ投与すること。

11．小児等への投与

小児等に対する安全性は確立していない。［使用経験が少ない］

12．臨床検査結果に及ぼす影響

該当資料なし

13．過量投与

該当資料なし

14．適用上及び薬剤交付時の注意（患者等に留意すべき必須事項等）

《適用上の注意》

［参考］

本剤の投与にあたっては、「**Ⅴ．治療に関する項目**」の「使用目標」並びに「**Ⅷ．安全性（使用上の注意等）に関する項目 5．慎重投与内容とその理由**」を参照すること。

《薬剤交付時の注意》

本剤は吸湿性が高いので、グラシン紙等防湿効果のない分包材質で調剤した場合は、交付時に取り扱いについて十分注意する旨患者に伝えること。

［参考］

製剤中の水分が7%以上になった場合、ケーキング・変色等の現象を起こしやすい。グラシン紙等に分包した場合は、チャック付きのビニール袋や茶筒等の密閉性の良い容器に入れ、しっかり蓋をして、直射日光をさけ、なるべく湿気の少ない涼しいところに保管する。その際、容器の中に乾燥剤を入れることが望ましい。

15．その他の注意

特になし

16．その他

特になし

Ⅸ. 非臨床試験に関する項目

1．一般薬理

(1)安全性薬理試験

・中枢及び末梢神経系に対する作用

雄性SD系ラットに1000、4000、16000mg/kgを単回経口投与し、Functional Observational Battery（FOB）法にて検討した結果、作用は認められなかった[31]。

・心血管系に対する作用

雄性ビーグル犬に250、1000、4000mg/kgを単回経口投与し、テレメトリー法で検討した結果、作用は認められなかった[32]。

・呼吸系に対する作用

雄性SD系ラットに1000、4000、16000mg/kgを単回経口投与し、Whole Body Plethysmography法で検討した結果、16000mg/kg投与群で呼吸数及び分時換気量の有意な増加が認められた。従って、無作用量は4000mg/kgであると判断された[33]。

2．毒性

⑴単回投与毒性試験[34]

動物種	投与経路	性別	LD_{50} (g/kg)
ddY系マウス	経口	雄	＞15
		雌	＞15
Wistar系ラット	経口	雄	＞15
		雌	＞15

⑵反復投与毒性試験

・SD系ラット雌雄に250、1000、4000mg/kg/日を3カ月間経口投与した結果、毒性学的に意味のある変化は認められなかった[35]。

・ビーグル犬雌雄に250、1000、4000mg/kg/日を3カ月間経口投与した結果、毒性学的に意味のある変化は認められなかった[36]。

⑶生殖発生毒性試験

該当資料なし

⑷その他の特殊毒性

遺伝毒性

細菌を用いる復帰突然変異試験[37]、哺乳類培養細胞を用いる染色体異常試験[38]及びラットを用いる小核試験[39]において、遺伝毒性は認められなかった。

Ⅹ. 取扱い上の注意等に関する項目

1. 有効期間又は使用期限

使用期限：容器、外箱に表示（3年）

設定根拠：安定性試験結果に基づく（自主設定）

2. 貯法・保存条件

薬の品質を保つため、できるだけ湿気をさけ、直射日光のあたらない涼しい所に保管すること。

3. 薬剤取扱い上の注意点

吸湿性が高いため、開封後は特に湿気をさけ、密閉するなど取扱いに注意すること。

4. 承認条件

特になし

5. 包装

500g、5kg（500g×10）、2.5g×42包、2.5g×189包

6. 同一成分・同効薬

［同一処方名薬］

オースギ抑肝散料エキス TG

7. 国際誕生年月日

昭和61年3月5日（製造承認年月日を国際誕生年月日とする）

8. 製造・輸入承認年月日及び承認番号

昭和61年3月5日

（61AM）1133

9. 薬価基準収載年月日

昭和61年10月30日

10. 効能・効果追加、用法・用量変更追加等の年月日及びその内容

該当しない

11. 再審査結果、再評価結果公表年月日及びその内容

該当しない

12．再審査期間

該当しない

13．長期投与の可否

薬剤投与期間の制限を受けない

14．厚生労働省薬価基準収載医薬品コード

5200139D1037

15．保険給付上の注意

特になし

XI. 文献

1．引用文献

1）Takeda, A. et al. Neurochem. Int. 2008, **53**(6-8), p.230.
2）Kawakami, Z. et al. Neuroscience. 2009, **159**(4), p.1397.
3）Egashira, N. et al. Prog. Neuropsychopharmacol. Biol. Psychiatry. 2008, **32**(6), p.1516.
4）Kanno, H. et al. J. Pharm. Pharmacol. 2009, **61**(9), p.1249.
5）Nishi, A. et al. Neuroscience. 2012, **207**, p.124.
6）Terawaki, K. et al. J. Ethnopharmacol. 2010, **127**(2), p.306.
7）Yamaguchi, T. et al. J. Ethnopharmacol. 2012, **143**(2), p.533.
8）栗原　久・他. 神経精神薬理. 1996, **18**(3), p.179.
9）Nogami, A. et al. J. Nat. Med. 2011, **65**(2), p.275.
10）Mizoguchi, K. et al. J. Ethnopharmacol. 2010, **127**(1), p.70.
11）Fujiwara, H. et al. Neuroscience. 2011, **180**, p.305.
12）Sekiguchi, K. et al. Phytother. Res. 2009, **23**(8), p.1175.
13）Tamano, H. et al. Brain. Res. Bull. 2010, **83**(6), p.351.
14）Egashira, N. et al. J. Pharmacol. Sci. 2011, **116**(3), p.316.
15）株式会社ツムラ社内資料
16）Ishida, S. et al. Chem. Pharm. Bull. 1988, **36**(1), p.440.
17）株式会社ツムラ社内資料
18）株式会社ツムラ社内資料
19）Shimizu, K. et al. J. Pharmacobio-Dyn. 1985, **8**, p.718.
20）Hattori, M. et al. Planta Med. 1983, **48**, p.38.
21）山田光胤. 漢方処方応用の実際. 南山堂, 1977, p.21.
22）松田邦夫・他. 臨床医のための漢方［基礎編］. カレントテラピー, 1989, p.30.
23）菊谷豊彦. 日本薬剤師会雑誌. 1982, **34**(8), p.727.
24）菊谷豊彦. 大阪医薬品協会会報. 1984, (6), p.1.
25）日本医師会編. 漢方治療のABC. 医学書院, 1992, p.14.
26）張　明澄. 中国医学薬物事典. エンタプライズ, 1990, p.125.
27）松田邦夫・他. 日本東洋医学雑誌. 1988, **38**(4), p.15.
28）久田孝光・他. 診断と治療. 2014, **102**(10), p.1577.
29）塩之入　洋・他. 臨床水電解質. 1985, **4**(2), p.184.
30）森本靖彦・他. 和漢医薬学会誌. 1991, **8**(1), p.1.
31）株式会社ツムラ社内資料
32）株式会社ツムラ社内資料
33）株式会社ツムラ社内資料
34）株式会社ツムラ社内資料
35）株式会社ツムラ社内資料

36）株式会社ツムラ社内資料
37）株式会社ツムラ社内資料
38）株式会社ツムラ社内資料
39）株式会社ツムラ社内資料

2．その他の参考文献

特になし

XII．参考資料

主な外国での発売状況

2020年11月現在、外国では発売されていない。

XIII．備考
