# Supplementary material for: Effects of the Kampo medicine Yokukansan for perioperative anxiety and postoperative pain in women undergoing breast surgery: A randomized, controlled trial
Source: PLoS One. 2021 Nov 24;16(11):e0260524. doi: 10.1371/journal.pone.0260524 (PMC8612547; doi:10.1371/journal.pone.0260524)
Supplement: Attachment — Submitted filename: 210531 Kainuma Reviewers comments for PLOS one.pdf [file pone.0260524.s008.pdf]

The authors have shown the efficacy of yokukansan on anxiety using both subjective and objective rating scales. We think that it is very interesting study; however, the problems listed below will need to be addressed before the paper is suitable for publication.

Major points

Although the authors used changes in sAA, HADS, and STAI as primary outcomes, it would be best to use only one thing as the primary outcome. Further, the sample size was for sAA in this study. If you decide on a primary outcome other than sAA, the sample size will become quite different.

The authors planned for yokukansan to be taken two times before sleep on the night before surgery and 2h before the induction of anesthesia. Was the duration of the first two times the same in this study? Further, why did you decide to take yokukansan 2hr before the induction of anesthesia?

Minor points

1. In the analysis of HADS, is there a statistically significant difference between the two groups over time?
2. The figure legends should be written after the references, please check that the formatting follows the journal's instructions for authors.
