# Supplementary material for: Effects of the Kampo medicine Yokukansan for perioperative anxiety and postoperative pain in women undergoing breast surgery: A randomized, controlled trial
Source: PLoS One. 2021 Nov 24;16(11):e0260524. doi: 10.1371/journal.pone.0260524 (PMC8612547; doi:10.1371/journal.pone.0260524)
Supplement: Attachment — Submitted filename: Response to Reviewers.docx [file pone.0260524.s009.docx]

Reviewer's Responses to Questions

6. Review Comments to the Author

Please use the space provided to explain your answers to the questions above. You may also include additional comments for the author, including concerns about dual publication, research ethics, or publication ethics. (Please upload your review as an attachment if it exceeds 20,000 characters)

Reviewer #1: In the discussion with Reviewers, the authors revised as follows.

Changes in sAA were considered as primary outcomes, and those in HADS, STAI, QoR-15, and VAS were considered as secondary outcomes.

However, in the UMIN registration, the authors declare that Primary outcomes as “subjective evaluation items: HADS　(Hospital anxiety and depression scale)” and “objective evaluation items: salivary amylase activity” and Secondary outcomes as “STAI (State Trait Anxiety Inventory)”, QoR (quality of recovery) score”, and “VAS score of pain”.

This study is designed clinical trial, if you change this point, amendment of trial design is needed.

Response

We thank reviewer #1 for pointing out the ambiguity in the manuscript. In this clinical study, we needed re-registration with the Japan Registry of Clinical Trials (jRCT). In the modified registry, we set the value of sAA (objective outcome of perioperative anxiety) as the primary outcome, and questionnaire-assessed anxiety and perioperative pain (HADS, STAI, QoR-15, and VAS) were set as secondary outcomes. The jRCT has been reviewed by the secretariat of the Central Clinical Research Review Committee of Niigata University, which is a third-party organization and has a stricter protocol. This research was conducted in accordance with the research protocol registered in jRCT, which is generally consistent with the research protocol registered in UMIN.

We have added the description about registration in jRCT and described primary and secondary outcomes in the materials and methods section (P8L109 – P9L113).

Reviewer #2: (No Response)

Reviewer #3: Major points

・Although the authors used changes in sAA, HADS, and STAI as primary outcomes, it would be best to use only one thing as the primary outcome. Further, the sample size was for sAA in this study. If you decide on a primary outcome other than sAA, the sample size will become quite different.

Response: Thank you for your the valuable suggestion. Accordingly, we set sAA as the primary outcome and questionnaire results as a secondary outcome. We calculated the sample size using differences in sAA as already shown in the sample size calculation (P14 L191-195).

OK

・The authors planned for yokukansan to be taken two times before sleep on the night before surgery and 2h before the induction of anesthesia. Was the duration of the first two times the same in this study? Further, why did you decide to take yokukansan 2hr before the induction of anesthesia?

Response: In a previous study (Evid Based Complement Alternat Med. 2014;2014: 965045, ref. #12), Yokukansan was administered once 5 hours before surgery. However, in cases where surgery is conducted in the morning, YKS was orally administered very early in the morning, two hours before prohibiting water. However, since the previous study used drugs that were non-inferior to benzodiazepines, we aimed to administer a slightly higher dose of Yokukansan to clarify its effect in this superiority study. Nevertheless, since prolonging the duration of medication would increase the number of dropouts, we started administration of yokukansan after hospital admission of patients to ensure that the medication was administered twice.

OK

Minor points

1 In the analysis of HADS, is there a statistically significant difference between the two groups over time?

Response: We found no significant differences in the main effect, but there were significant differences in the interaction.

OK

2 The figure legends should be written after the references, please check that the formatting follows the journal’s instructions for authors.

The journal guidelines verify that figure legends should be included immediately after the citation of the corresponding figure in the text. Please see the corresponding guidelines below:

Response: Figure captions have been inserted immediately after the first paragraph whereeach figure is cited as mentioned in the submission guidelines. Figure files are uploaded separately.

OK

The author responded appropriately to reviewers’ comments.

Response: We thank reviewer #3 for the effort in assessing our rebuttal letter.
